# Supplementary material for: Bixbyite-type Ln2O3 as promoters of metallic Ni for alkaline electrocatalytic hydrogen evolution
Source: Nat Commun. 2022 Jul 5;13:3857. doi: 10.1038/s41467-022-31561-4 (PMC9256667; doi:10.1038/s41467-022-31561-4)
Supplement: Supplementary file 1 — Supplementary Information [file 41467_2022_31561_MOESM1_ESM.pdf]

Supplementary Information

Bixbyite-type Ln_2O_3 as promoters of metallic Ni for alkaline electrocatalytic hydrogen evolution

Hongming Sun[1,+], Zhenhua Yan[2,+], Caiying Tian[1,+], Cha Li[1,+], Xin Feng[1], Rong Huang[1], Yinghui Lan[1], Jing Chen[1], Cheng-Peng Li[1*], Zhihong Zhang[3] & Miao Du[1,3*]

[1] College of Chemistry, Tianjin Key Laboratory of Structure and Performance for Functional Molecules, Tianjin Normal University, Tianjin 300387, China

[2] Key Laboratory of Advanced Energy Materials Chemistry (Ministry of Education), College of Chemistry, Nankai University, Tianjin 300071, China

[3] College of Materials and Chemical Engineering, Zhengzhou University of Light Industry, Zhengzhou 450001, China

[+] These authors contributed equally

*e-mail: hxxylcp@tjnu.edu.cn; hxxydm@tjnu.edu.cn

Experimental section

Chemicals. Ethanol (AR, CAS no. 64-17-5) and nickel nitrate (≥ 98%, CAS no. 13478-00-7) were purchased from Tianjin Damao Chemical Trading Co., Ltd. Potassium hydroxide (95%, CAS no. 1310-58-3), ytterbium nitrate pentahydrate (99.9%, CAS no. 35725-34-9), thulium nitrate hexahydrate (99.9%, CAS no. 36548-87-5), cerium nitrate hexahydrate (99.9%, CAS no. 10294-41-4), gadolinium nitrate hydrate (99.9%, CAS no. 94219-55-3), samarium nitrate hexahydrate (99.9%, CAS no. 13759-83-6), and europium nitrate hexahydrate (99.9%, CAS no. 10031-53-5) were obtained from Shanghai Macklin Biochemical Co., Ltd. Lutetium nitrate hexahydrate (99.9%, CAS no. 10099-67-9), erbium nitrate pentahydrate (99.9%, CAS no. 10031-51-3), holmium nitrate pentahydrate (99.9%, CAS no. 14483-18-2), and dysprosium nitrate pentahydrate (99.9%, CAS no. 10031-49-9) were purchased from Aladdin. Pt/C (20%, CAS no. 7440-06-4) was purchased from Meryer (Shanghai) Chemical Technology Co., Ltd. Hydrochloric acid (36~ 38%, CAS no. 7647-01-0) and Nafion (D-521 dispersion 5% w/w in water & 1-propanol, ≥ 0.92 meq g^{-1} exchange, CAS no. 31175-20-9) was purchased from Alfa Aesar. H_2 gas (99.99%) and Ar gas (99.999%) were purchased from Huanyu Co., Ltd. All chemicals were used as received without further purification.

Electrical conductivity tests. To measure the electrical resistivity, the powder catalysts were scraped off the self-supported electrodes. Around 100 mg sample was pressed by 20 Mpa into a 0.50 cm diameter, 0.05 cm thick chip. Then two stainless steel electrodes (2.0 cm diameter, 0.2 cm thick) were placed onto two faces of the sample chip and held by an alligator chip. The resistivity test was taken using the Bipotentiostat workstation (Pine Research Instrumentation,

Basic Wave Driver 20 Bundle, USA). The resistance values (R) of the samples were calculated by voltmeter-ammeter method[1]. The conductivity k was deduced using the relation: $\kappa = 1/\rho = h / (R \times S)$, where ρ is resistivity and S and h are the surface and thickness of the chips.

Turnover frequency (TOF) calculation. The per-site TOF values can be calculated based on the following equation.

$$\text{TOF} = \frac{\#\ \text{total hydrogen turnover} / \text{cm}^2\ \text{geometric area}}{\#\ \text{active sites} / \text{cm}^2\ \text{geometric area}} \qquad (1)$$

The number of total hydrogen turnovers is calculated from the current density according to:

$$\#H_2 = \left(j\ \frac{\text{mA}}{\text{cm}^2}\right)\left(\frac{1\text{C/s}}{1000\text{mA}}\right)\left(\frac{1\text{mol}\ e^-}{96485.3\ \text{C}}\right)\left(\frac{1\text{mol}\ H_2}{2\text{mol}\ e^-}\right)\left(\frac{6.02 \times 10^{23}\ \text{molecules}\ H_2}{1\text{mol}\ H_2}\right) = 3.12 \times 10^{15}\ \frac{H_2/s}{\text{cm}^2}\ \text{per}\ \frac{\text{mA}}{\text{cm}^2} \qquad (2)$$

Given the great difficulty of determining the exact quantity of active sites on catalyst surface, especially for such hybrid catalysts with interface in this work, we assume the total number of surface Ni sites as the number of active sites, which are estimated by the reported method[2]. Molar mass of Ni: 58.69 g mol^{-1}; Density of Ni: 8.90 g cm^{-3}; Molar volume of Ni: 6.60 $\text{cm}^3\ \text{mol}^{-1}$. Average surface atoms per 1 square centimeter:

$$\#\text{surface sites} = \left(\frac{1\ \times 6.022 \times 10^{23}\ \text{atoms}}{1\ \text{mol}} \times \frac{1\ \text{mol}}{6.60\ \text{cm}^3}\right)^{\frac{2}{3}} = 2.03 \times 10^{15}\ \frac{\text{atoms}}{\text{cm}^2} \qquad (3)$$

Then, the current density from the LSV polarization curves can be converted into TOF values according to:

$$\text{TOF} = \frac{\left(3.12\times10^{15}\frac{H_2/s}{\text{cm}^2}\ \text{per}\ \frac{\text{mA}}{\text{cm}^2}\right)\times|j|}{(\#\text{surface sites}) \times A_{ECSA}} \qquad (4)$$

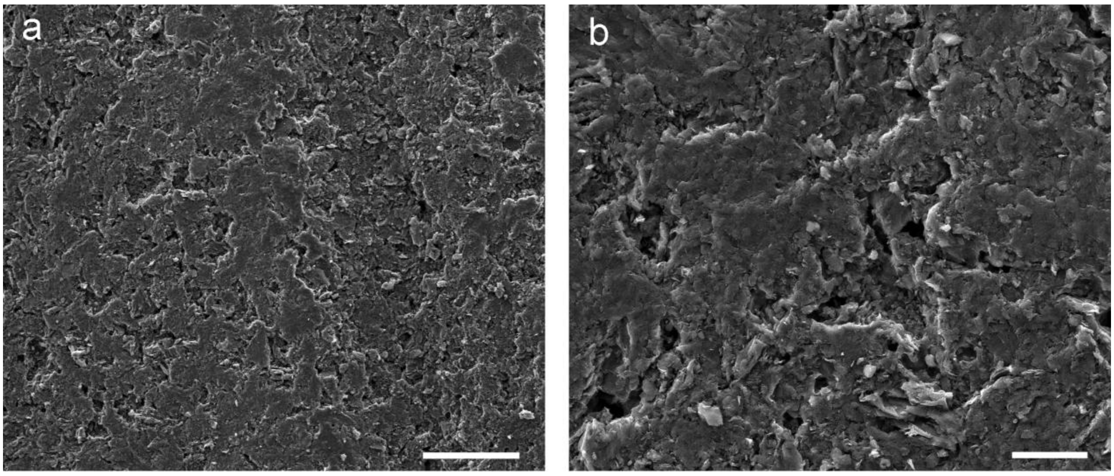

Supplementary Figure 1. Morphology characterizations of the graphite plate without any catalyst. a Low-magnification SEM image (scale bar: 100 μm). **b** high-magnification SEM image (scale bar: 20 μm).

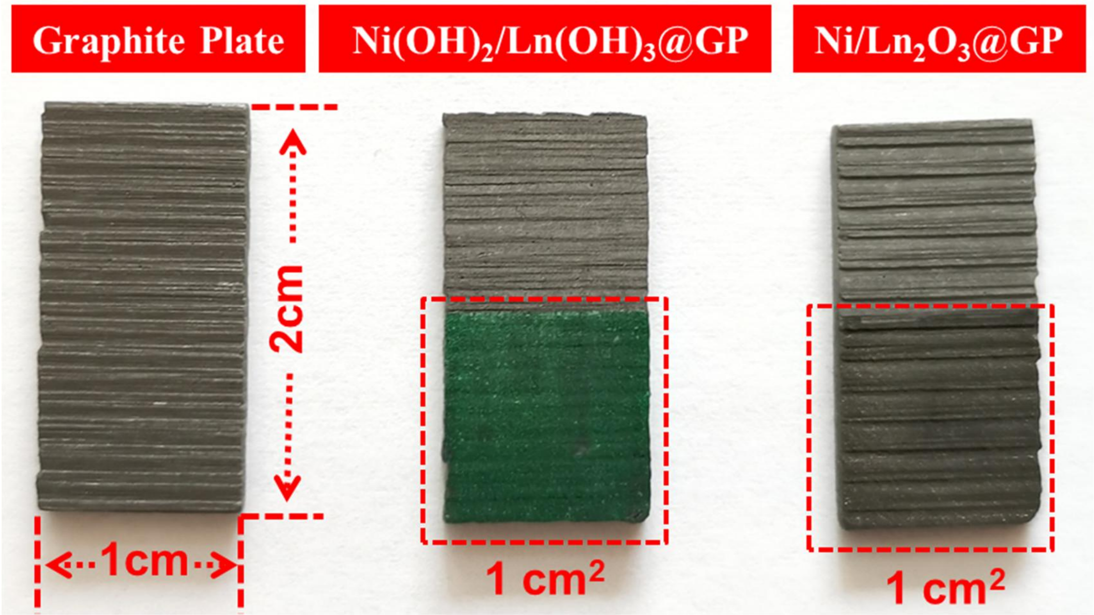

Supplementary Figure 2. Optical images of electrodes. (Left) graphite plate (GP), (middle) $Ni(OH)_2/Ln(OH)_3$@GP and (right) Ni/Ln_2O_3@GP electrodes.

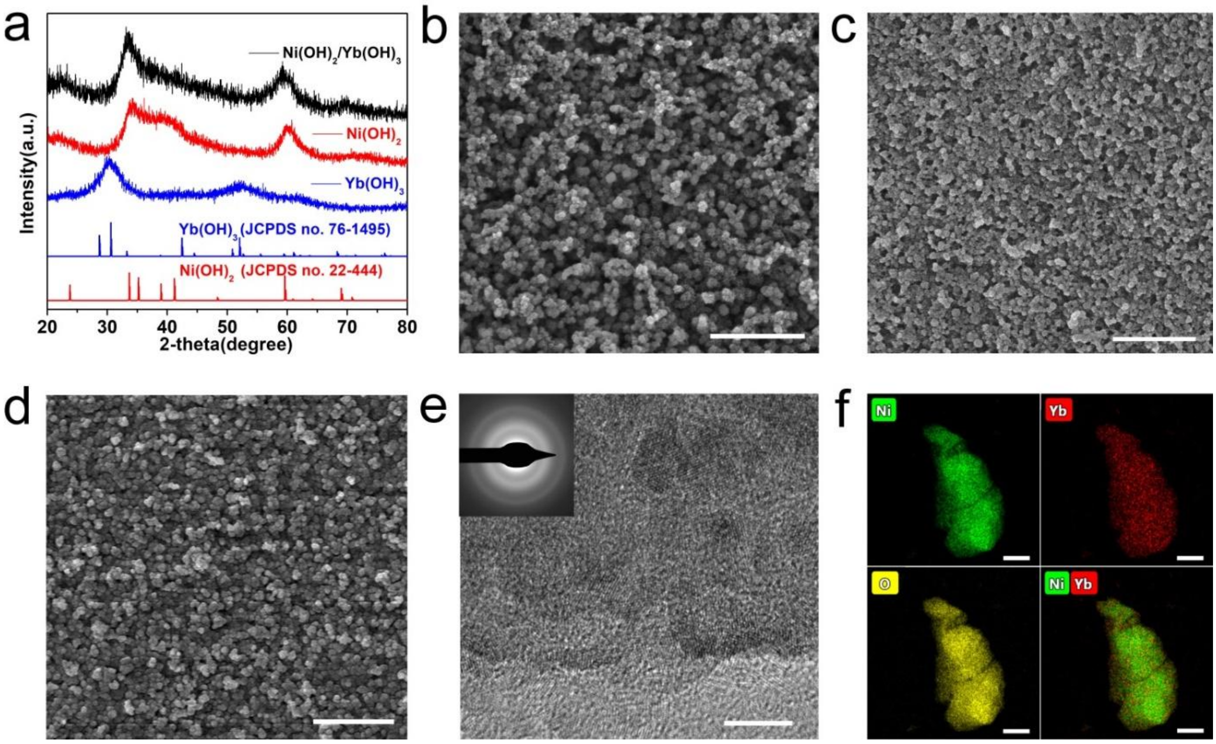

Supplementary Figure 3. Characterizations of $Ni(OH)_2$, $Yb(OH)_3$ and $Ni(OH)_2/Yb(OH)_3$ precursor electrodes. a XRD patterns of Ni(OH), $Yb(OH)_3$, and $Ni(OH)_2/Yb(OH)_3$. **b** SEM image of $Ni(OH)_2$@GP (scale bar: 2 μm). **c** SEM image of $Yb(OH)_3$@GP (scale bar: 1 μm). **d** SEM image of $Ni(OH)_2/Yb(OH)_3$@GP (scale bar: 2 μm). **e** TEM image of $Ni(OH)_2/Yb(OH)_3$ (scale bar: 5 nm; inset: SAED pattern). **f** TEM-EDX elemental mapping of $Ni(OH)_2/Yb(OH)_3$ (scale bar: 100 nm).

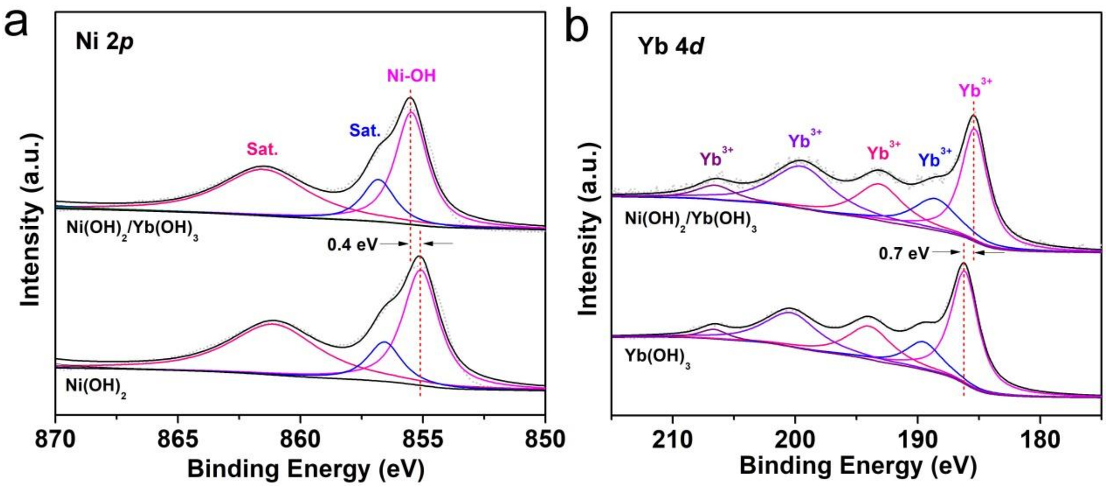

Supplementary Figure 4. XPS spectra of $Ni(OH)_2$, $Yb(OH)_3$ and $Ni(OH)_2/Yb(OH)_3$. a Ni 2*p* XPS spectra of $Ni(OH)_2$ and $Ni(OH)_2/Yb(OH)_3$. **b** Yb 4*d* XPS spectra of $Yb(OH)_3$ and $Ni(OH)_2/Yb(OH)_3$. For Ni 2*p* XPS spectrum of $Ni(OH)_2/Yb(OH)_3$, the poignant peak at 855.5 eV is indexed to Ni^{2+} ion[3], which is 0.4 eV higher relative to that of $Ni(OH)_2$. For Yb 4*d* XPS spectrum of $Ni(OH)_2/Yb(OH)_3$, the main peak at 185.5 eV corresponds to Yb^{3+} ion[4], which shows a 0.7 eV negative shift compared with that of $Yb(OH)_3$. The XPS results indicate the electron transfer between $Ni(OH)_2$ and $Yb(OH)_3$.

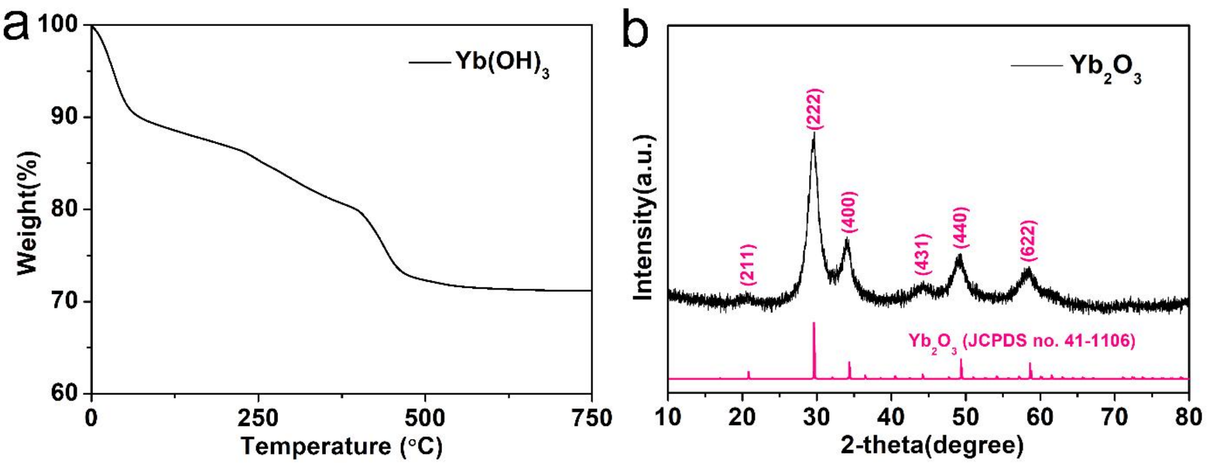

Supplementary Figure 5. Thermostability of $Yb(OH)_3$. a Thermogravimetric analysis curve of $Yb(OH)_3$ powder in argon atmosphere with a ramping rate of 5 °C min^{-1}. **b** XRD pattern of the sample obtained from $Yb(OH)_3$ after thermogravimetric analysis. This result suggests that $Yb(OH)_3$ will decompose into Yb_2O_3 under 500 °C during sintering.

Supplementary Table 1. The $\Delta_f H_m^\theta$ and S_m^θ of reactants for preparing Ni and Ni/Yb_2O_3.

Reactant	H_2	H_2O	$Ni(OH)_2$	Ni	Yb_2O_3	Yb
$\Delta_f H_m^\theta$ (kJ/mol)	0	-241.8	-529.7	0	-1814.6	0
S_m^θ (J/mol K)	130.7	188.8	88.0	29.9	149.8	74.8

The temperatures of reduction reactions from $Ni(OH)_2$ and Yb_2O_3 to metal Ni and Yb were calculated from the point of thermodynamics ($\Delta_r G_m^\theta = \Delta_r H_m^\theta - T\Delta_r S_m^\theta$).

$$\mathbf{Ni(OH)_2(s) + H_2(g) = Ni(s) + 2H_2O(g)} \quad (5)$$

$$\Delta_r H_m^\theta = \Delta_f H_m^\theta\ (\text{Ni,s}) + 2\Delta_f H_m^\theta\ (\text{H}_2\text{O,g}) - \Delta_f H_m^\theta\ (\text{H}_2\text{,g}) - \Delta_f H_m^\theta\ (\text{Ni(OH)}_2\text{,s})$$

$$= 0 + 2 \times (-241.8\ \text{kJ/mol}) - 0 - (-529.7\ \text{kJ/mol}) \qquad = 46.1\ \text{kJ/mol}$$

$$\Delta_r S_m^\theta = S_m^\theta\ (\text{Ni,s}) + 2S_m^\theta\ (\text{H}_2\text{O,g}) - S_m^\theta\ (\text{H}_2\text{,g}) - S_m^\theta\ (\text{Ni(OH)}_2\text{,s})$$

$$= 29.9\ \text{J/mol K} + 2 \times 188.8\ \text{J/mol K} - 130.7\ \text{J/mol K} - 88.0\ \text{J/mol K}$$

$$= 188.8\ \text{J/mol K}$$

$$T = 46.1 \times 10^3/188.8 = 244.17\ \text{K}$$

$$\mathbf{Yb_2O_3(s) + 3H_2(g) = 2Yb(s) + 3H_2O(g)} \quad (6)$$

$$\Delta_r H_m^\theta = 2\Delta_f H_m^\theta\ (\text{Yb,s}) + 3\Delta_f H_m^\theta\ (\text{H}_2\text{O,g}) + 3\Delta_f H_m^\theta\ (\text{H}_2\text{,g}) - \Delta_f H_m^\theta\ (\text{Yb}_2\text{O}_3\text{,s})$$

$$= 0 + 3 \times (-241.8\ \text{kJ/mol}) - 0 - (-1814.6\ \text{kJ/mol}) = 1089.2\ \text{kJ/mol}$$

$$\Delta_r S_m^\theta = S_m^\theta\ (\text{Yb,s}) + 3S_m^\theta\ (\text{H}_2\text{O,g}) - 3S_m^\theta\ (\text{H}_2\text{,g}) - S_m^\theta (\text{Yb}_2\text{O}_3\text{,s})$$

$$= 74.8\ \text{J/mol K} + 3 \times 188.8\ \text{J/mol K} - 3 \times 130.7\ \text{J/mol K} - 149.8\ \text{J/mol K}$$

$$= 99.3\ \text{J/mol K}$$

$$T = 1089.2 \times 10^3/99.3 = 10968.8\ \text{K}$$

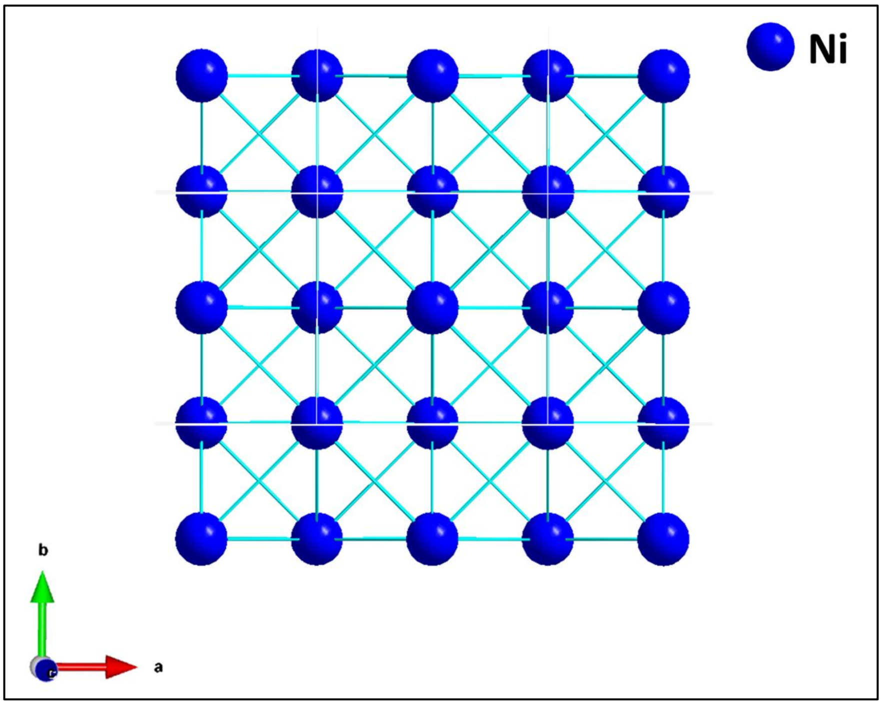

Supplementary Figure 6. Crystal structure. Crystal structure of cubic Ni.

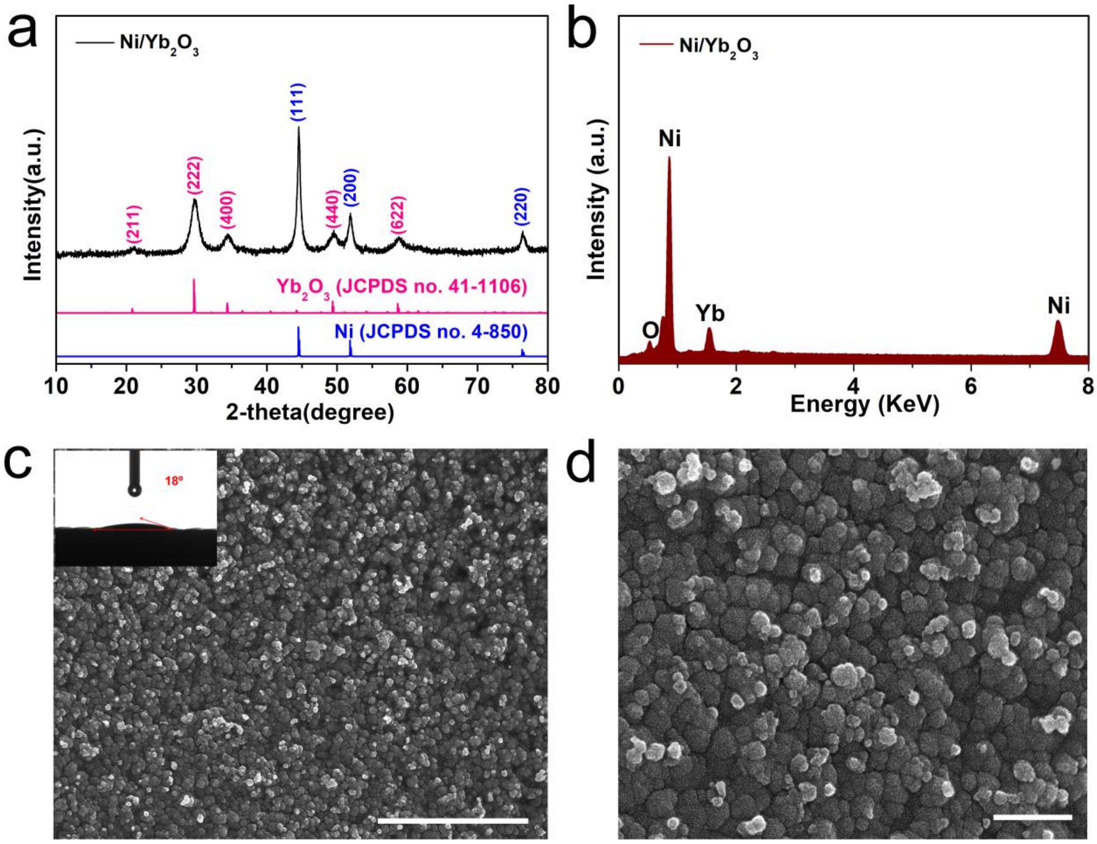

Supplementary Figure 7. Characterization of Ni/Yb_2O_3. a XRD pattern. **b** EDS curve. **c, d** SEM images. Inset of **c** shows the wetting ability test, indicating that Ni/Yb_2O_3 is hydrophilic. Scale bar: **c** 3 μm, **d** 500 nm. The Ni:Yb molar ratio is 90.78:9.22.

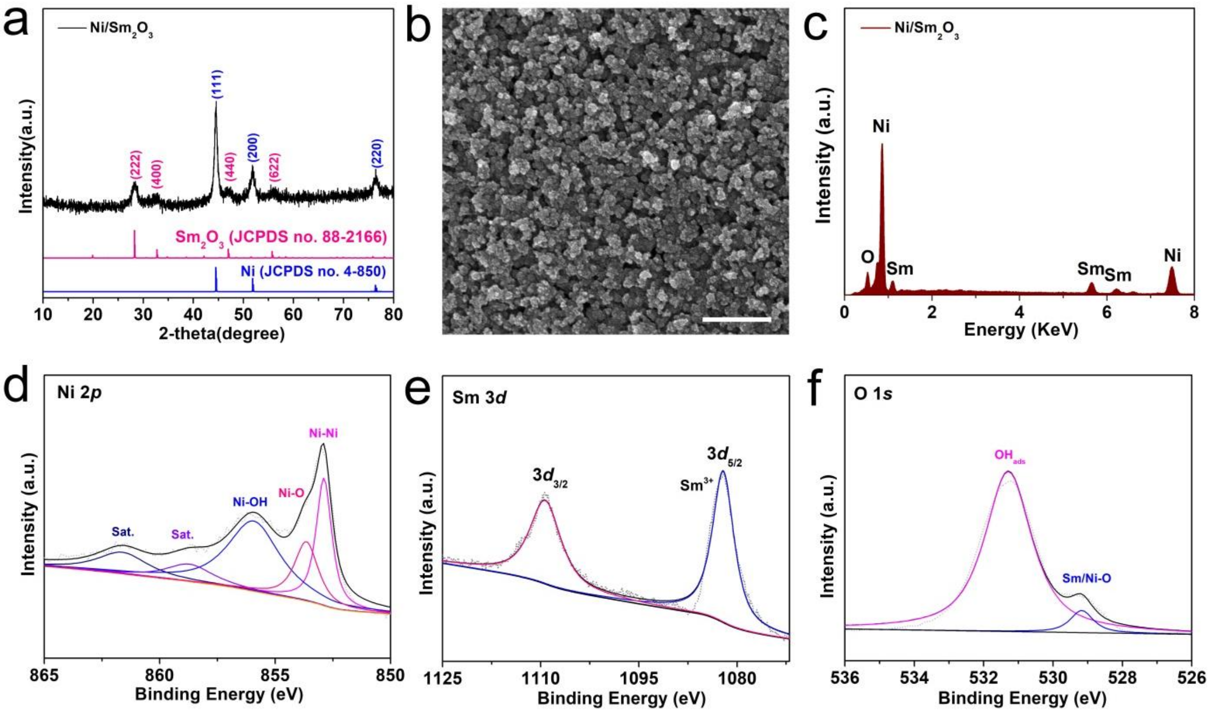

Supplementary Figure 8. Characterization of Ni/Sm_2O_3. a XRD pattern. **b** SEM image (scale bar: 500 nm). **c** EDS spectrum. **d** Ni 2*p* XPS spectrum. **e** Sm 3*d* XPS spectrum. **f** O 1*s* XPS spectrum. The Ni:Sm molar ratio is 90.54:9.46.

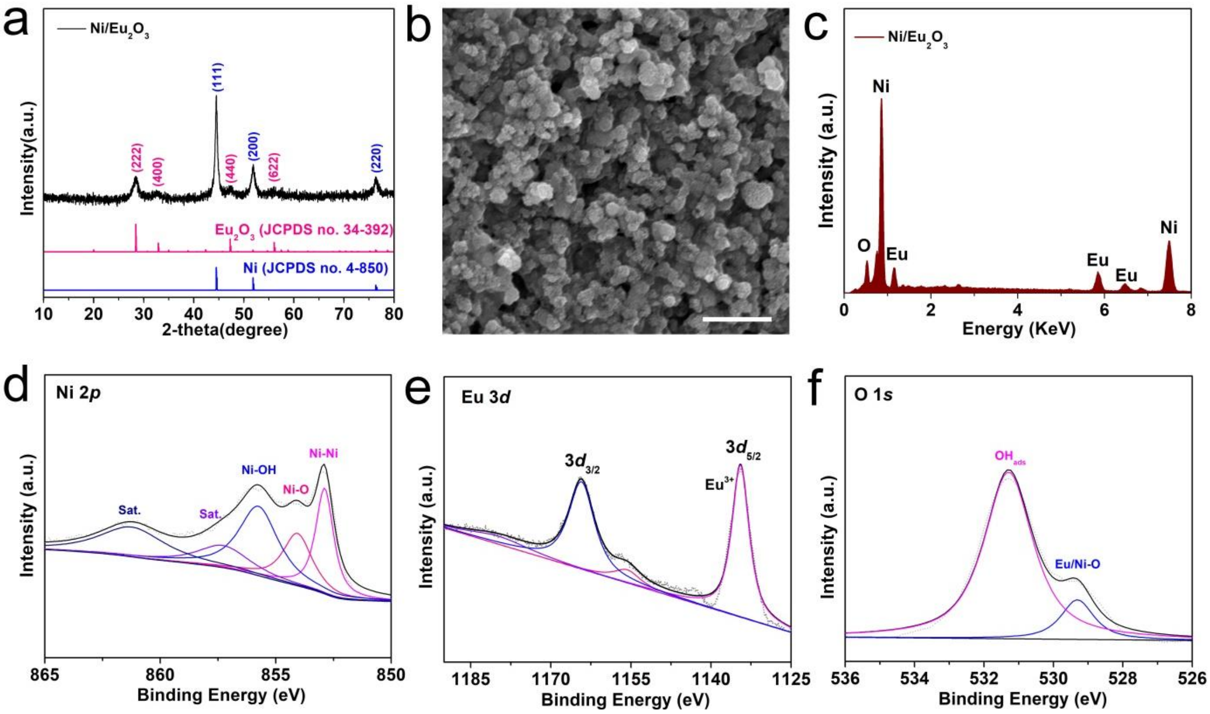

Supplementary Figure 9. Characterization of Ni/Eu_2O_3. a XRD pattern. **b** SEM image (scale bar: 500 nm). **c** EDS spectrum. **d** Ni 2*p* XPS spectrum. **e** Eu 3*d* XPS spectrum. **f** O1*s* XPS spectrum. The Ni:Eu molar ratio is 89.16:10.84.

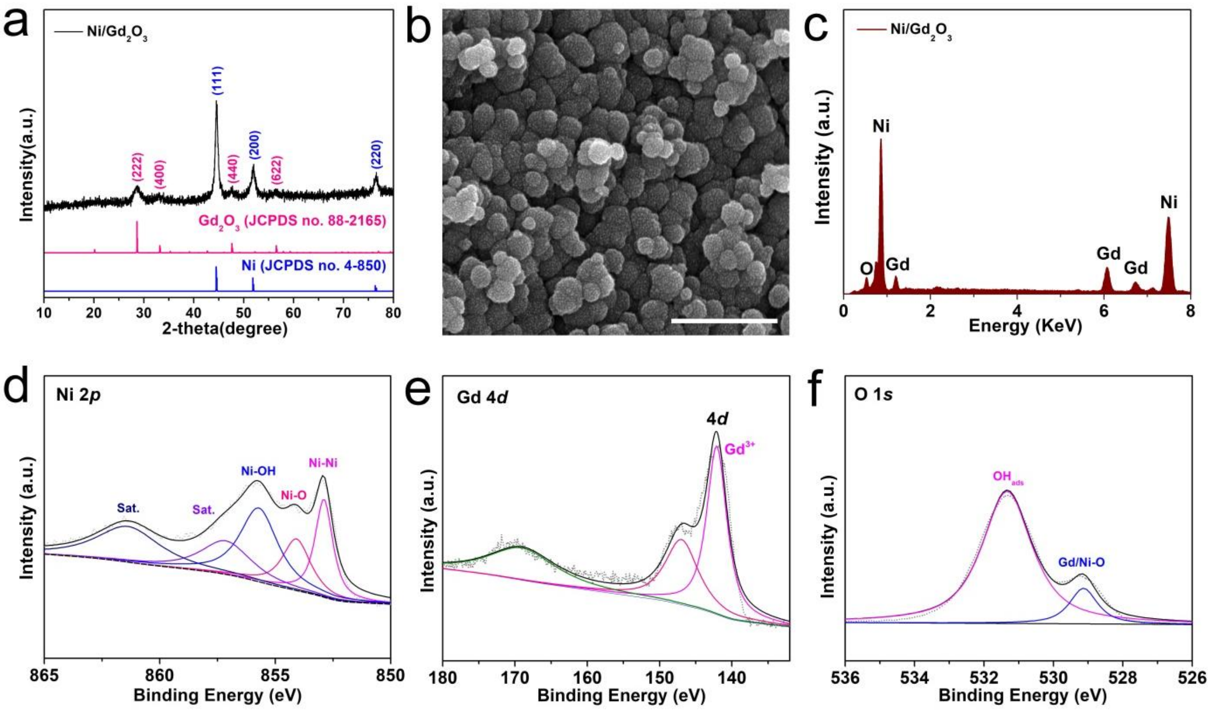

Supplementary Figure 10. Characterization of Ni/Gd_2O_3. a XRD pattern. **b** SEM image (scale bar: 500 nm). **c** EDS spectrum. **d** Ni 2*p* XPS spectrum. **e** Gd 4*d* XPS spectrum. **f** O 1*s* XPS spectrum. The Ni:Gd molar ratio is 90.47:9.53.

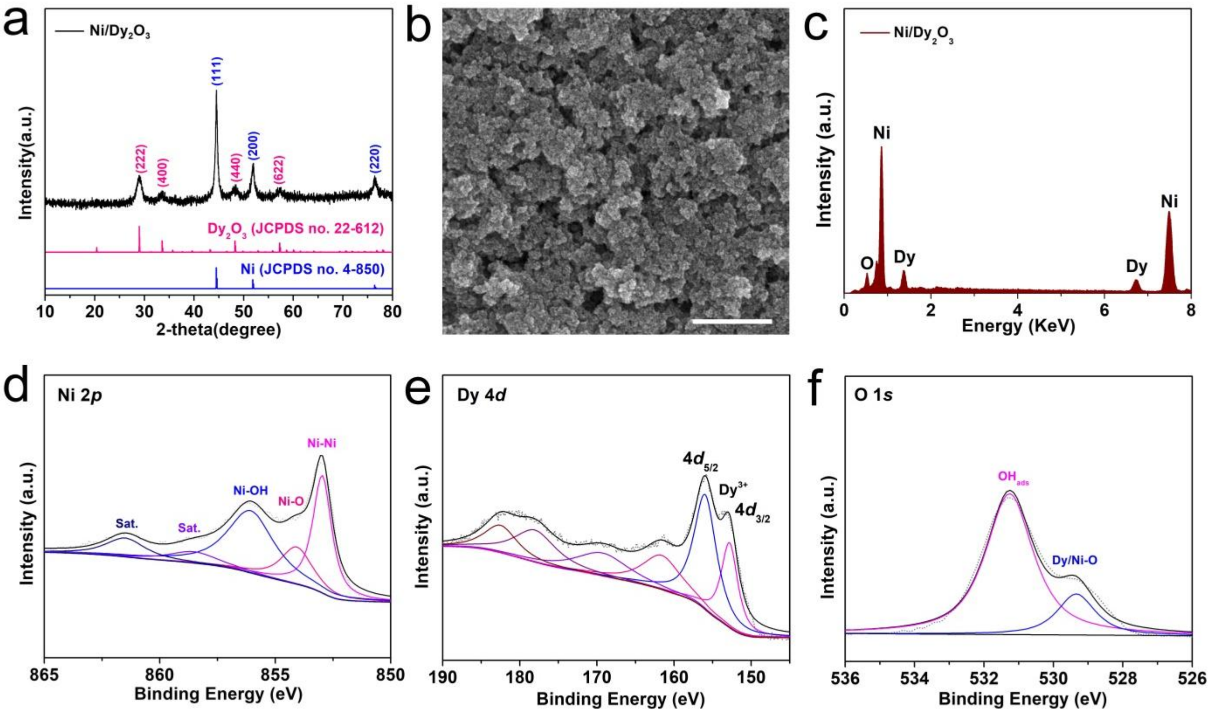

Supplementary Figure 11. Characterization of Ni/Dy_2O_3. a XRD pattern. **b** SEM image (scale bar: 500 nm). **c** EDS spectrum. **d** Ni 2*p* XPS spectrum. **e** Dy 4*d* XPS spectrum. **f** O 1*s* XPS spectrum. The Ni:Dy molar ratio is 89.59:10.41.

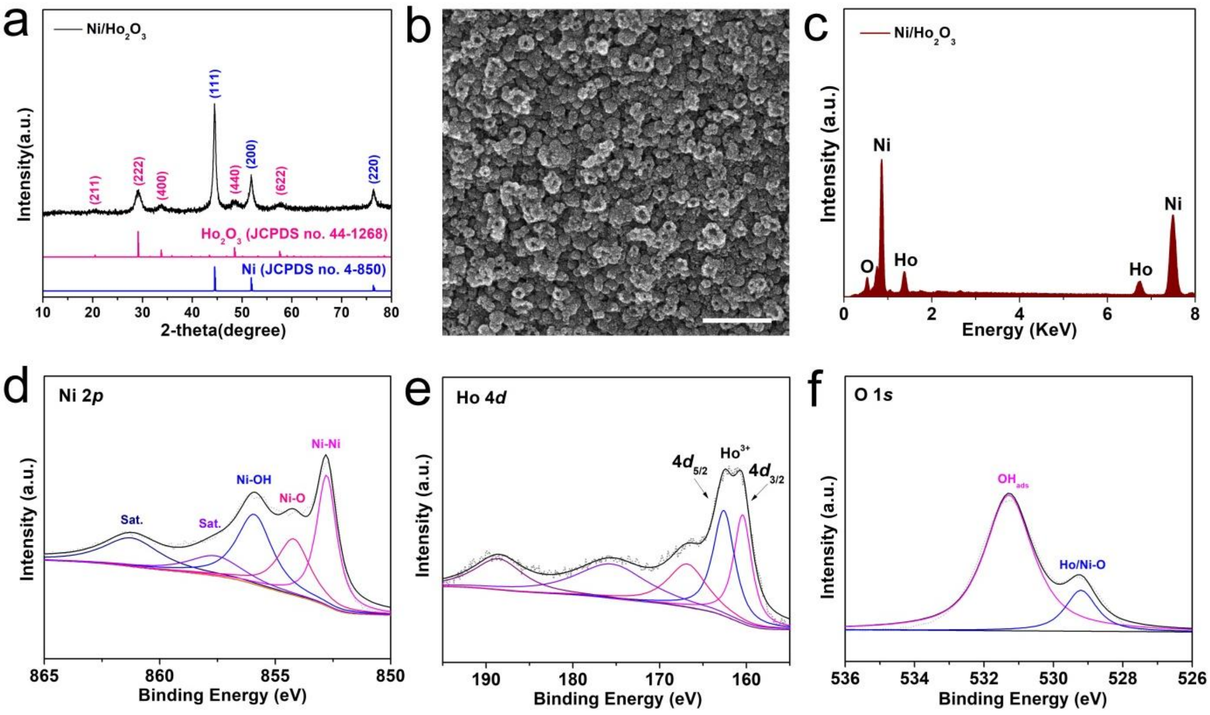

Supplementary Figure 12. Characterization of Ni/Ho_2O_3. a XRD pattern. **b** SEM image (scale bar: 500 nm). **c** EDS spectrum. **d** Ni 2*p* XPS spectrum. **e** Ho 4*d* XPS spectrum. **f** O 1*s* XPS spectrum. The Ni:Ho molar ratio is 88.11:11.89.

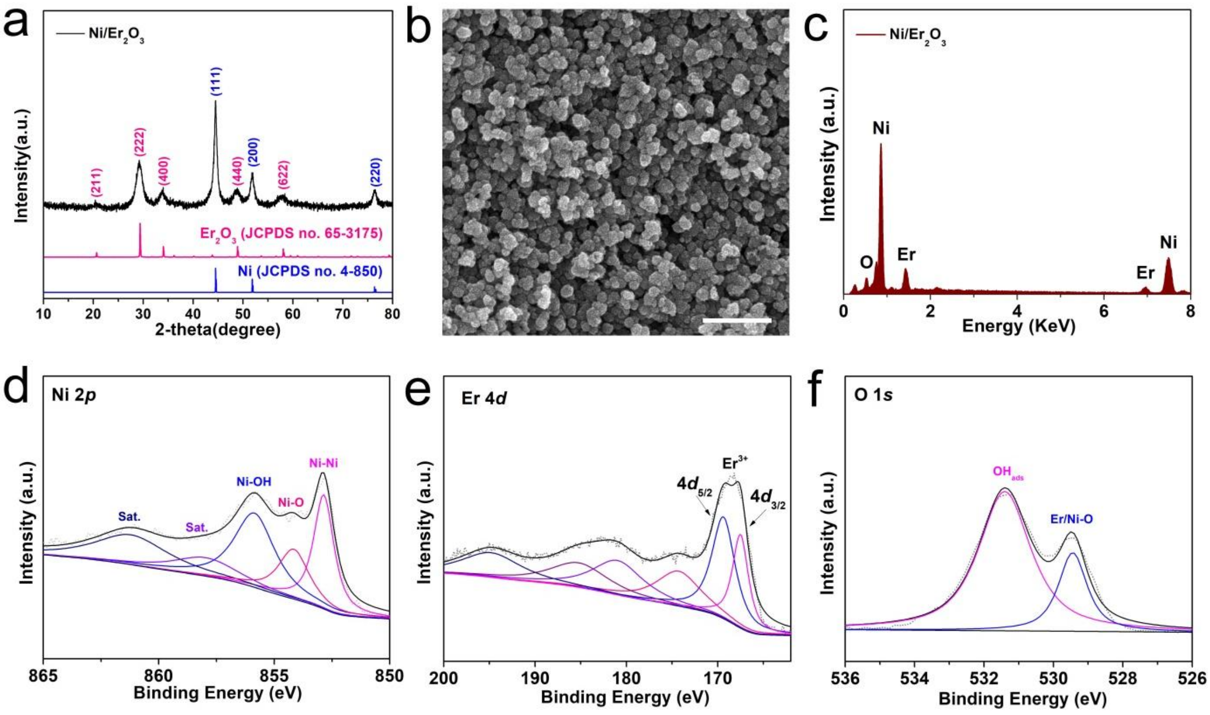

Supplementary Figure 13. Characterization of Ni/Er_2O_3. a XRD pattern. **b** SEM image (scale bar: 500 nm). **c** EDS spectrum. **d** Ni 2*p* XPS spectrum. **e** Er 4*d* XPS spectrum. **f** O 1*s* XPS spectrum. The Ni:Er molar ratio is 89.82:10.18.

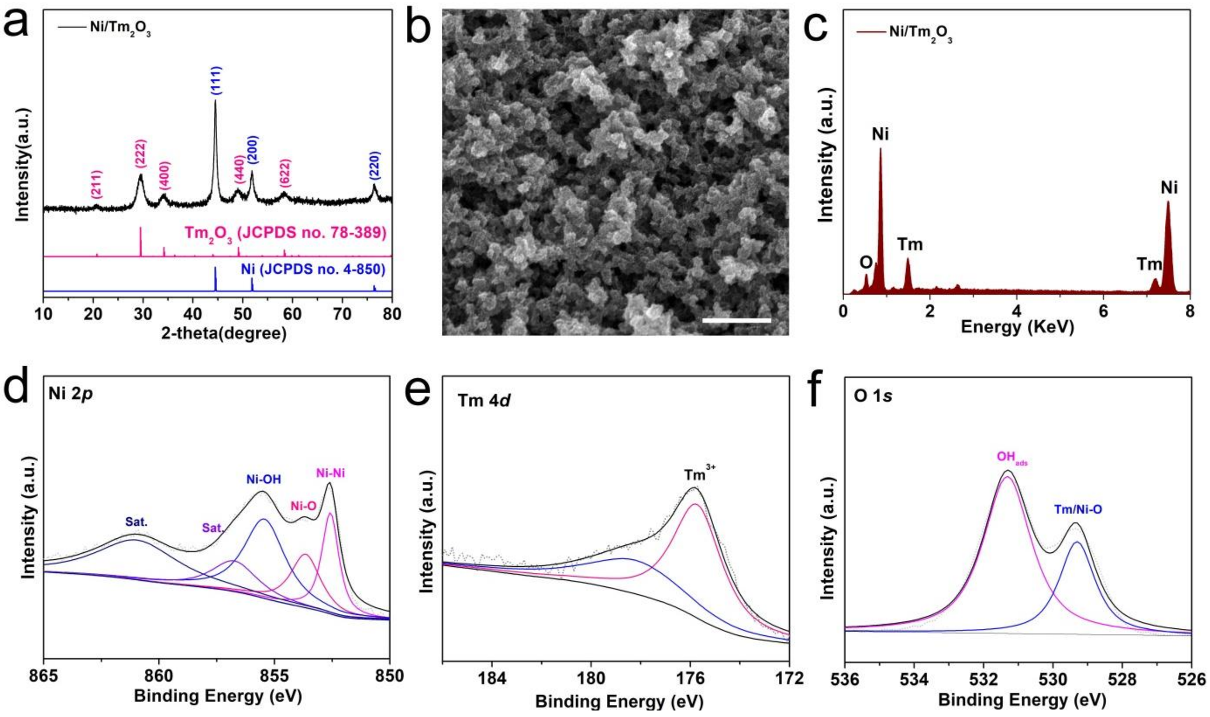

Supplementary Figure 14. Characterization of Ni/Tm_2O_3. a XRD pattern. **b** SEM image (scale bar: 500 nm). **c** EDS spectrum. **d** Ni 2*p* XPS spectrum. **e** Tm 4*d* XPS spectrum. **f** O 1*s* XPS spectrum. The Ni:Tm molar ratio is 88.08:11.92.

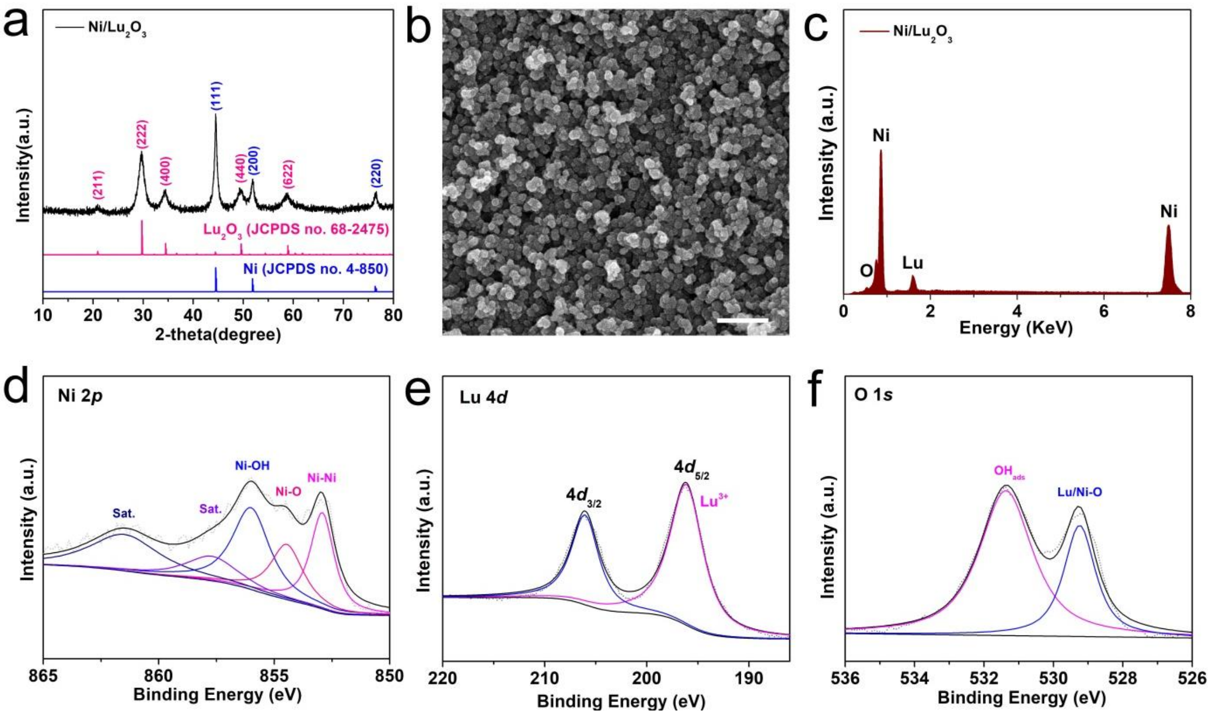

Supplementary Figure 15. Characterization of Ni/Lu_2O_3. a XRD pattern. **b** SEM image (scale bar: 500 nm). **c** EDS spectrum. **d** Ni 2*p* XPS spectrum. **e** Lu 4*d* XPS spectrum. **f** O 1*s* XPS spectrum. The Ni:Sm molar ratio is 90.00:10.00.

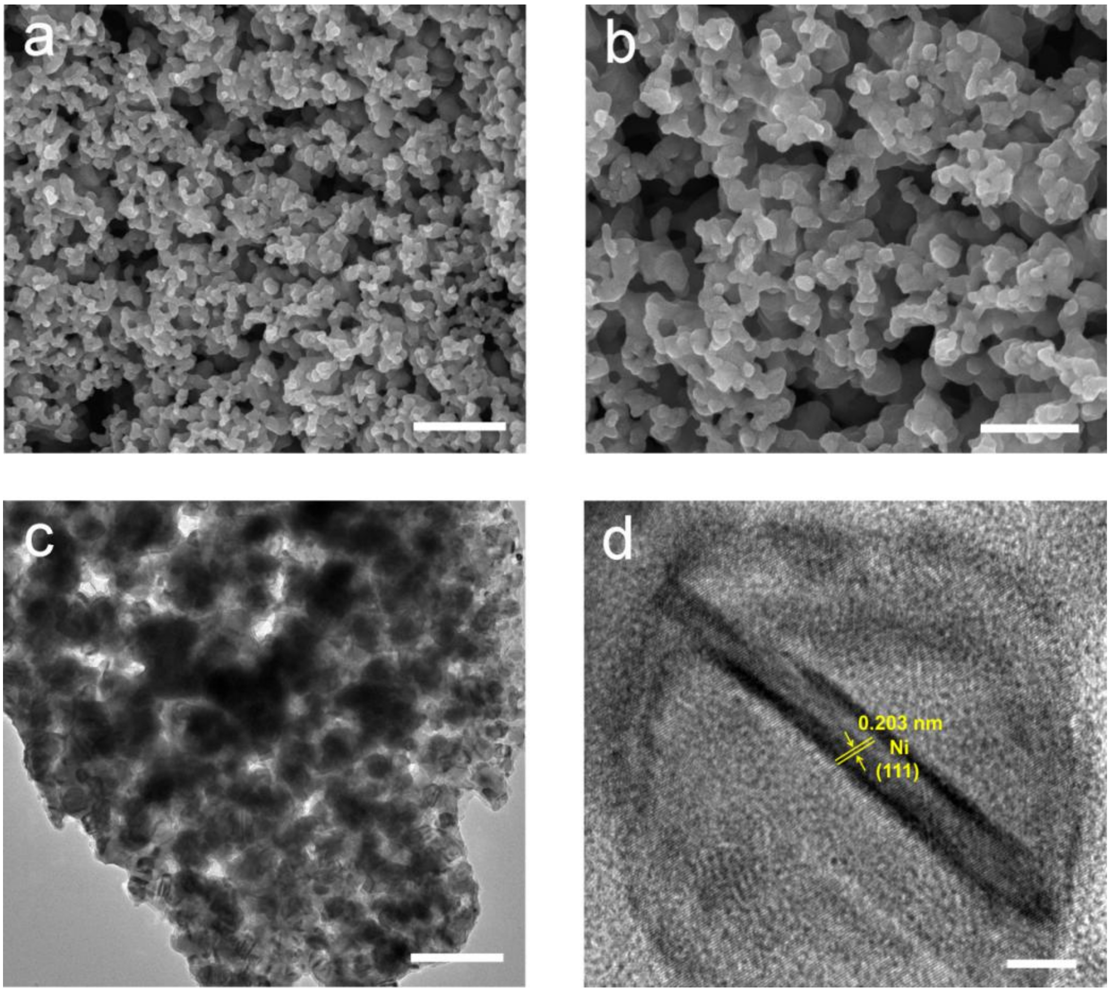

Supplementary Figure 16. Morphology and structural characterizations of Ni@GP electrode. a, b Low-magnification (scale bar: 1 μm) and high-magnification (scale bar: 500 nm) SEM images of Ni@GP electrode. **c, d** Low-magnification (scale bar: 200 nm) and high-magnification (scale bar: 5 nm) TEM images of Ni nanoparticles.

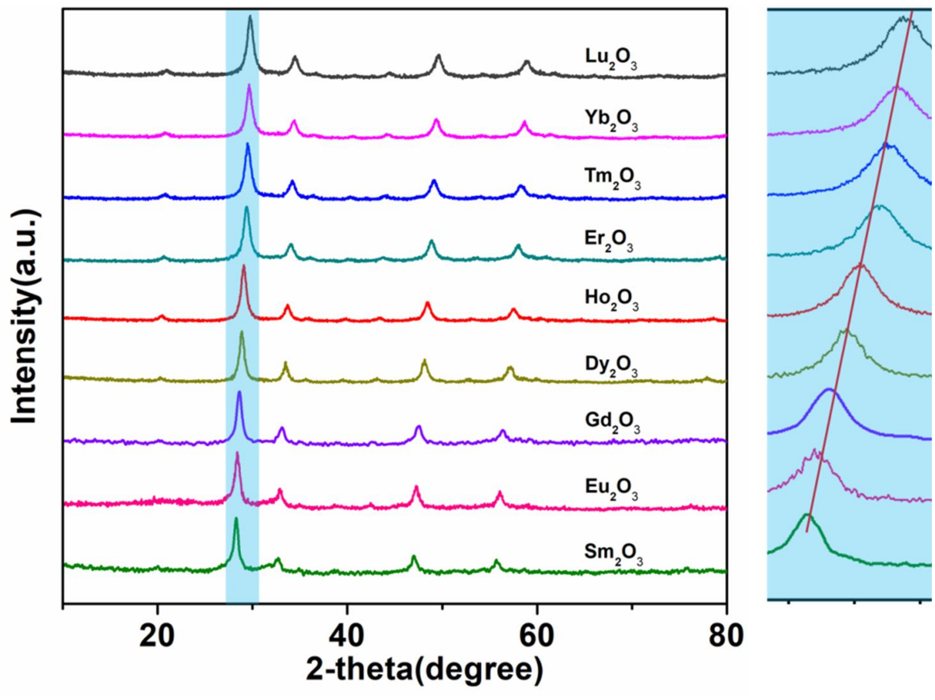

Supplementary Figure 17. Phase characterizations. XRD patterns of Ln_2O_3 powders, including Sm_2O_3, Eu_2O_3, Gd_2O_3, Dy_2O_3, Ho_2O_3, Er_2O_3, Tm_2O_3, Yb_2O_3, and Lu_2O_3.

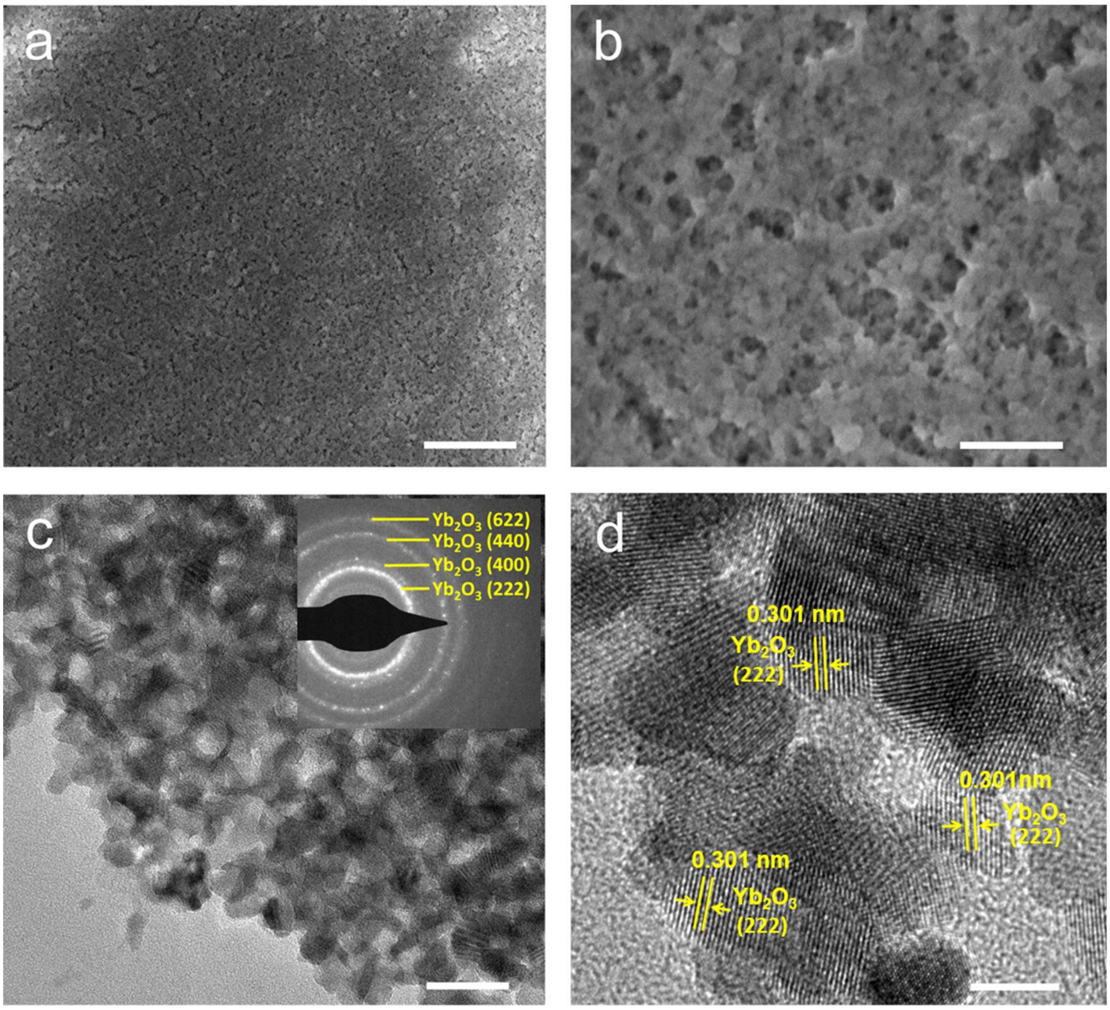

Supplementary Figure 18. Morphology and structural characterizations of Yb_2O_3@GP electrode. a, b Low-magnification (scale bar: 5 μm) and high-magnification (scale bar: 1 μm) SEM images of Yb_2O_3@GP electrode. **c, d** Low-magnification (scale bar: 20 nm; inset: SAED pattern) and high-magnification (scale bar: 5 nm) TEM images of Yb_2O_3 nanoparticles.

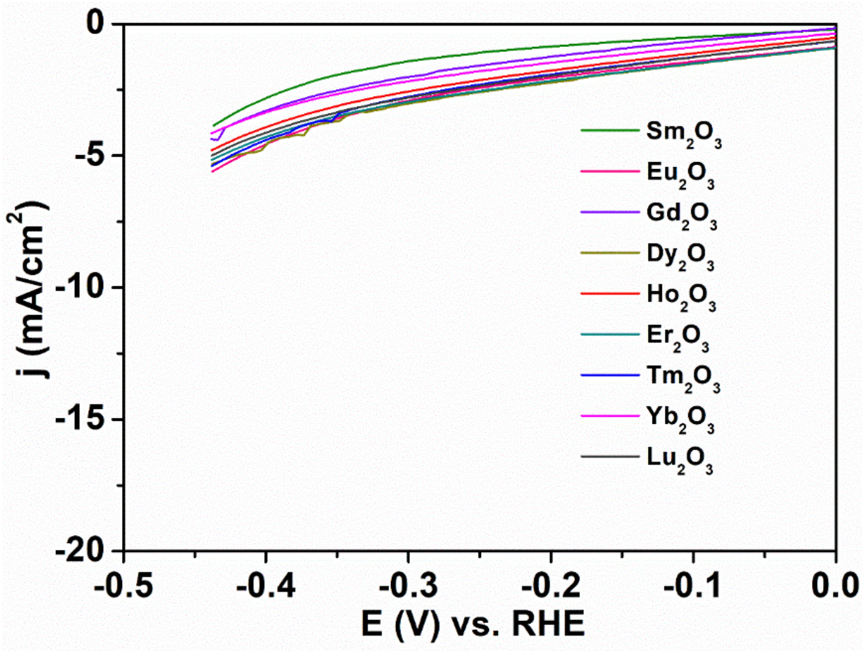

Supplementary Figure 19. Electrocatalytic HER performances of Ln_2O_3 in 1.0 M KOH electrolyte. Polarization curves of the Ln_2O_3 electrodes with mass loading of ca. 3.5 mg cm^{-2} (scan rate: 5 mV s^{-1}).

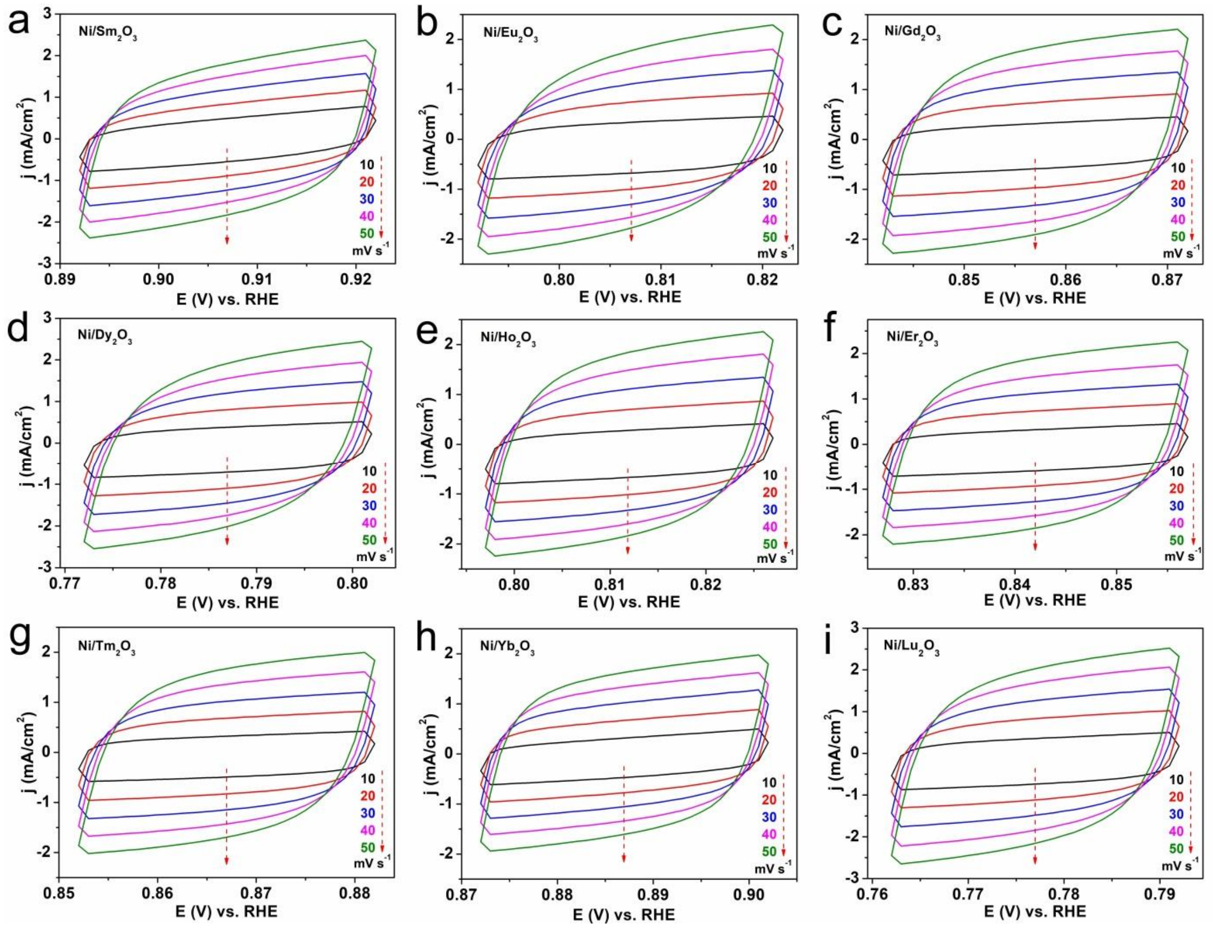

Supplementary Figure 20. Double-layer capacitance (C_{dl}) measurements in 1 M KOH. CV curves at different scan rates within the non-Faradaic potential range for **a** Ni/Sm_2O_3, **b** Ni/Eu_2O_3, **c** Ni/Gd_2O_3, **d** Ni/Dy_2O_3, **e** Ni/Ho_2O_3, **f** Ni/Er_2O_3, **g** Ni/Tm_2O_3, **h** Ni/Yb_2O_3, and **i** Ni/Lu_2O_3.

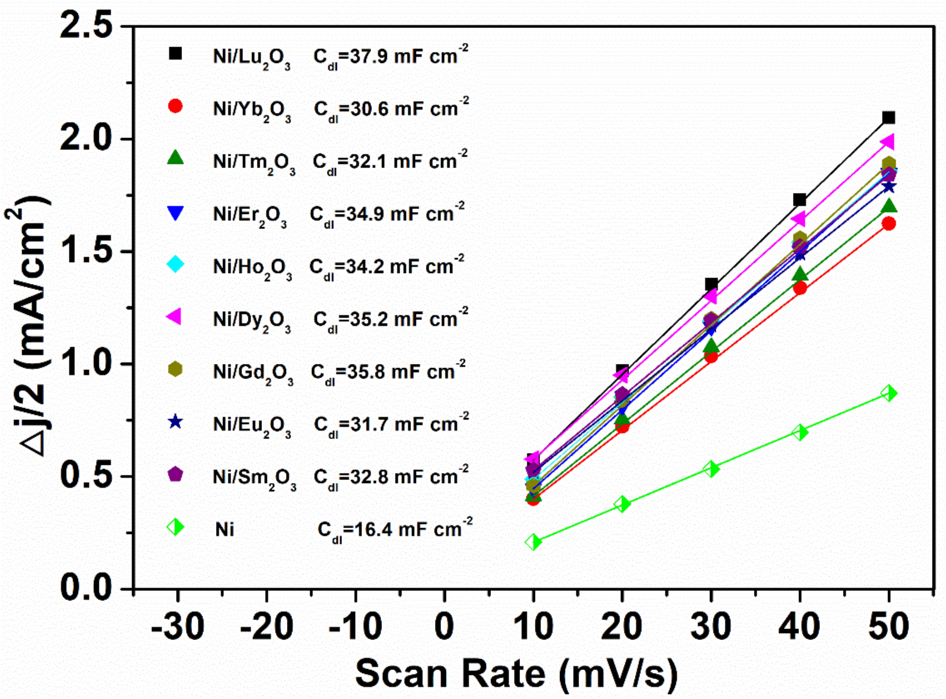

Supplementary Figure 21. Double-layer capacitance (C_{dl}). Capacitive currents on the basis of scan rate for Ni/Ln_2O_3 and Ni electrodes.

The ECSA was determined assuming a general specific C_{dl} capacitance of 40 μF cm^{-2} for all samples[5], and the ECSA is estimated by the following formula:

$$A_{ECSA}^{Samples} = \frac{\text{Specific capacitance } (\mu F\ cm^{-2})}{40\ \mu F\ cm^{-2} \text{ per } cm_{ECSA}^{2}} \tag{7}$$

$$A_{ECSA}^{Ni/Lu_2O_3} = \frac{37.9\ mF\ cm^{-2}}{40\ \mu F\ cm^{-2} \text{ per } cm_{ECSA}^{2}} = 947.5\ cm_{ECSA}^{2}$$

$$A_{ECSA}^{Ni/Yb_2O_3} = \frac{30.6\ mF\ cm^{-2}}{40\ \mu F\ cm^{-2} \text{ per } cm_{ECSA}^{2}} = 765.0\ cm_{ECSA}^{2}$$

$$A_{ECSA}^{Ni/Tm_2O_3} = \frac{32.1\ mF\ cm^{-2}}{40\ \mu F\ cm^{-2} \text{ per } cm_{ECSA}^{2}} = 802.5\ cm_{ECSA}^{2}$$

$$A_{ECSA}^{Ni/Er_2O_3} = \frac{34.9\ mF\ cm^{-2}}{40\ \mu F\ cm^{-2} \text{ per } cm_{ECSA}^{2}} = 872.5\ cm_{ECSA}^{2}$$

$$A_{ECSA}^{Ni/Ho_2O_3} = \frac{34.2\ mF\ cm^{-2}}{40\ \mu F\ cm^{-2} \text{ per } cm_{ECSA}^{2}} = 855.0\ cm_{ECSA}^{2}$$

$$A_{ECSA}^{Ni/Dy_2O_3} = \frac{35.2\ mF\ cm^{-2}}{40\ \mu F\ cm^{-2} \text{ per } cm_{ECSA}^{2}} = 880.0\ cm_{ECSA}^{2}$$

$$A_{ECSA}^{Ni/Gd_2O_3} = \frac{35.8\ mF\ cm^{-2}}{40\ \mu F\ cm^{-2} \text{ per } cm_{ECSA}^{2}} = 895.0\ cm_{ECSA}^{2}$$

$$A_{ECSA}^{Ni/Eu_2O_3} = \frac{31.7\ mF\ cm^{-2}}{40\ \mu F\ cm^{-2} \text{ per } cm_{ECSA}^{2}} = 792.5\ cm_{ECSA}^{2}$$

$$A_{ECSA}^{Ni/Sm_2O_3} = \frac{32.8\ mF\ cm^{-2}}{40\ \mu F\ cm^{-2} \text{ per } cm_{ECSA}^{2}} = 820.0\ cm_{ECSA}^{2}$$

$$A_{ECSA}^{Ni} = \frac{16.4\ mF\ cm^{-2}}{40\ \mu F\ cm^{-2} \text{ per } cm_{ECSA}^{2}} = 410.0\ cm_{ECSA}^{2}$$

The A_{ECSA} of Ni/Yb_2O_3 was calculated to be 765.0 cm^2_{ECSA} and the TOF value of Ni/Yb_2O_3 was calculated to be TOF (100 mV) = 0.362 s^{-1}.

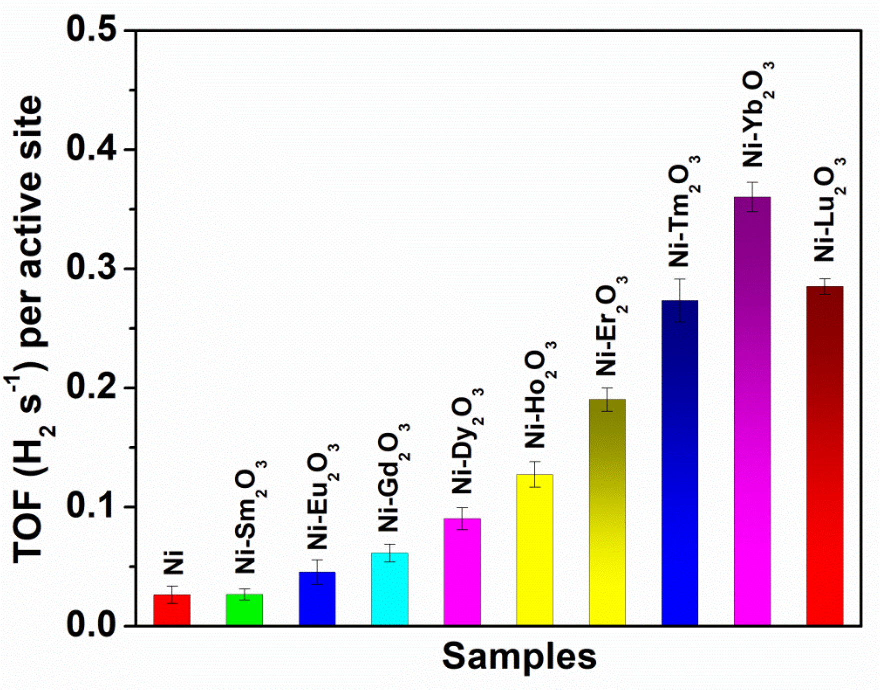

Supplementary Figure 22. Electrocatalytic HER performances of synthesized electrodes in 1.0 M KOH. Comparison of the TOF values of Ni/Ln_2O_3 and Ni at an overpotential of 100 mV. The error bars represent the standard derivation based on triplicate polarization curve measurements.

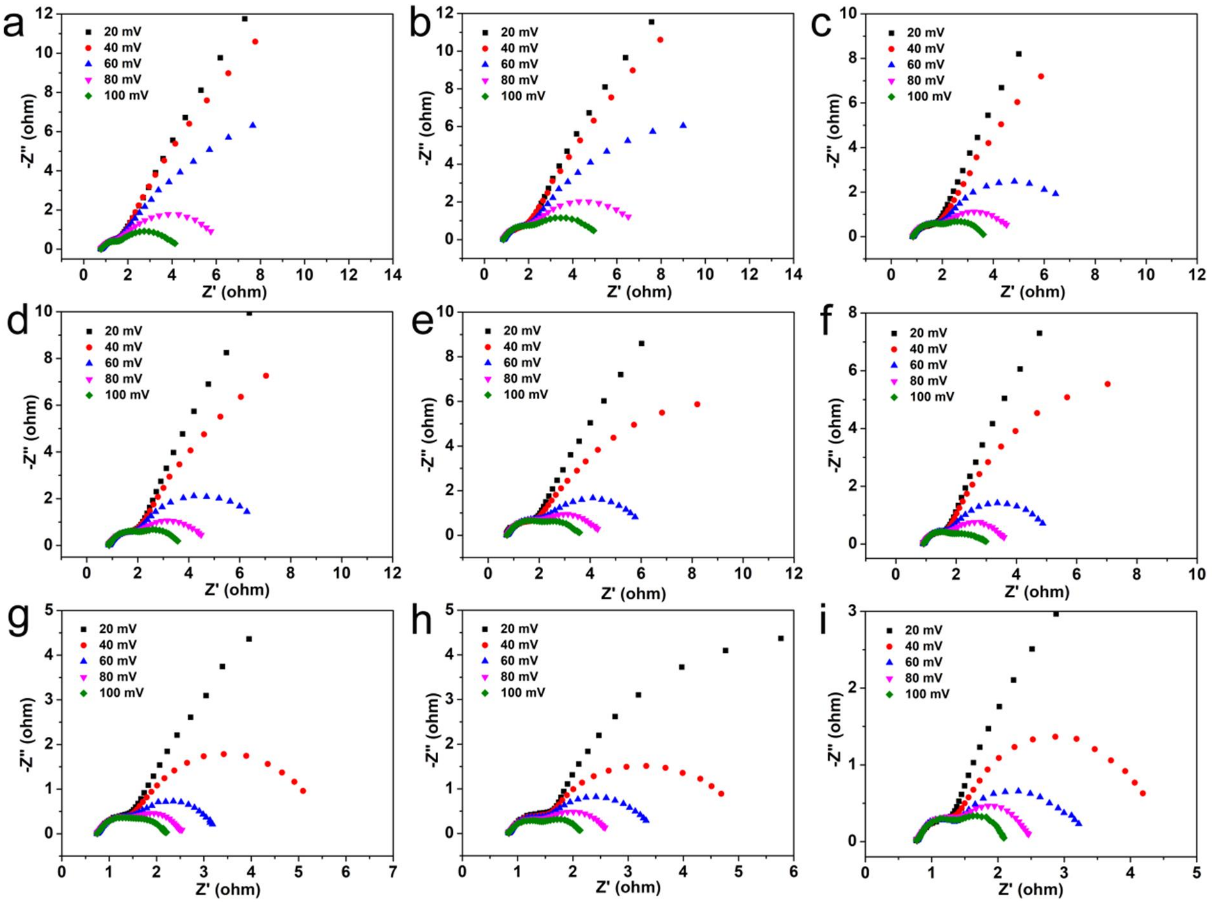

Supplementary Figure 23. Electrochemical impedance spectra of Ni/Ln_2O_3 electrodes.

Nyquist plots for **a** Ni/Sm_2O_3, **b** Ni/Eu_2O_3, **c** Ni/Gd_2O_3, **d** Ni/Dy_2O_3, **e** Ni/Ho_2O_3, **f** Ni/Er_2O_3, **g** Ni/Tm_2O_3, **h** Ni/Yb_2O_3, and **i** Ni/Lu_2O_3 collected at the overpotentials from 20 to 100 mV in 1 M KOH with amplitude of 10 mV.

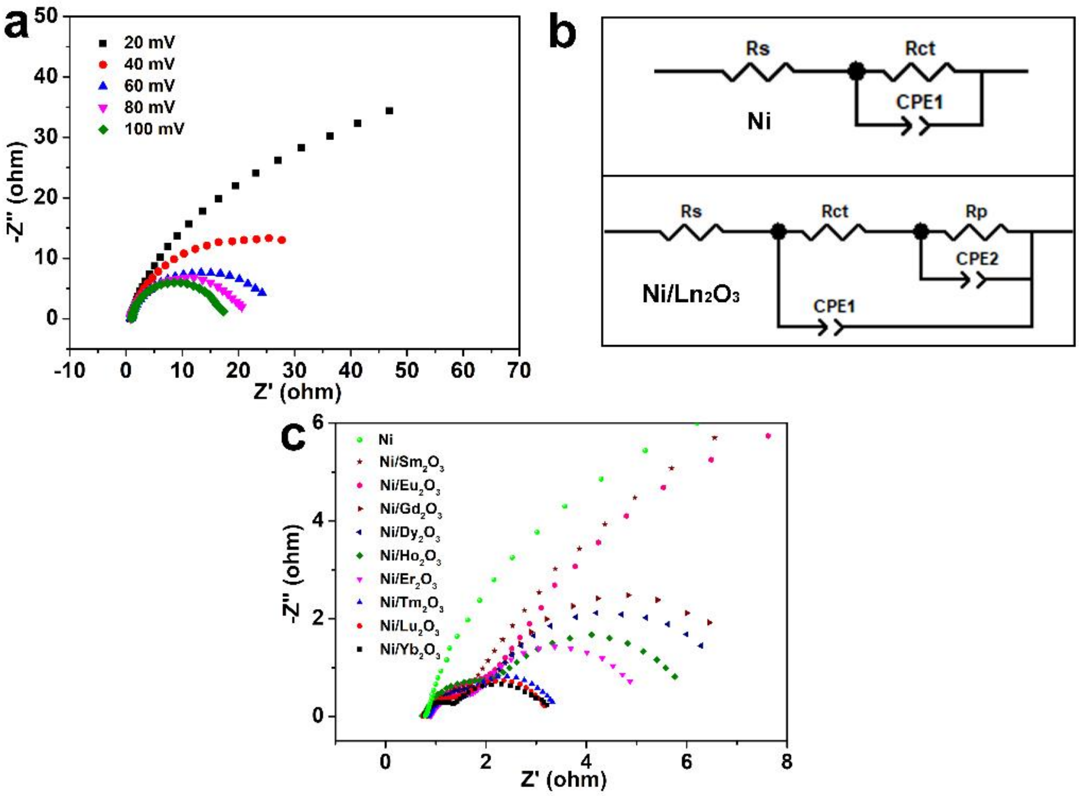

Supplementary Figure 24. Electrochemical impedance spectra and equivalent circuits of Ni and Ni/Ln_2O_3 electrodes. a Nyquist plots for Ni collected at the overpotentials from 20 to 100 mV in 1 M KOH with amplitude of 10 mV. **b** Randles and Armstrong equivalent circuits used for fitting the EIS data. **c** Nyquist plots for Ni/Ln_2O_3 collected at the overpotentials at 60 mV in 1 M KOH with amplitude of 10 mV.

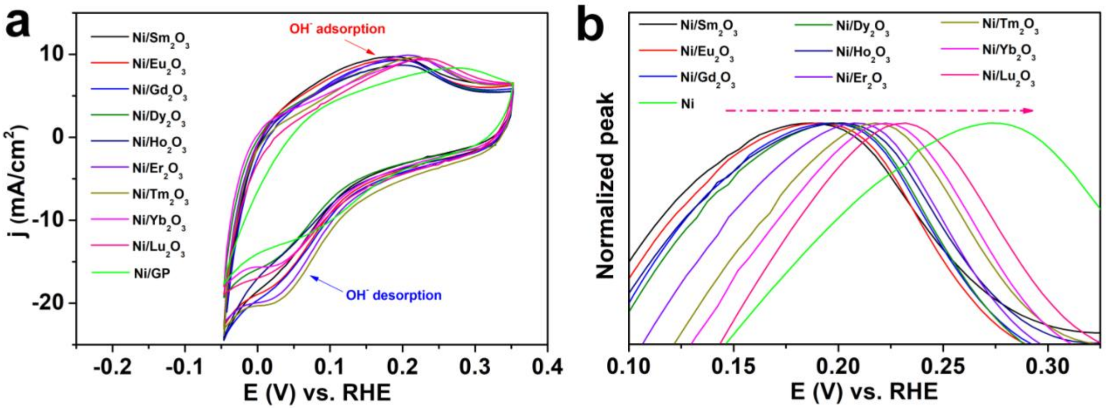

Supplementary Figure 25. Potential measurements for adsorption/desorption of OH^- on electrode surface. a Cyclic voltammograms of Ni/Ln_2O_3 and Ni electrodes in Ar-saturated 1.0 M KOH at a scan rate of 20 mV s^{-1}. **b** Normalized cyclic voltammograms of Ni/Ln_2O_3 and Ni electrodes in Ar-saturated 1.0 M KOH at a scan rate of 20 mV s^{-1}.

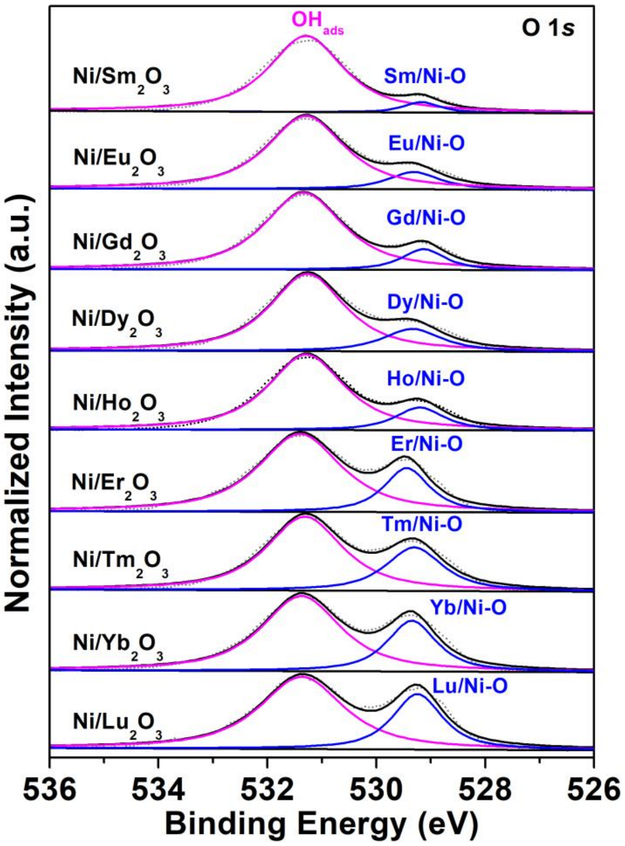

Supplementary Figure 26. XPS characterization. O 1*s* XPS spectra of Ni/Ln_2O_3 samples.

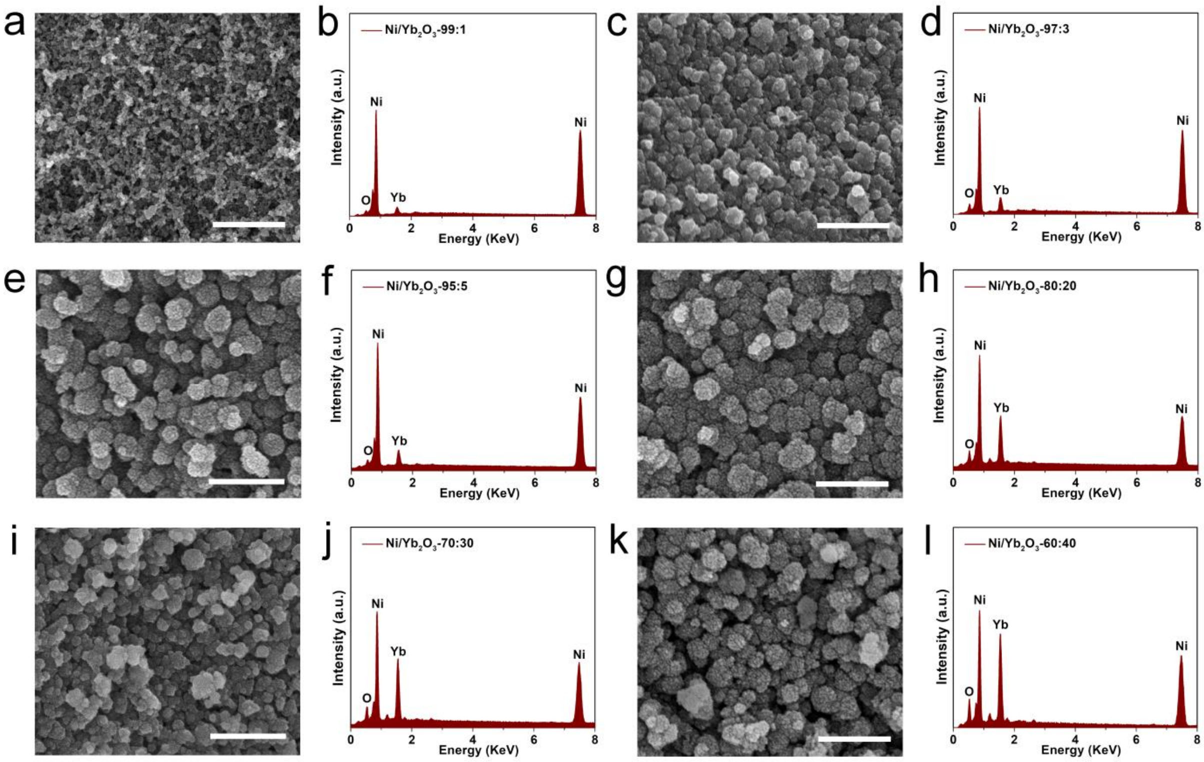

Supplementary Figure 27. Characterization of Ni/Yb_2O_3 electrodes with different Ni:Yb molar ratios. **a** SEM image and **b** EDS curve of Ni/Yb_2O_3-99:1 with the Ni:Yb molar ratio of 89.94:1.06. **c** SEM image and **d** EDS curve of Ni/Yb_2O_3-97:3 with the Ni:Yb molar ratio of 96.38:3.62. **e** SEM image and **f** EDS curve of Ni/Yb_2O_3-95:5, with the Ni:Yb molar ratio of 95.09:4.91. **g** SEM image and **h** EDS curve of Ni/Yb_2O_3-80:20 with the Ni:Yb molar ratio of 80.82:19.18. **i** SEM image and **j** EDS curve of Ni/Yb_2O_3-70:30 with the Ni:Yb molar ratio of 69.15:30.85. **k** SEM image and **l** EDS curve of Ni/Yb_2O_3-60:40 with the Ni:Yb molar ratio of 66.15:33.85. Scale bar: 500 nm.

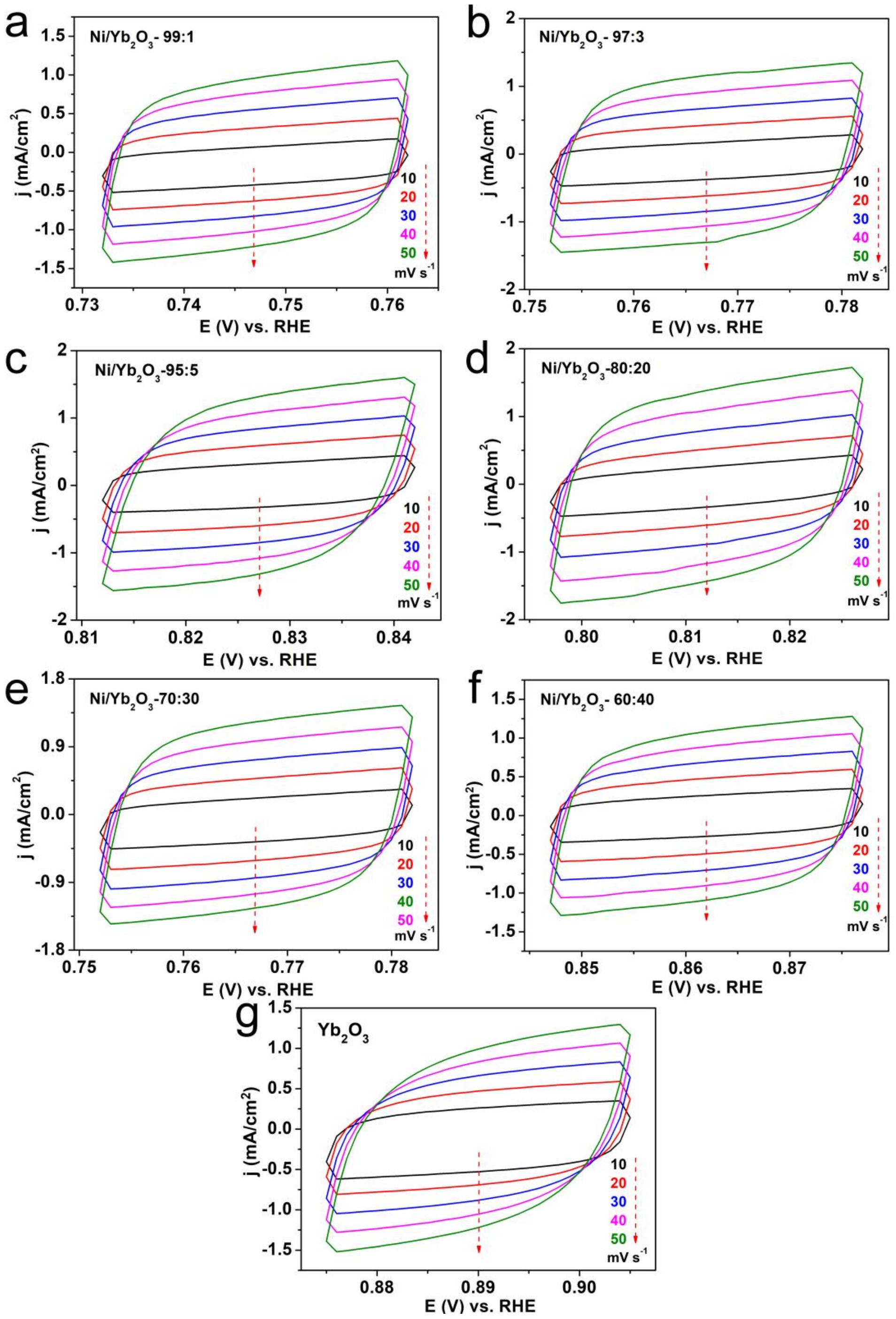

Supplementary Figure 28. Double-layer capacitance (C_{dl}) measurements in 1 M KOH.

CV curves at different scan rates within the non-Faradaic potential range for **a** Ni/Yb_2O_3-99:1, **b** Ni/Yb_2O_3-97:3, **c** Ni/Yb_2O_3-95:5, **d** Ni/Yb_2O_3-80:20, **e** Ni/Yb_2O_3-70:30, **f** Ni/Yb_2O_3-60:40, and **g** Yb_2O_3.

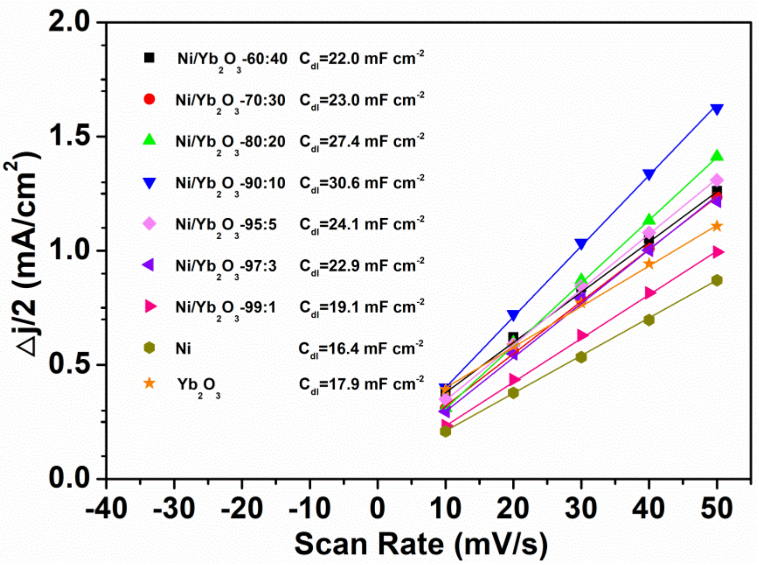

Supplementary Figure 29. Double-layer capacitance (C_{dl}). Capacitive currents on the basis of scan rate for Ni, Ni/Yb_2O_3-99:1, Ni/Yb_2O_3-97:3, Ni/Yb_2O_3-95:5, Ni/Yb_2O_3-90:10 (i.e. Ni/Yb_2O_3), Ni/Yb_2O_3-80:20, Ni/Yb_2O_3-70:30, Ni/Yb_2O_3-60:40, and Yb_2O_3 electrodes. The ECSA was determined assuming a general specific C_{dl} capacitance of 40 μF cm^{-2}.

$$A_{ECSA}^{Ni/Yb_2O_3-60:40} = \frac{22.0\ \text{mF cm}^{-2}}{40\ \mu\text{F cm}^{-2}\ \text{per cm}^2_{ECSA}} = 550.0\ \text{cm}^2_{ECSA}$$

$$A_{ECSA}^{Ni/Yb_2O_3-70:30} = \frac{23.0\ \text{mF cm}^{-2}}{40\ \mu\text{F cm}^{-2}\ \text{per cm}^2_{ECSA}} = 575.0\ \text{cm}^2_{ECSA}$$

$$A_{ECSA}^{Ni/Yb_2O_3-80:20} = \frac{27.4\ \text{mF cm}^{-2}}{40\ \mu\text{F cm}^{-2}\ \text{per cm}^2_{ECSA}} = 685.0\ \text{cm}^2_{ECSA}$$

$$A_{ECSA}^{Ni/Yb_2O_3-95:5} = \frac{24.1\ \text{mF cm}^{-2}}{40\ \mu\text{F cm}^{-2}\ \text{per cm}^2_{ECSA}} = 602.5\ \text{cm}^2_{ECSA}$$

$$A_{ECSA}^{Ni/Yb_2O_3-97:3} = \frac{22.9\ \text{mF cm}^{-2}}{40\ \mu\text{F cm}^{-2}\ \text{per cm}^2_{ECSA}} = 572.5\ \text{cm}^2_{ECSA}$$

$$A_{ECSA}^{Ni/Yb_2O_3-99:1} = \frac{19.1\ \text{mF cm}^{-2}}{40\ \mu\text{F cm}^{-2}\ \text{per cm}^2_{ECSA}} = 477.5\ \text{cm}^2_{ECSA}$$

$$A_{ECSA}^{Yb_2O_3} = \frac{17.9\ \text{mF cm}^{-2}}{40\ \mu\text{F cm}^{-2}\ \text{per cm}^2_{ECSA}} = 447.5\ \text{cm}^2_{ECSA}$$

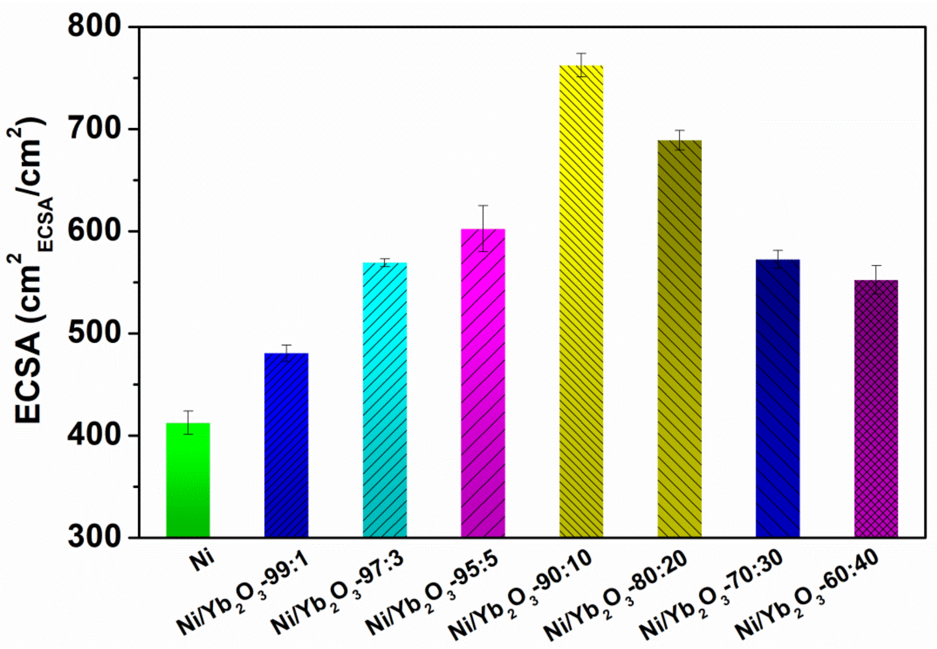

Supplementary Figure 30. Comparison of ECSAs for Ni and Ni/Yb_2O_3 electrodes with different Ni:Yb molar ratios. The error bars represent the standard derivation based on triplicate measurements.

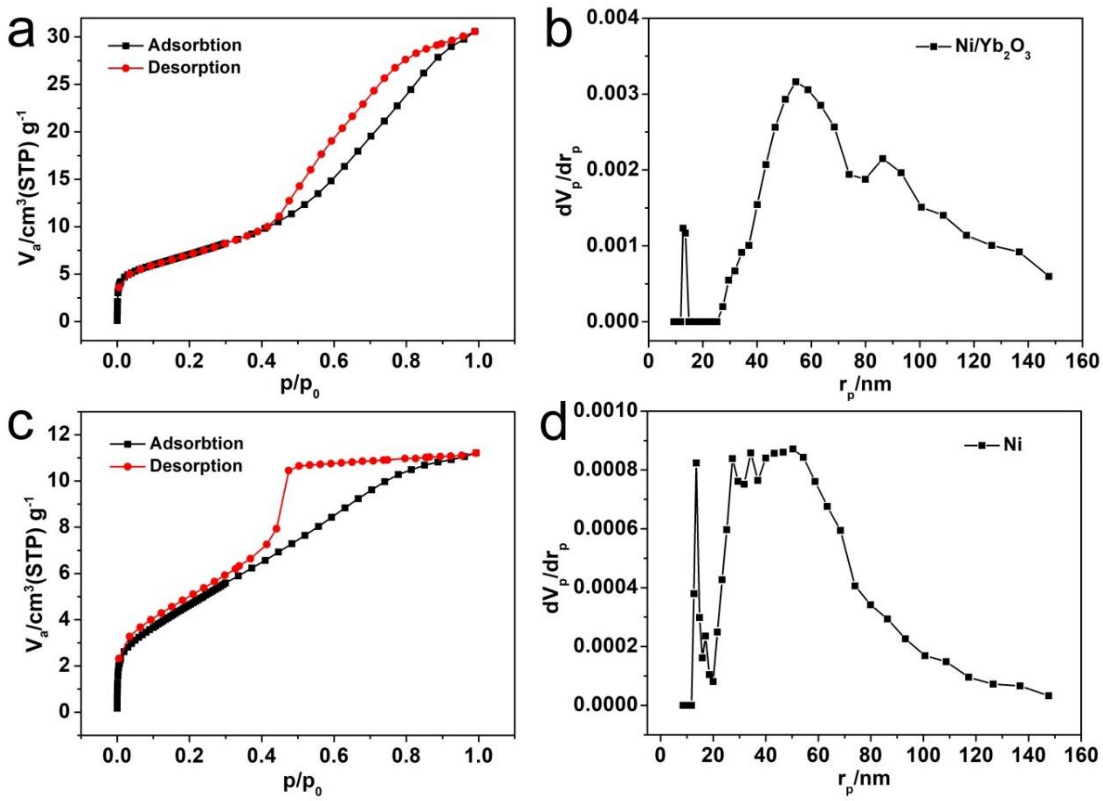

Supplementary Figure 31. Porosity of Ni/Yb_2O_3 and Ni. a Nitrogen adsorption/desorption isotherms and **b** pore size distribution for Ni/Yb_2O_3 nanoparticles. **c** Nitrogen adsorption/ desorption isotherms and **d** pore size distribution for Ni nanoparticles.

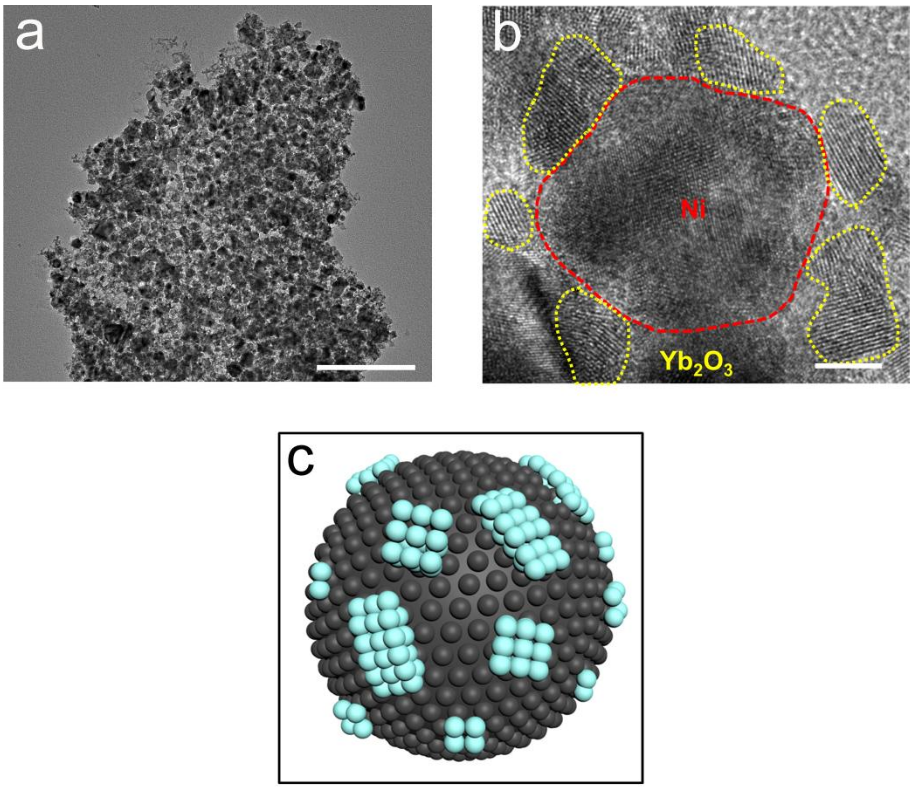

Supplementary Figure 32. Characterization of Ni/Yb_2O_3-90:10. a TEM image (scale bar: 200 nm). **b** HRTEM (scale bar: 5 nm), where yellow and red dotted lines represent Yb_2O_3 and Ni, respectively, identified by the lattice fringes. **c** Schematic illustration of Ni/Yb_2O_3-90:10 heterostructure (black and wathet spheres represent Ni and Yb_2O_3, respectively).

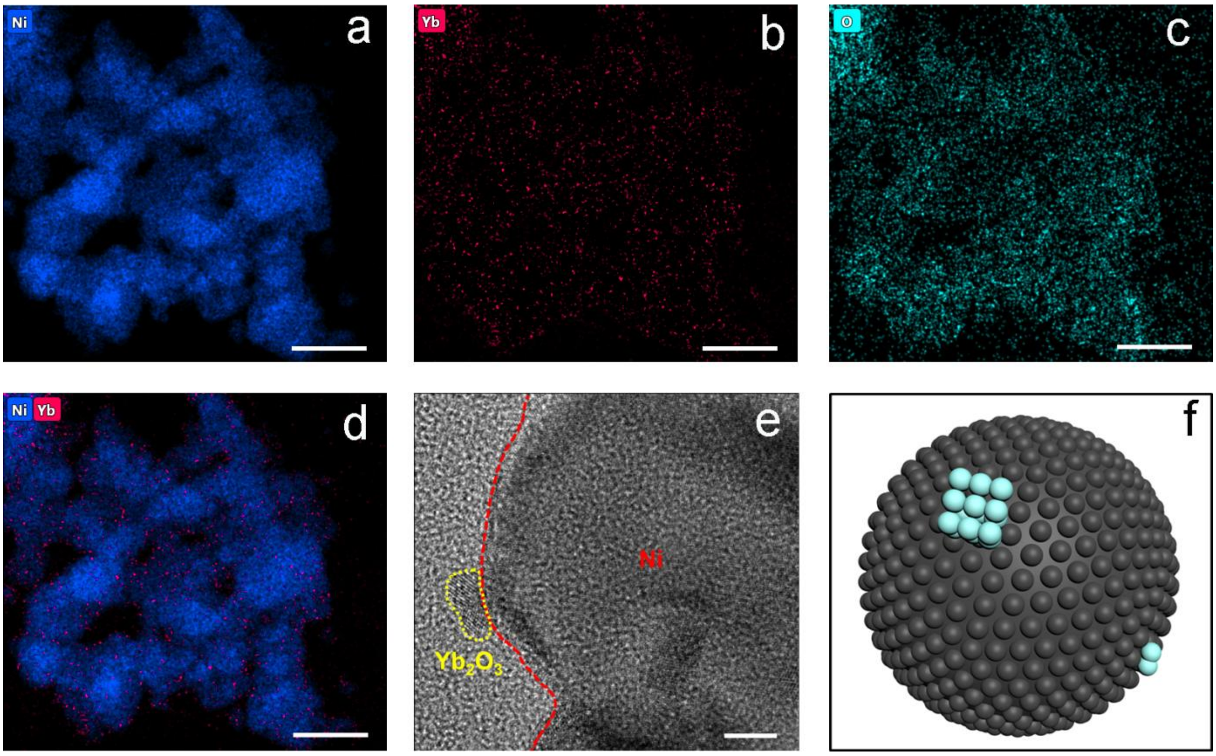

Supplementary Figure 33. Characterization of Ni/Yb_2O_3-99:1. a, b, c, d TEM-EDX elemental mappings (scale bar: 100 nm). **e** HRTEM image (scale bar: 5 nm), where yellow and red dotted lines represent Yb_2O_3 and Ni, respectively, identified by the lattice fringes. **f** Schematic illustration of Ni/Yb_2O_3-99:1 heterostructure (black and wathet spheres represent Ni and Yb_2O_3, respectively).

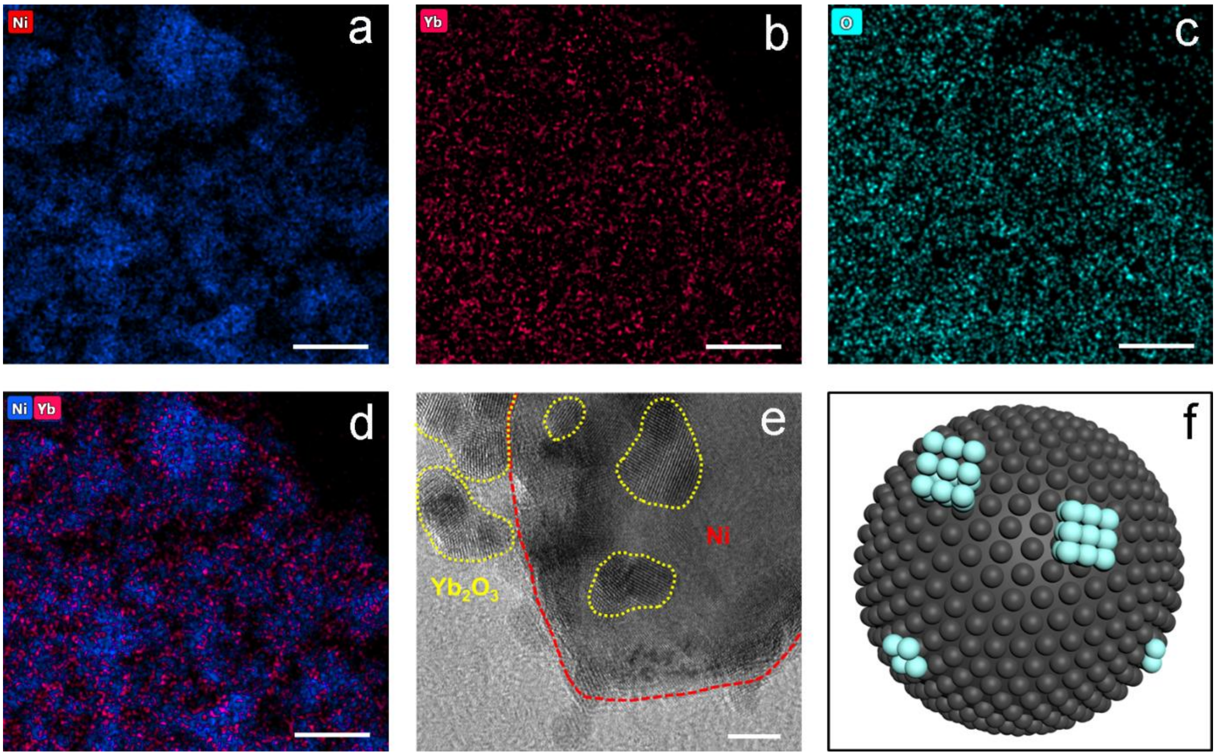

Supplementary Figure 34. Characterization of Ni/Yb_2O_3-97:3. a, b, c, d TEM-EDX elemental mappings (scale bar: 100 nm). **e** HRTEM image (scale bar: 5 nm), where yellow and red dotted lines represent Yb_2O_3 and Ni, respectively, identified by the lattice fringes. **f** Schematic illustration of Ni/Yb_2O_3-97:3 heterostructure (black and wathet spheres represent Ni and Yb_2O_3, respectively).

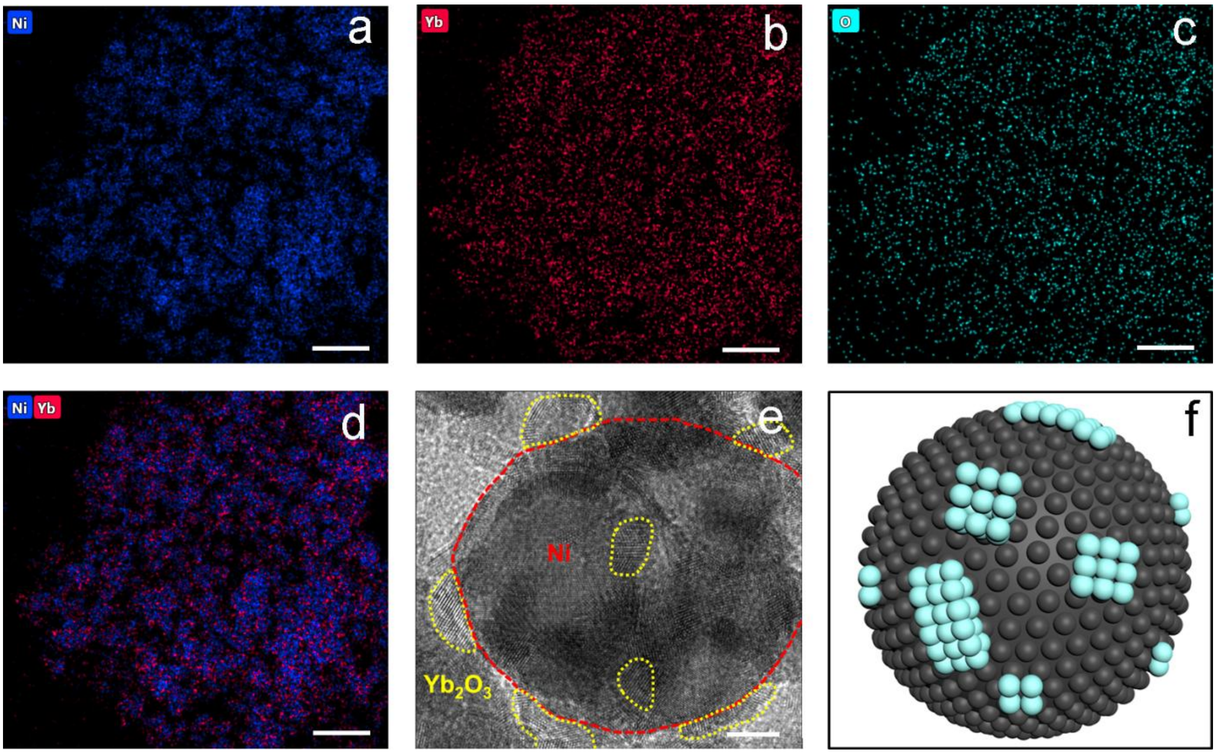

Supplementary Figure 35. Characterization of Ni/Yb_2O_3-95:5. a, b, c, d TEM-EDX elemental mappings (scale bar: 100 nm). **e** HRTEM image (scale bar: 5 nm), yellow and red dotted lines represent Yb_2O_3 and Ni, respectively, identified by the lattice fringes. **f** Schematic illustration of Ni/Yb_2O_3-95:5 heterostructure (black and wathet spheres represent Ni and Yb_2O_3, respectively).

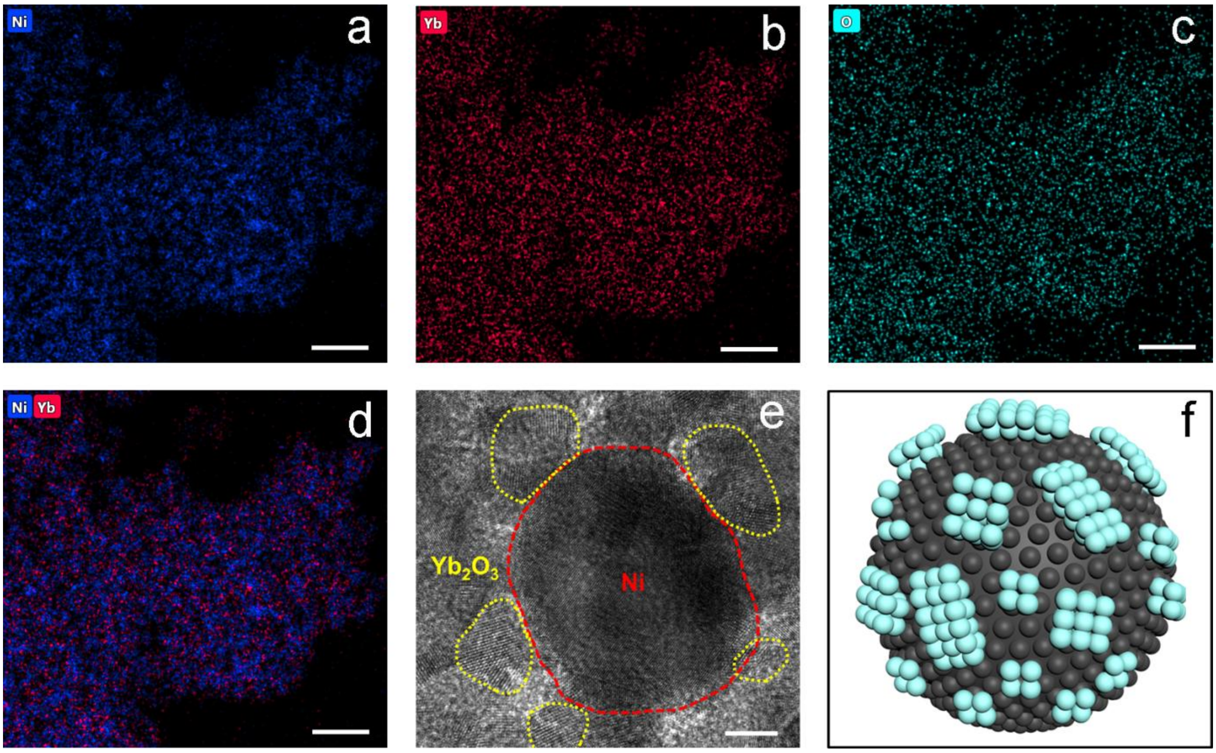

Supplementary Figure 36. Characterization of Ni/Yb_2O_3-80:20. a, b, c, d TEM-EDX elemental mappings (scale bar: 100 nm). **e** HRTEM image (scale bar: 5 nm), where yellow and red dotted lines represent Yb_2O_3 and Ni, respectively, identified by the lattice fringes. **f** Schematic illustration of Ni/Yb_2O_3-80:20 heterostructure (black and wathet spheres represent Ni and Yb_2O_3, respectively).

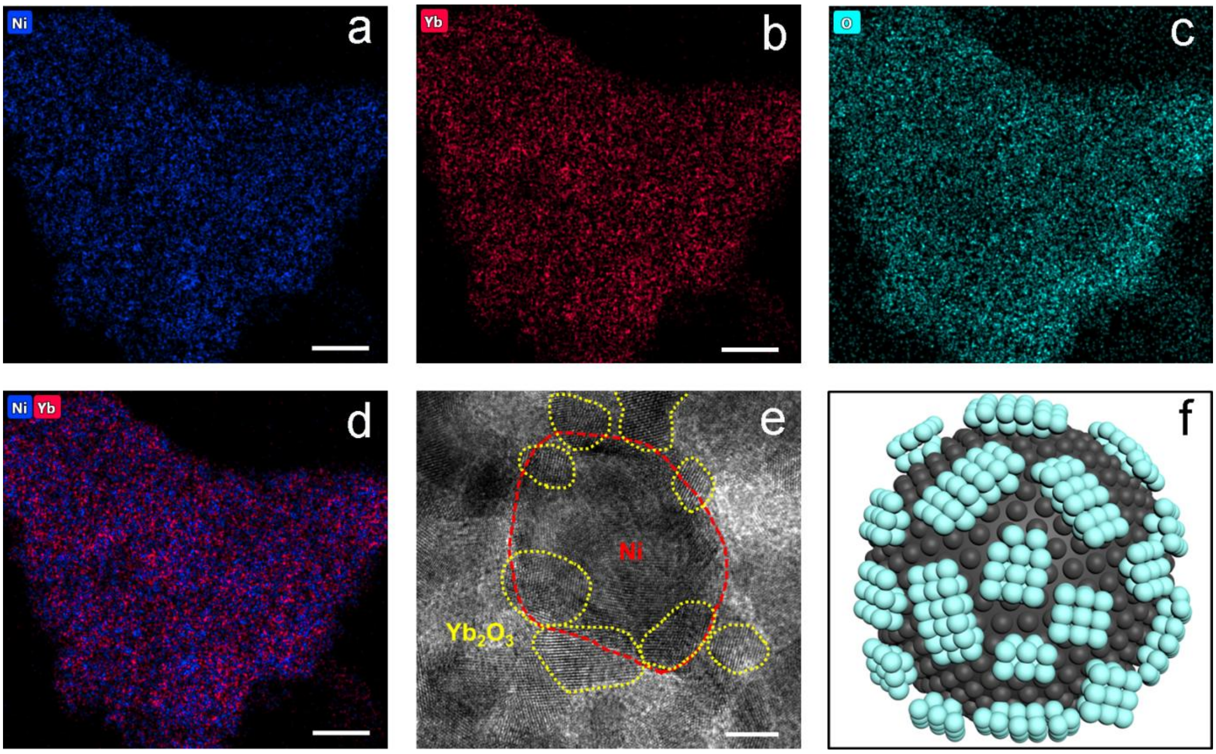

Supplementary Figure 37. Characterization of Ni/Yb_2O_3-70:30. a, b, c, d TEM-EDX elemental mappings (scale bar: 100 nm). **e** HRTEM image (scale bar: 5 nm), where yellow and red dotted lines represent Yb_2O_3 and Ni nanoparticles, respectively, identified by the lattice fringes. **f** Schematic illustration of Ni/Yb_2O_3-70:30 heterostructure (black and wathet spheres represent Ni and Yb_2O_3, respectively).

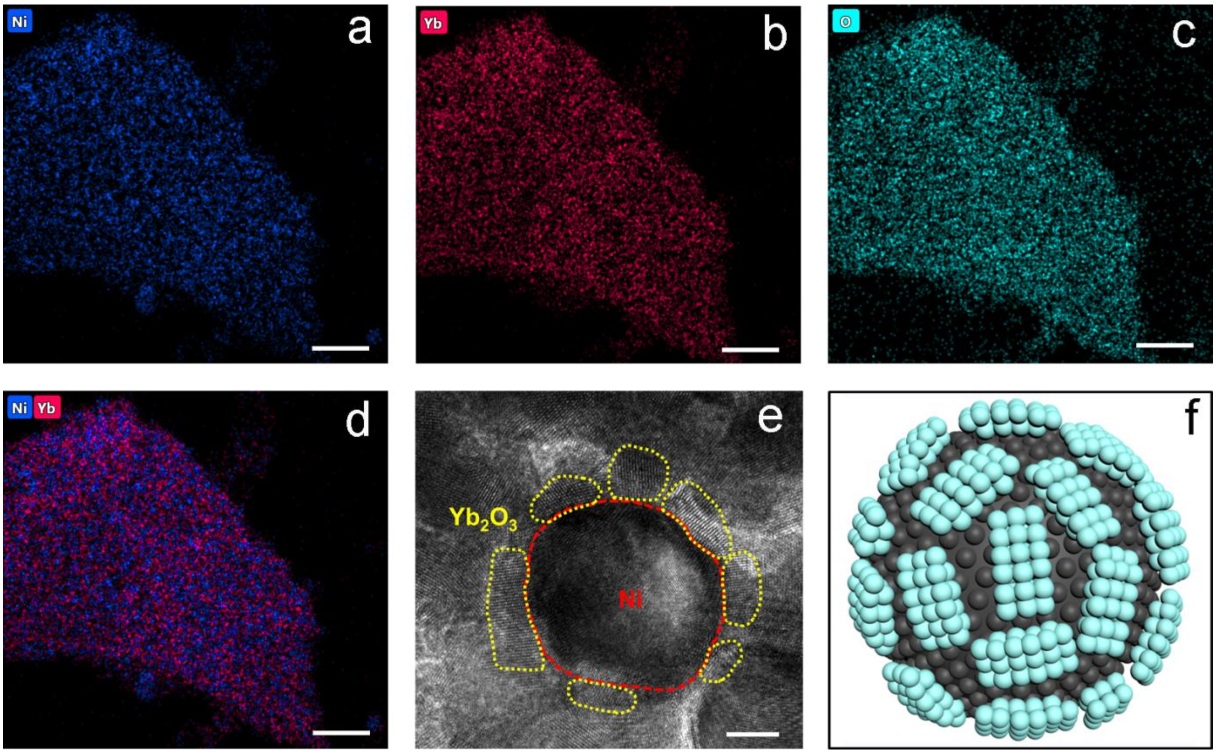

Supplementary Figure 38. Characterization of Ni/Yb$_2$O$_3$-60:40. a, b, c, d TEM-EDX elemental mappings (scale bar: 100 nm). **e** HRTEM image (scale bar: 5 nm), where yellow and red dotted lines represent Yb$_2$O$_3$ and Ni, respectively, identified by the lattice fringes. **f** Schematic illustration of Ni/Yb$_2$O$_3$-60:40 heterostructure (black and wathet spheres represent Ni and Yb$_2$O$_3$, respectively).

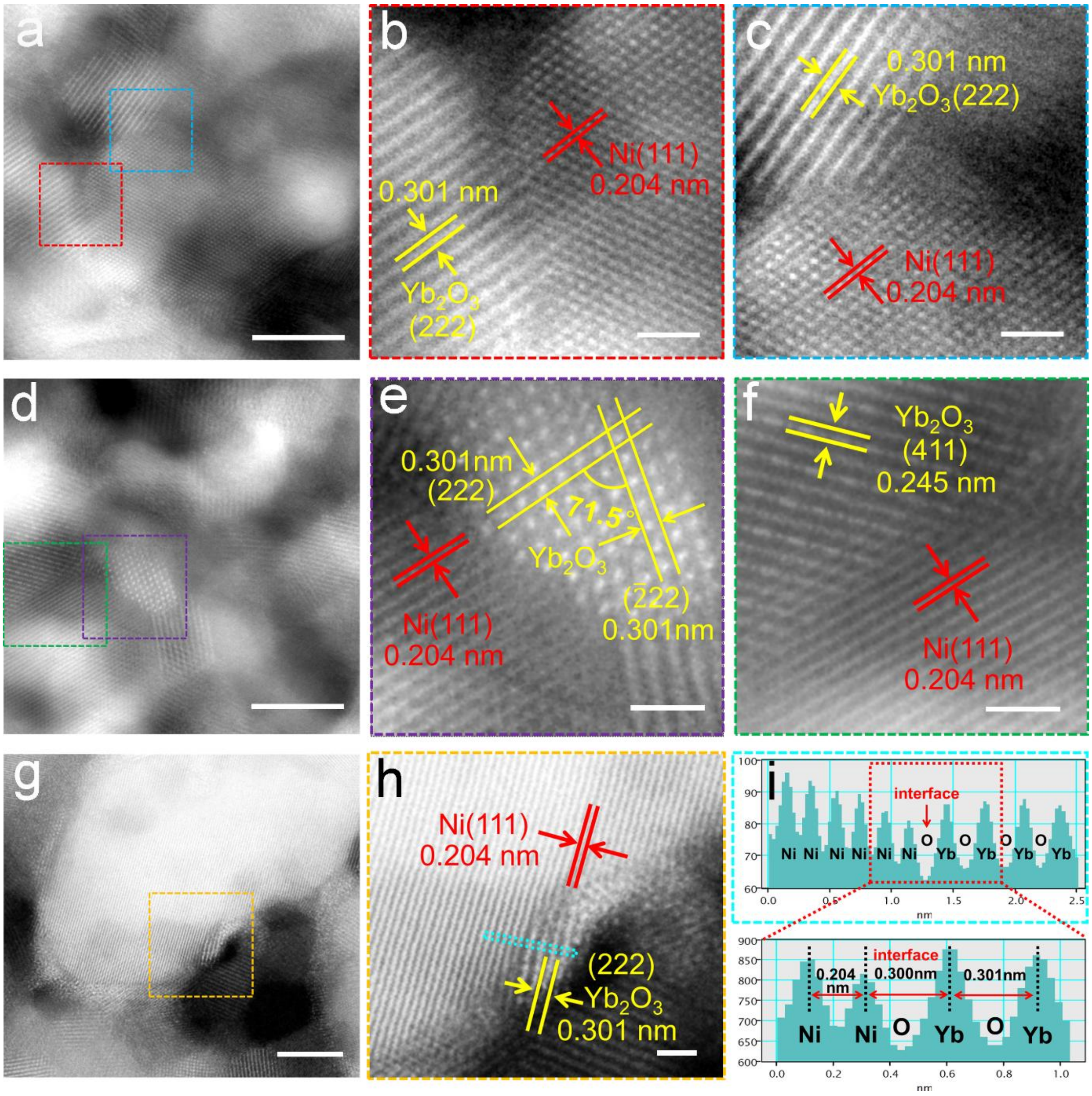

Supplementary Figure 39. Characterization of Ni/Yb_2O_3. a, d, g TEM images (scale bar: 5 nm). **b, c, e, f, h** HRTEM images (scale bar: 1 nm). **i** Line intensity profile for Ni and Yb_2O_3 indicated by the blue lines in HRTEM image **h**.

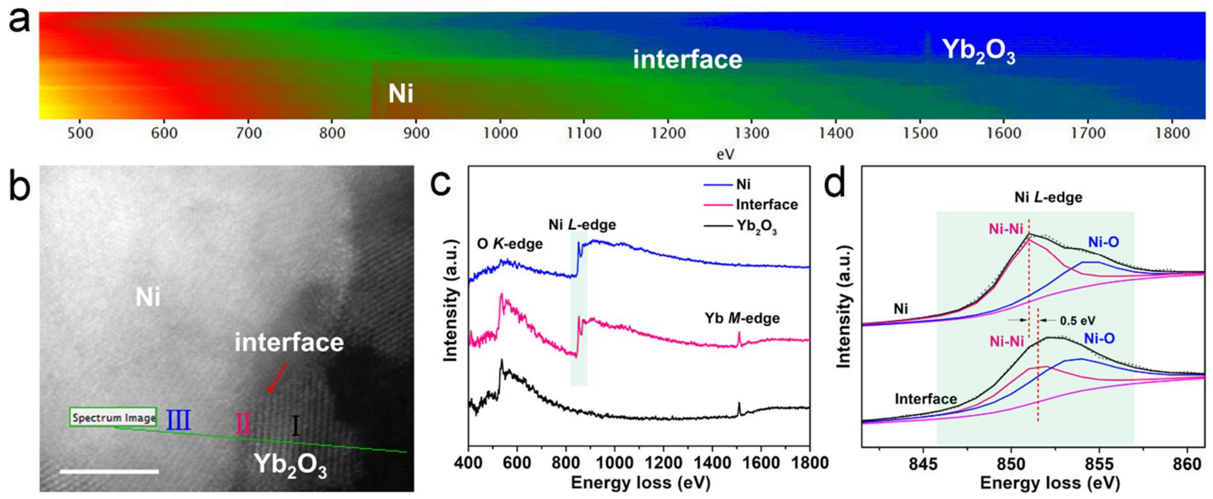

Supplementary Figure 40. Characterization of Ni/Yb_2O_3. a Line-scan electron energy loss spectroscopy (EELS). **b** HRTEM image of Ni/Yb_2O_3 recorded for line-scan EELS spectrum (scale bar: 5 nm). **c** EELS spectra collected on Yb_2O_3 phase (I), interface (II) and Ni phase (III) of Ni/Yb_2O_3 as marked in HRTEM image **b**. **d** Magnified Ni L-edge EELS plots.

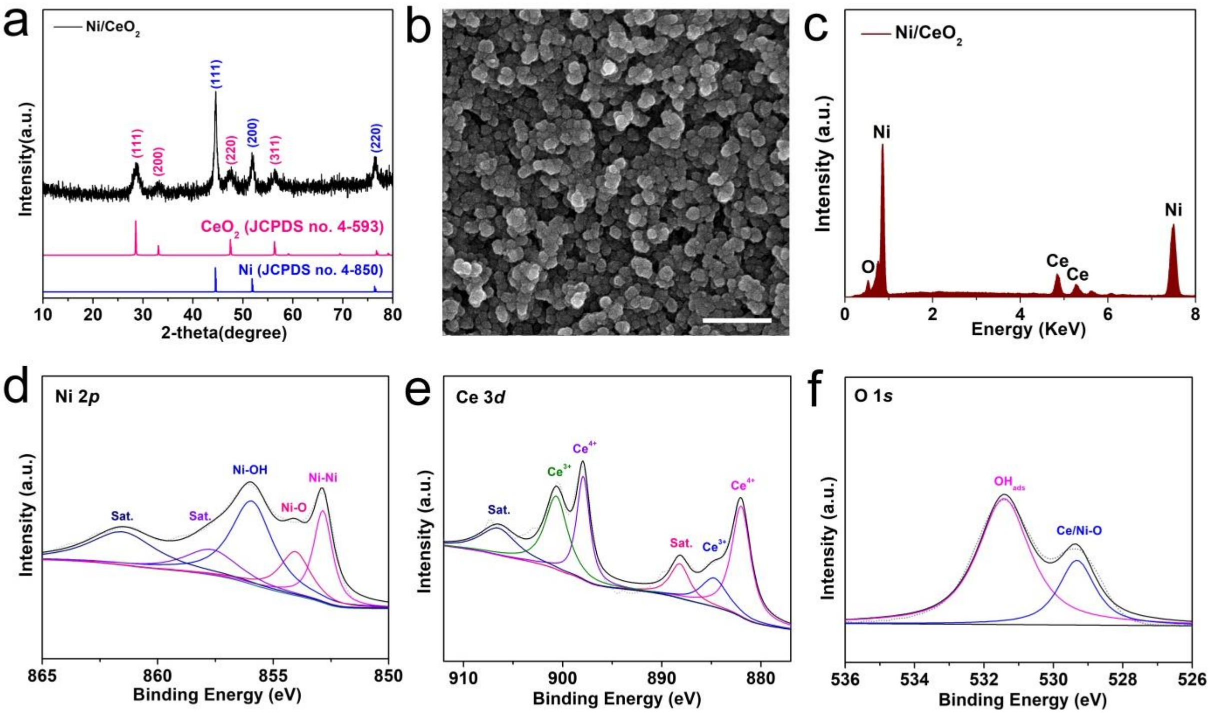

Supplementary Figure 41. Characterization of Ni/CeO$_2$. a XRD pattern. **b** SEM image (scale bar: 500 nm). **c** EDS spectrum. **d** Ni 2*p* XPS spectrum. **e** Ce 3*d* XPS spectrum. **f** O 1*s* XPS spectrum. The Ni:Ce molar ratio is 89.83:10.17.

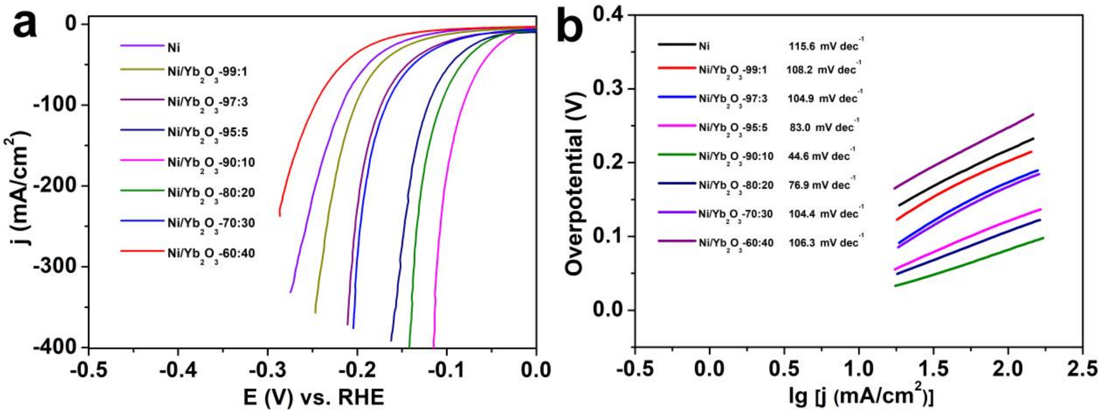

Supplementary Figure 42. Electrocatalytic HER activity of Ni and Ni/Yb_2O_3 electrodes with different Ni:Yb molar ratios in 1.0 M KOH electrolyte. a Polarization curves. **b** Tafel plots derived from the polarization curves.

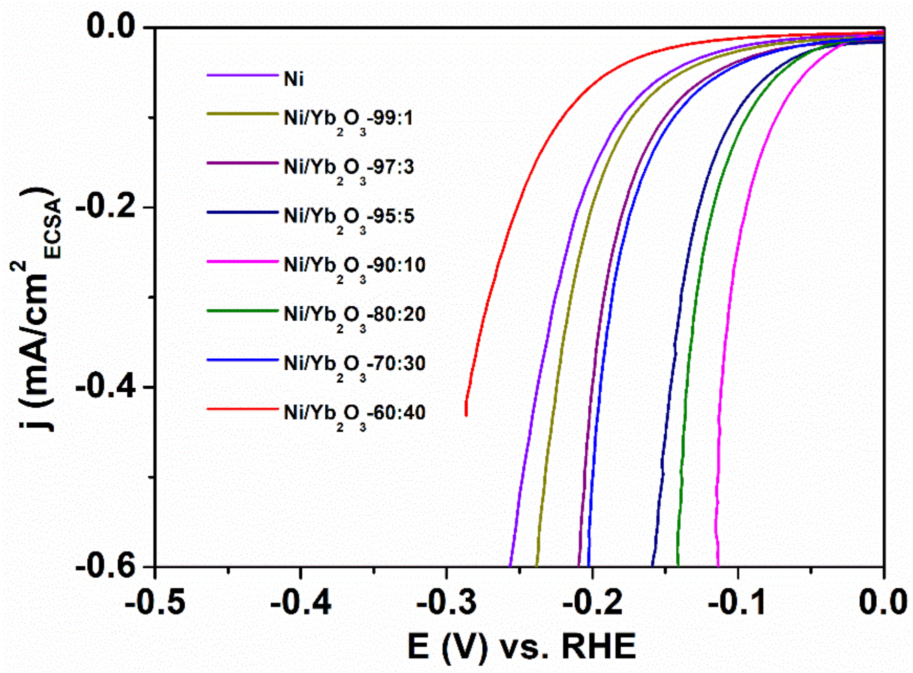

Supplementary Figure 43. Electrocatalytic HER activity of synthesized electrodes in 1.0 M KOH electrolyte. ECSA-normalized HER polarization curves of Ni and Ni/Yb_2O_3 electrodes with different Ni:Yb molar ratios (99:1, 97:3, 95:5, 90:10, 80:20, 70:30 and 60:40).

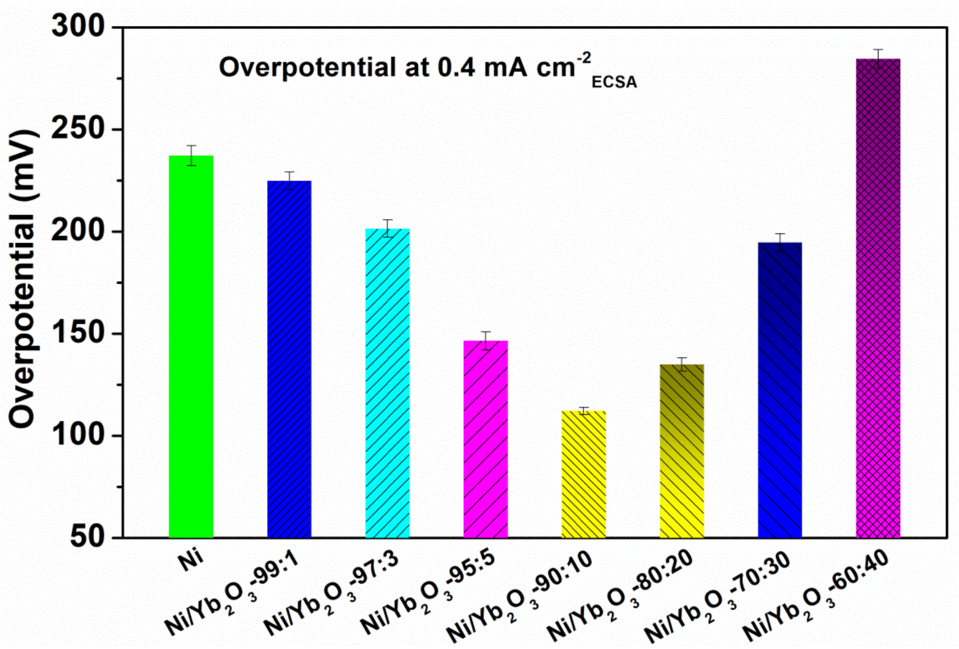

Supplementary Figure 44. Comparison of the ECSA-normalized specific activity. The overpotentials of Ni and Ni/Yb_2O_3 electrodes with different Ni:Yb molar ratios at 0.4 mA cm^{-2}_{ECSA}. The error bars represent the standard derivation based on triplicate polarization curve measurements.

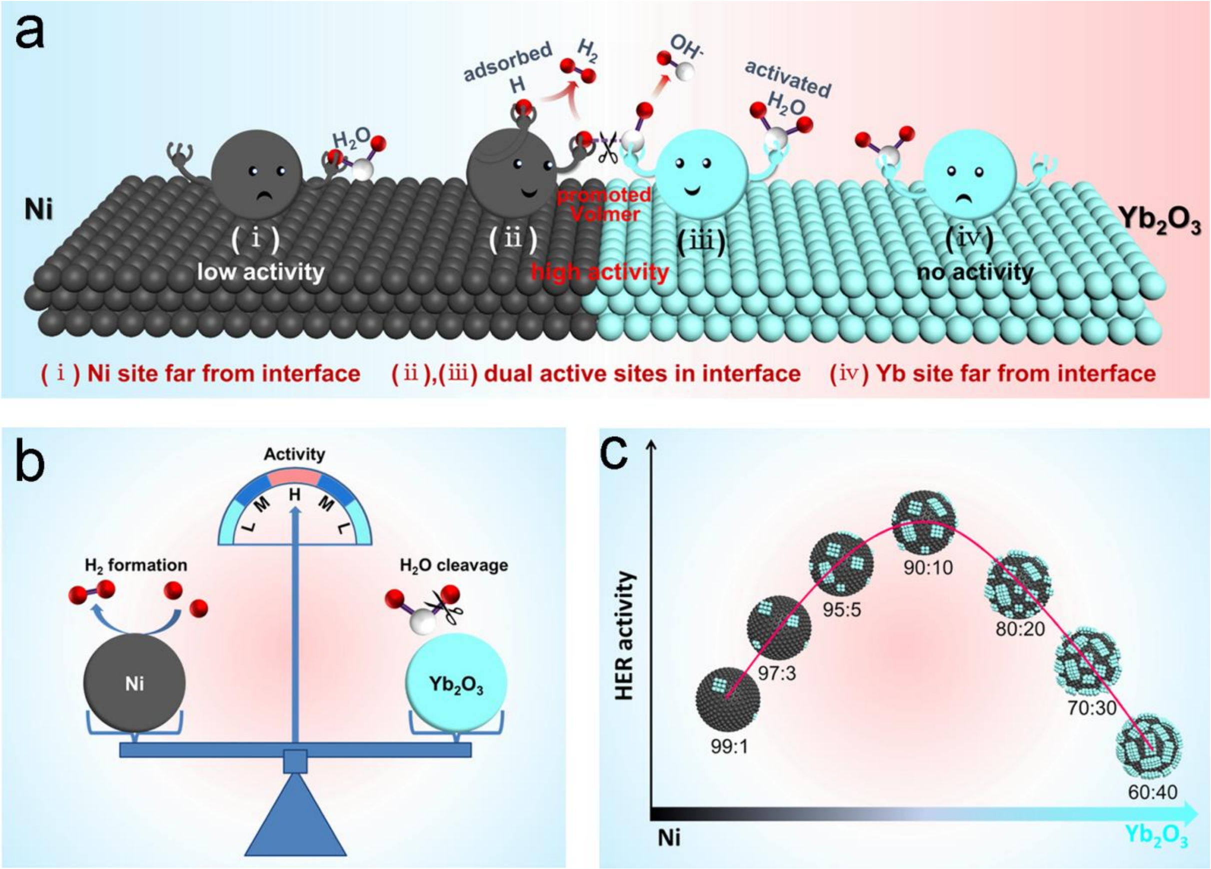

Supplementary Figure 45. Catalytic mechanism. a Schematic illustration of alkaline HER on the heterosurface of Ni/Yb_2O_3. **b** Schematic illustration for the balance of H_2O dissociation step and H_2 formation step obtained by regulating the Ni/Yb ratios. **c** Dependence of alkaline HER activity on the compositions of Ni/Yb_2O_3 hybrids.

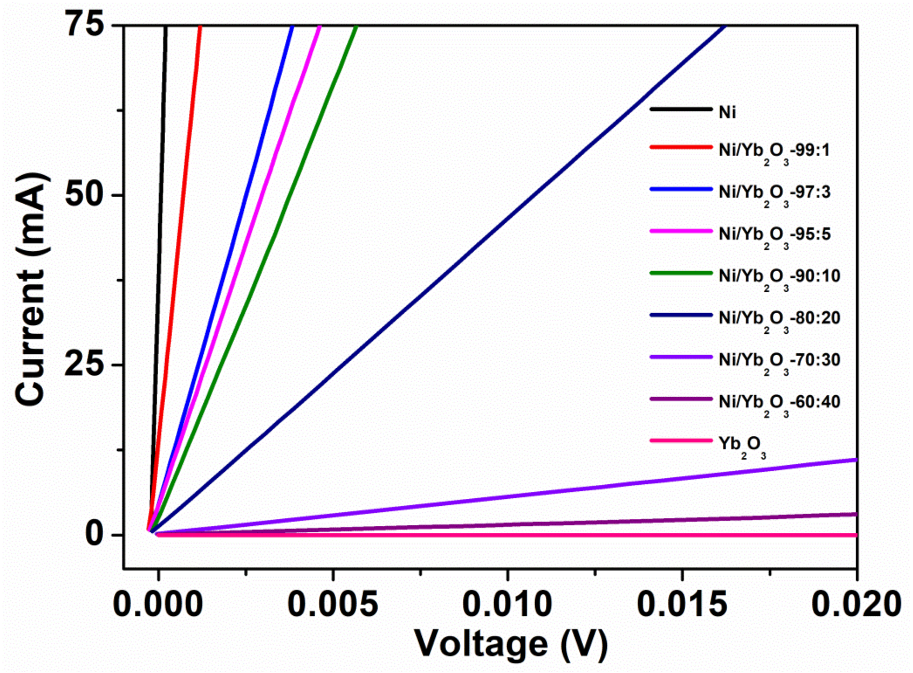

Supplementary Figure 46. Electrical conductivity measurements. The resistance values (R) of the samples were calculated by voltmeter-ammeter method[1].The electrical conductivity was determined to be 1.65×10^3, 3.61×10^2, 1.22×10^2, 1.00×10^2, 8.55×10, 2.88, 3.52, 9.71×10^{-1} and 1.46×10^{-6} S m^{-1} for Ni, Ni/Yb_2O_3-99:1, Ni/Yb_2O_3-97:3, Ni/Yb_2O_3-95:5, Ni/Yb_2O_3-90:10, Ni/Yb_2O_3-80:20, Ni/Yb_2O_3-70:30, Ni/Yb_2O_3-60:40 and Yb_2O_3, respectively. The electrical conductivity of Ni/Yb_2O_3 hybrids decreases significantly with the increase of Yb_2O_3 doping amount due to the very low electrical conductivity of Yb_2O_3.

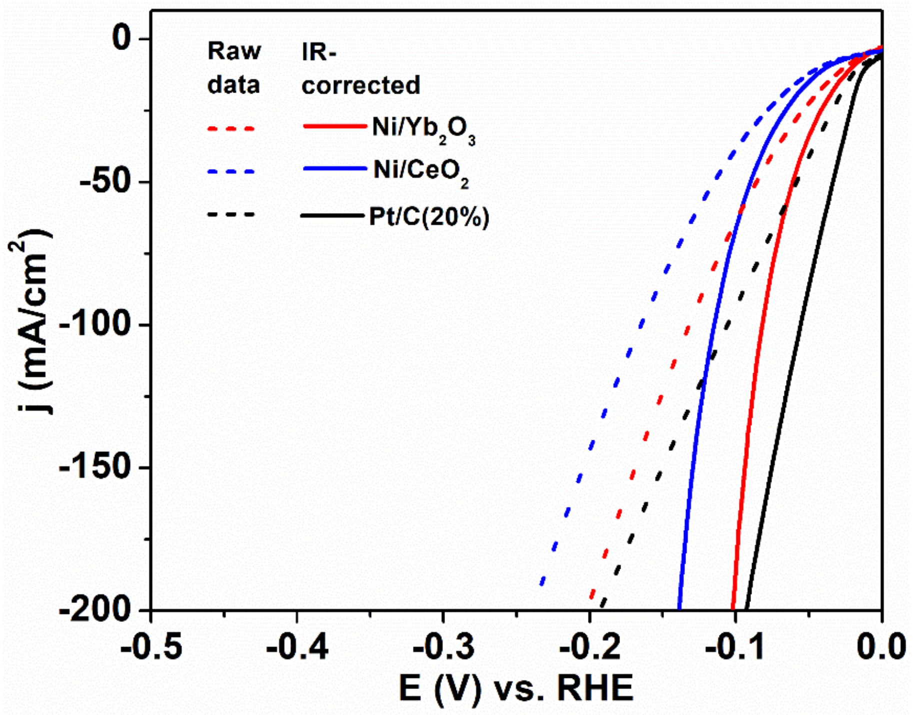

Supplementary Figure 47. Electrocatalytic HER activity of synthesized electrodes.

Original and iR-corrected HER polarization curves of Ni/Yb_2O_3, Ni/CeO_2 and Pt/C(20%) electrodes in 1 M KOH.

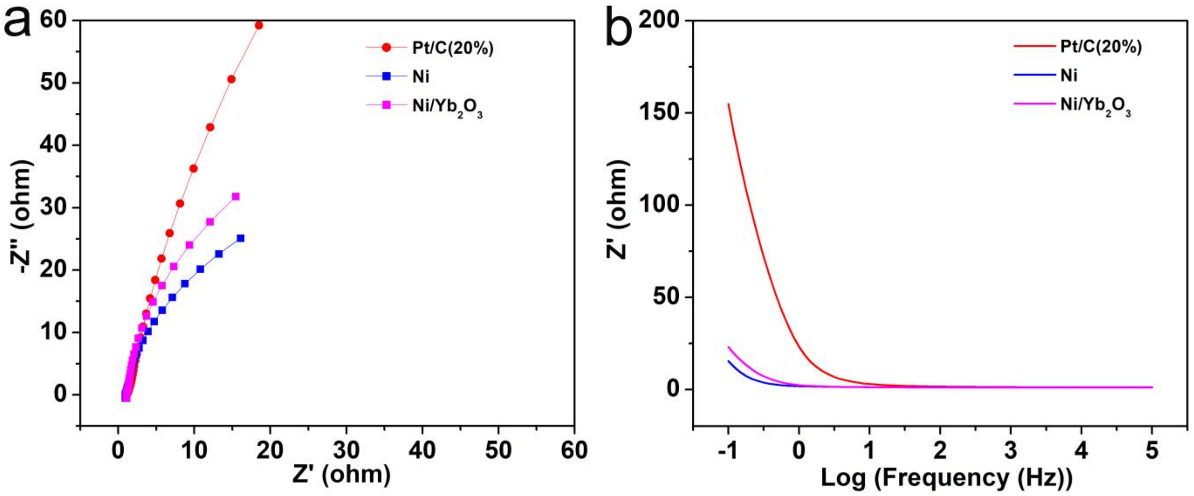

Supplementary Figure 48. Electrochemical impedance spectra of Ni/Yb_2O_3, Ni and Pt/C(20%) electrodes. a Nyquist plots collected at open circuit potential in 1 M KOH with amplitude of 10 mV. **b** The corresponding Bode plots. Electrochemical impedance spectra suggest the superior electrical conductivity of the self-supported Ni/Yb_2O_3 and Ni electrodes compared with the Pt/C(20%) electrode due to their lower interface resistance between the in-situ loaded Ni/Yb_2O_3 or Ni and the substrate.

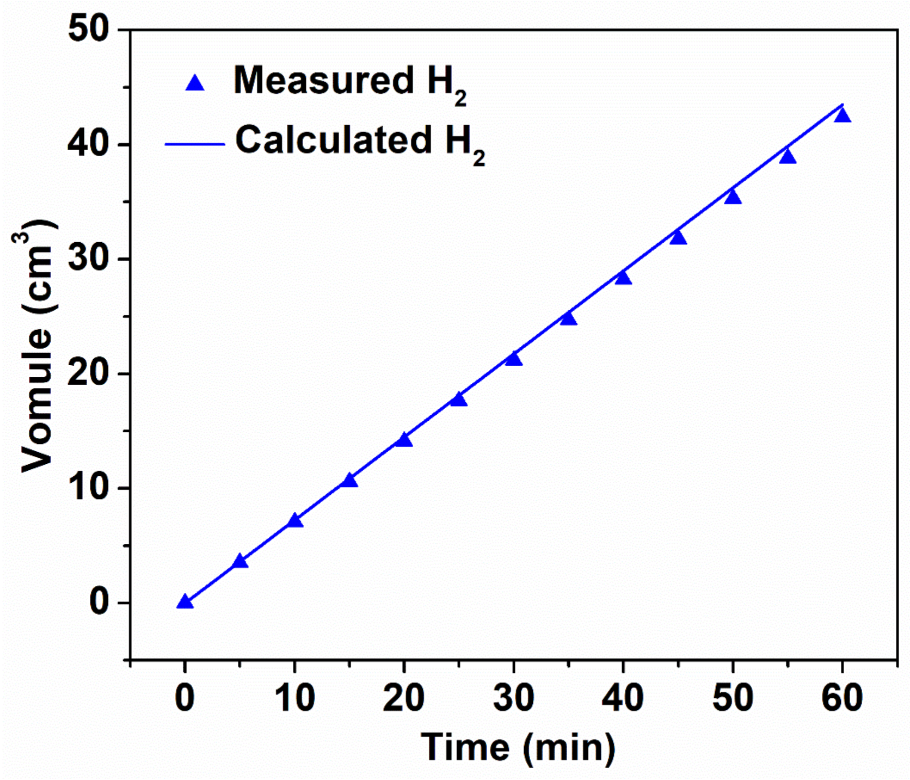

Supplementary Figure 49. Faraday efficiency. Generated and theoretical volumes of H_2 gas over time at 100 mA cm^{-2} for Ni/Yb_2O_3.

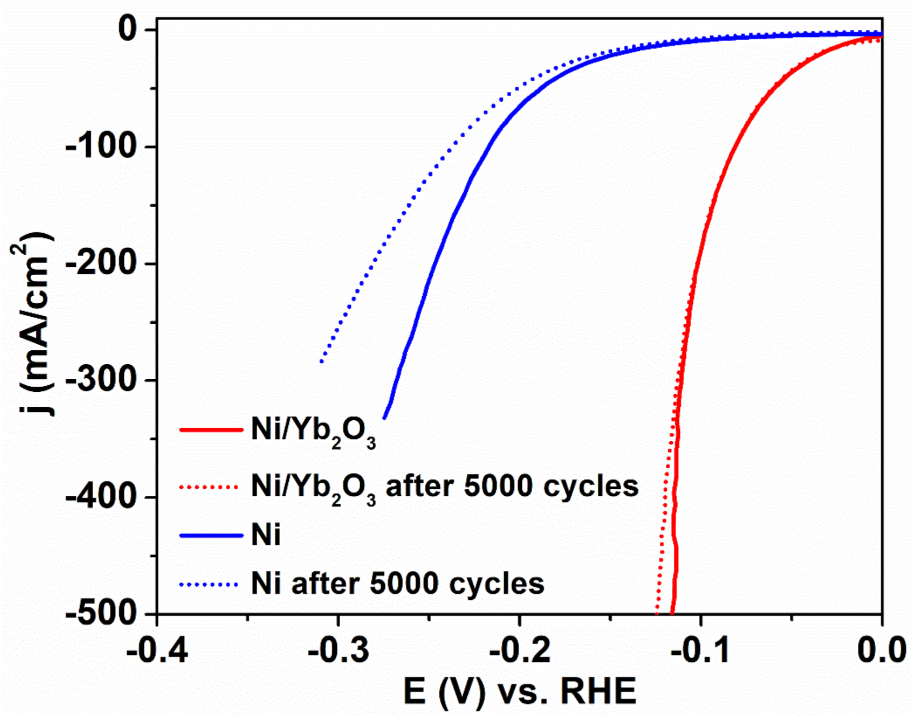

Supplementary Figure 50. Electrocatalytic HER stability. Polarization curves of Ni and Ni/Yb_2O_3 electrodes before and after 5000 cycles at a scan rate of 100 mV s^{-1}.

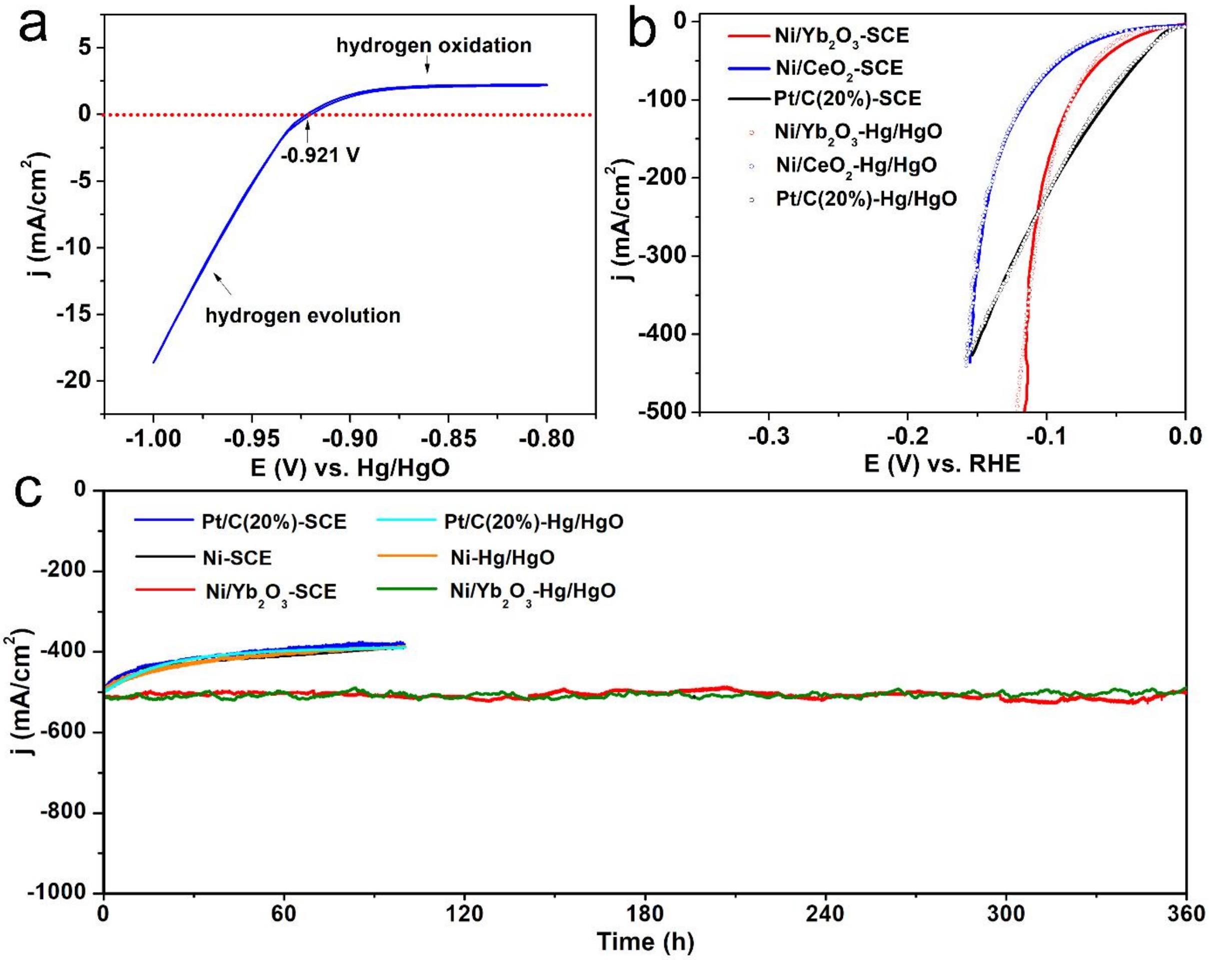

Supplementary Figure 51. HER performances of Ni/Yb_2O_3, Ni, Ni/CeO_2 and Pt/C(20%) tested by using different reference electrodes. a CV curves of platinum plate electrode recorded at a scan rate 5 mV s^{-1} for potential calibration of reference electrode, with the CV result of RHE calibration $E_{(RHE)} = E_{(Hg/HgO)} + 0.921$ V. **b** LSV polarization curves. **c** Chronopotentionmetric curves.

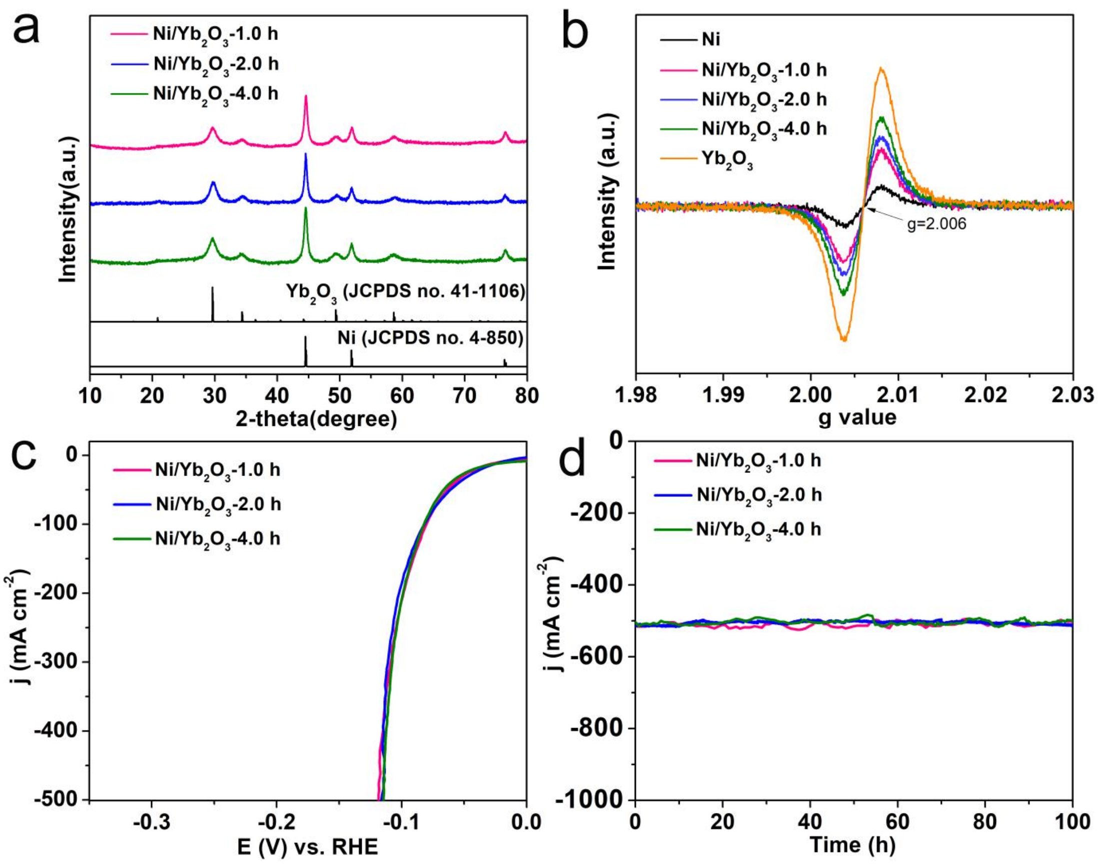

Supplementary Figure 52. Characterization and electrocatalytic HER performances of Ni/Yb_2O_3 catalysts with different oxygen vacancy concentrations (Ni/Yb_2O_3-1.0 h, Ni/Yb_2O_3-2.0 h and Ni/Yb_2O_3-4.0 h). a XRD patterns. **b** Electron paramagnetic resonance (EPR) spectra. **c** Polarization curves. **d** Chronopotentionmetric curves.

Due to the hydrogen reducing atmosphere in the preparation process, the oxygen atoms in Yb_2O_3 can be taken from the lattice by hydrogen to form oxygen vacancies. The electron paramagnetic resonance (EPR) spectra of Ni/Yb_2O_3 show the signal of oxygen vacancies at g = 2.006. The obviously higher signal of Yb_2O_3 than Ni indicates that the oxygen vacancies mainly come from Yb_2O_3. By adjusting the hydrogen sintering time, the Ni/Yb_2O_3 hybrids with different oxygen vacancy concentrations were prepared, as proved by the EPR spectra.

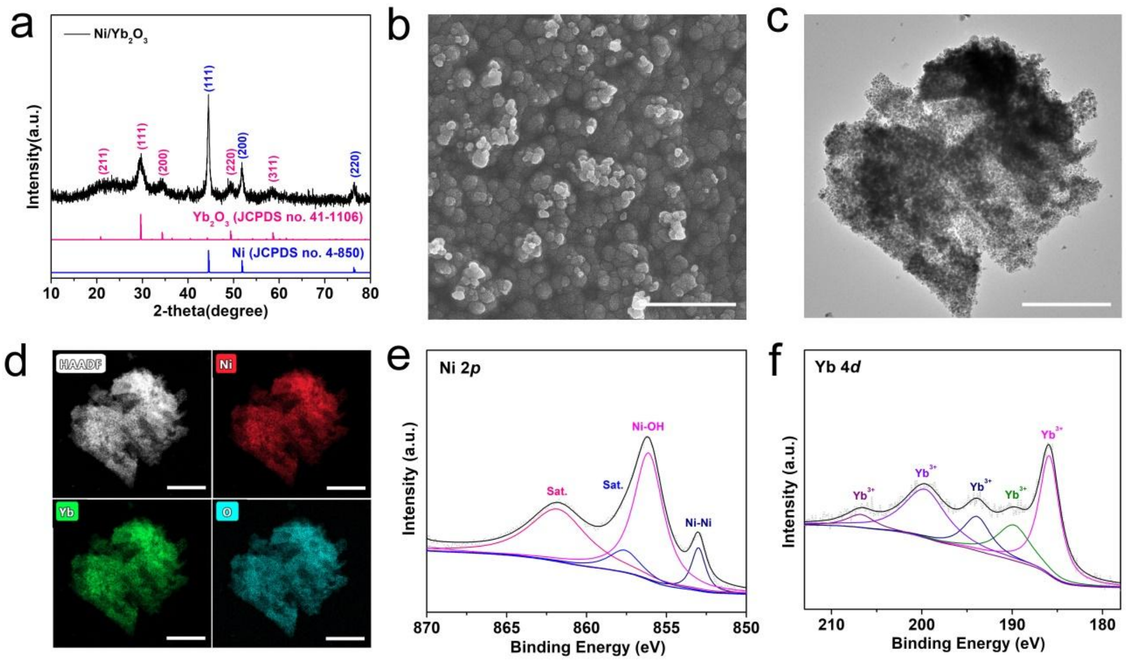

Supplementary Figure 53. Characterization of used Ni/Yb_2O_3 electrode after HER test. **a** XRD pattern. **b** SEM image (scale bar: 500 nm). **c** TEM image (scale bar: 500 nm). **d** TEM-EDX elemental mapping (scale bar: 500 nm). **e** Ni 2*p* XPS spectrum. **f** Yb 4*d* XPS spectrum.

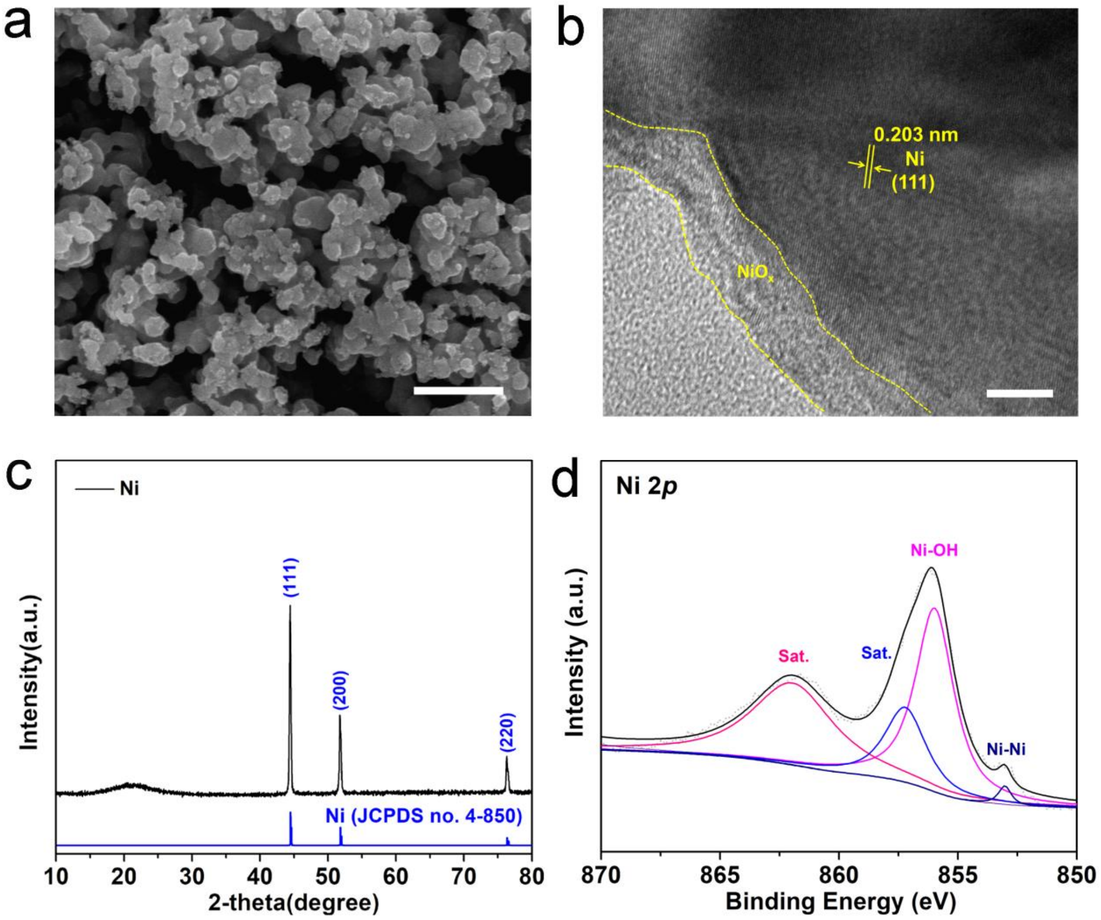

Supplementary Figure 54. Characterization of used Ni electrodes after HER test. a SEM image (scale bar: 500 nm). **b** TEM image (scale bar: 5 nm). **c** XRD pattern. **d** Ni 2*p* XPS spectrum.

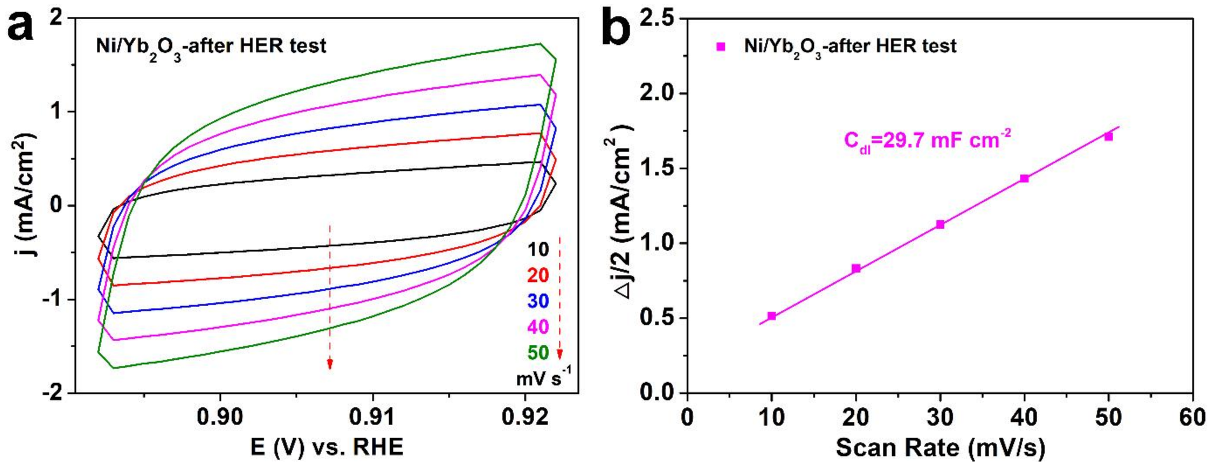

Supplementary Figure 55. ECSA by non-faradaic double layer capacitance obtained in 1.0 M KOH. a CV curves of Ni/Yb_2O_3 after HER test collected at various scan rates ranging from 10 to 50 mV s^{-1}. **b** Corresponding linear fitting of scan rates versus difference between the anodic and cathodic currents at -0.155 V.

$$A_{\mathrm{ECSA}}^{\mathrm{Ni/Yb_2O_3-after\ HER}} = \frac{29.7\ \mathrm{mF\ cm^{-2}}}{40\ \mu\mathrm{F\ cm^{-2}\ per\ cm_{ECSA}^2}} = 742.5\ \mathrm{cm_{ECSA}^2}$$

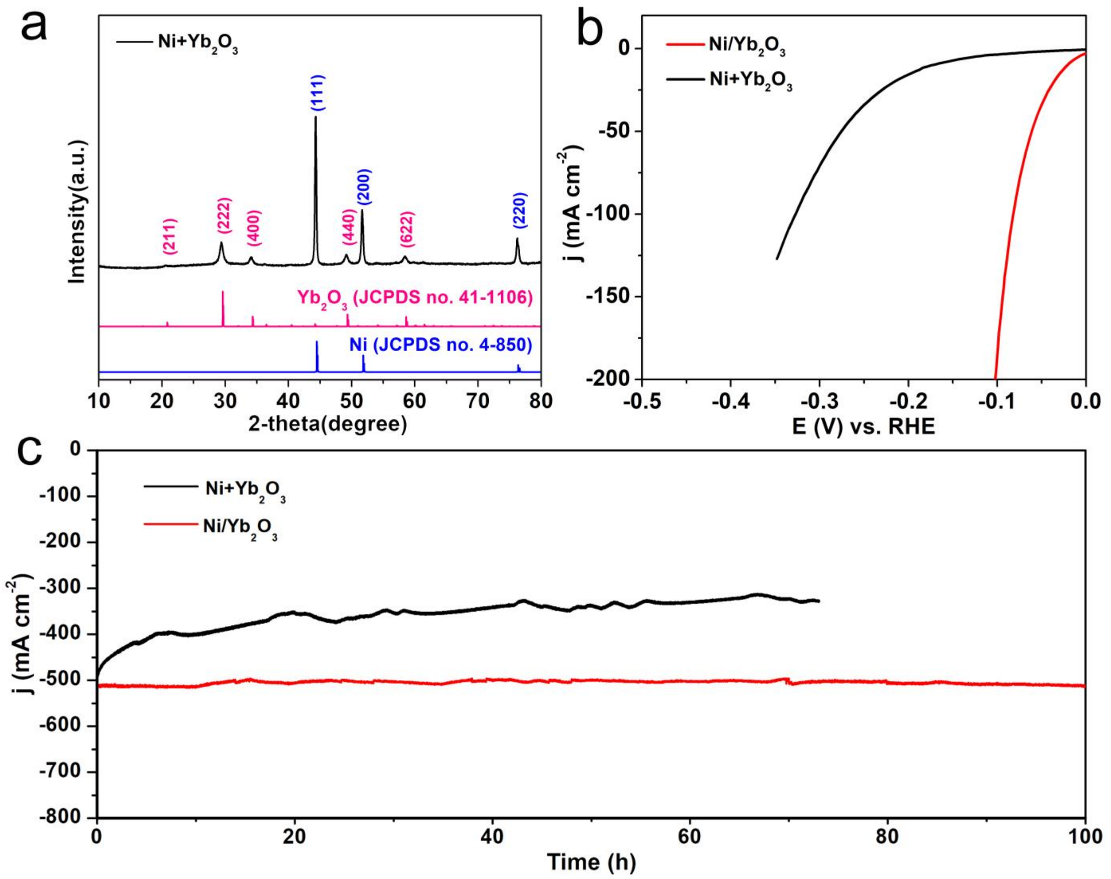

Supplementary Figure 56. Characterization and electrocatalytic HER performances of the Ni+Yb_2O_3 electrode. a XRD patterns. **b** Polarization curves. **c** Chronopotentionmetric curves.

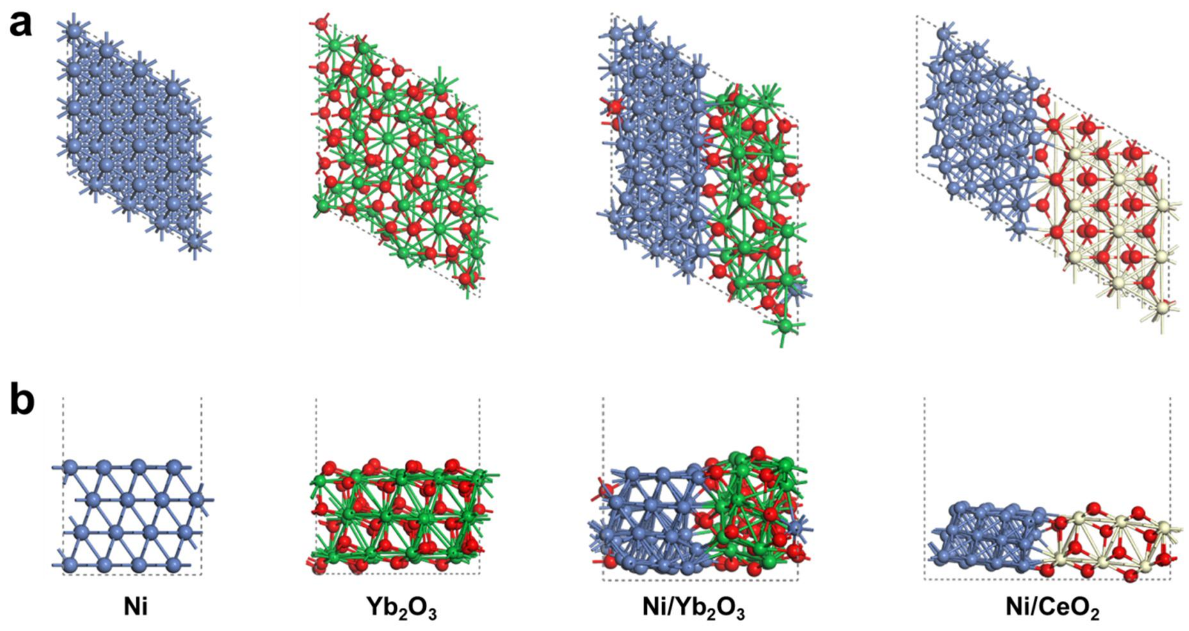

Supplementary Figure 57. DFT calculations of optimized structures for Ni, Yb_2O_3, Ni/Yb_2O_3, and Ni/CeO_2. a Top view. **b** Side view. The blue, red, green, and buff spheres represent the Ni, O, Yb, and Ce atoms, respectively.

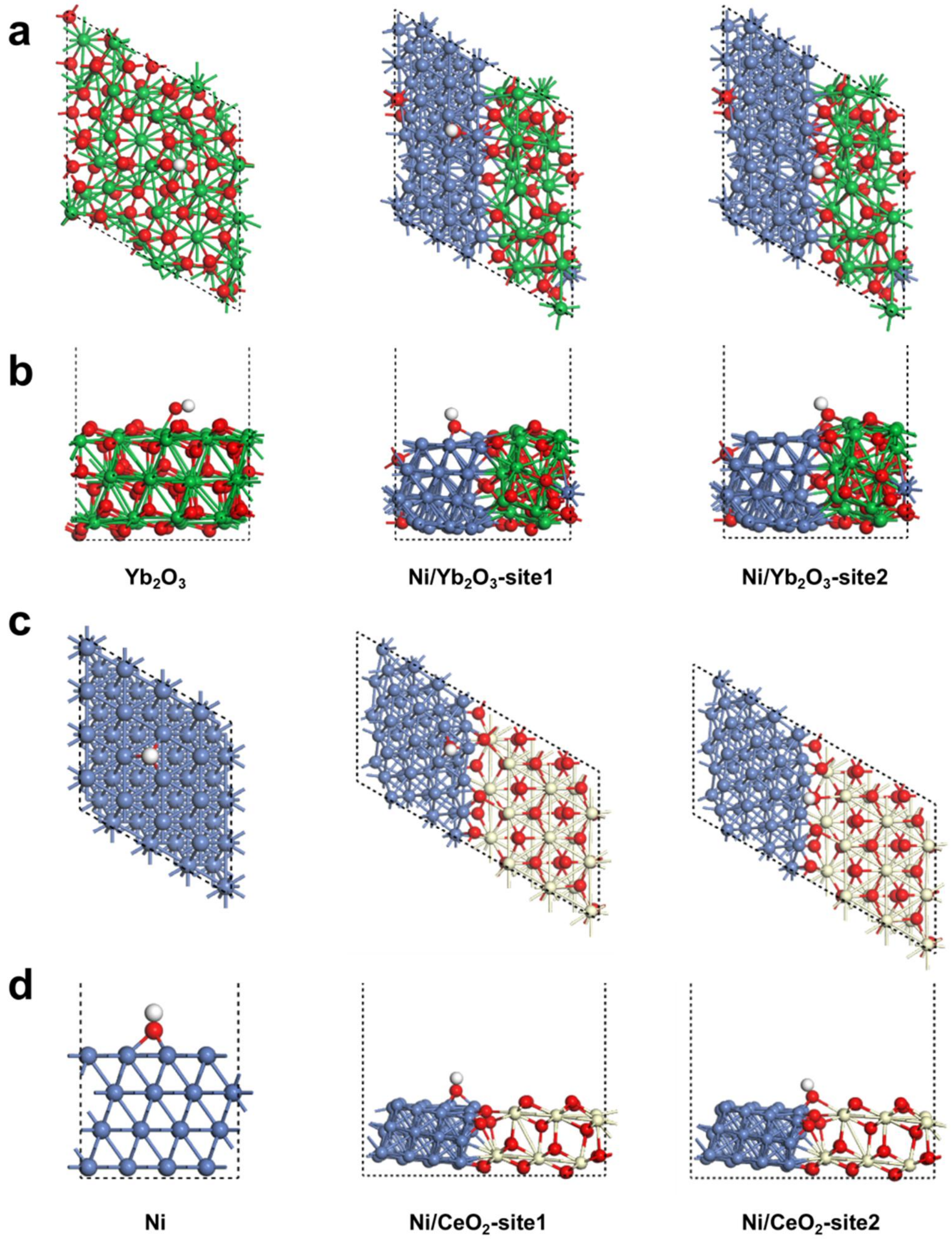

Supplementary Figure 58. Adsorption configurations of OH on optimized sites of different samples. a Top view for Yb_2O_3(222) and Ni(111)/Yb_2O_3(222). **b** Side view for Yb_2O_3(222) and Ni(111)/Yb_2O_3(222). **c** Top view for Ni(111) and Ni(111)/CeO_2(111). **d** Side view for Ni(111) and Ni(111)/CeO_2(111). The blue, red, white, green, and buff spheres represent the Ni, O, H, Yb, and Ce atoms, respectively.

Based on the results of DFT calculation, the adsorption energies of OH on the surface of Yb_2O_3(222), Ni(111)/Yb_2O_3(222)-site1 and Ni(111)/Yb_2O_3(222)-site2 are -0.24 eV, -0.12 eV and -0.54 eV, respectively. And those on the surface of Ni(111), Ni(111)/CeO_2(111)-site1 and Ni(111)/CeO_2(111)-site2 are -0.06 eV, -0.52 eV and -0.34 eV, respectively. The results reveal that the adsorption energy of OH on the surface of Ni(111) is significantly weaker than those on the surfaces of Yb_2O_3(222), Ni(111)/Yb_2O_3(222) and Ni(111)/CeO_2(111).

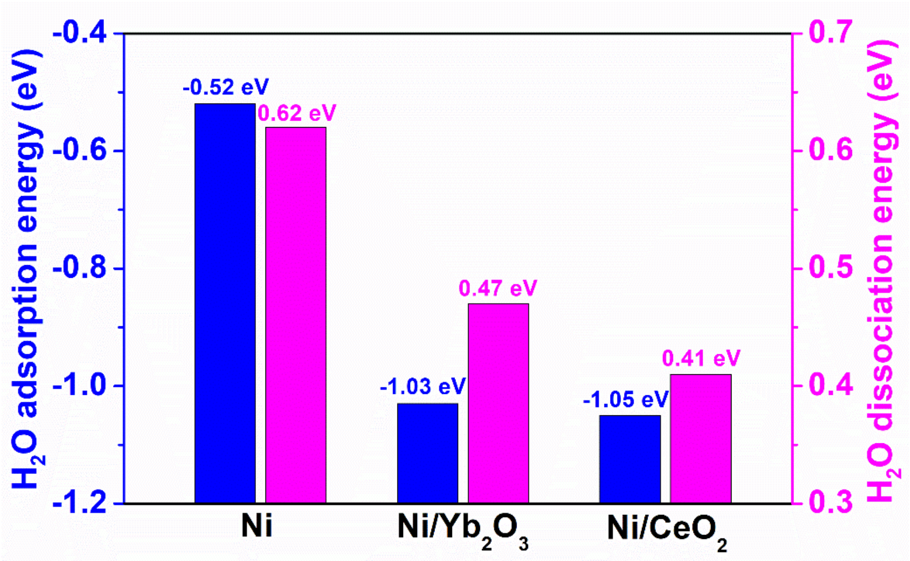

Supplementary Figure 59. Comparison of H_2O adsorption energy and dissociation energy on Ni, Ni/Yb_2O_3 and Ni/CeO_2. DFT-calculated adsorption energy of H_2O and energy barriers of H_2O dissociation on the surfaces of Ni(111), Ni(111)/Yb_2O_3(222), and Ni(111)/CeO_2(111).

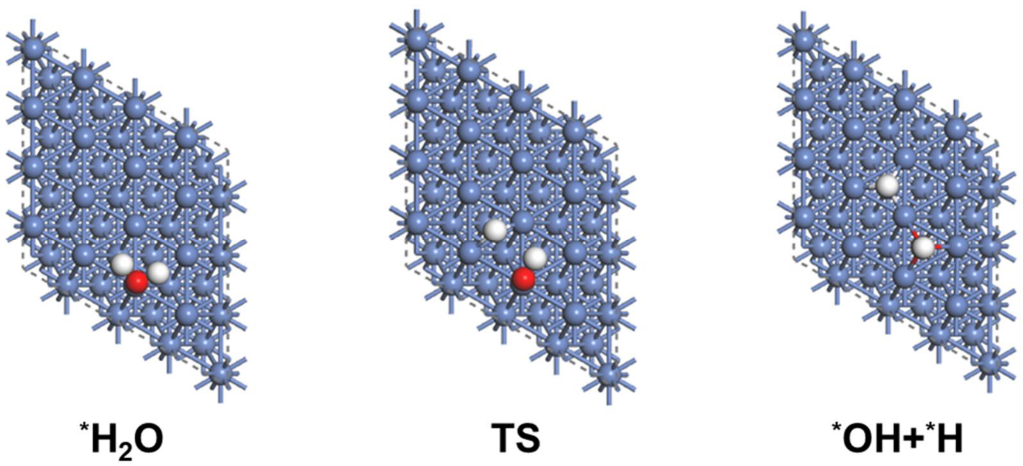

Supplementary Figure 60. Atomic configurations of simulated H_2O dissociation process on optimized sites of the pristine Ni(111) surface (top view). The blue, red, and white spheres represent the Ni, O, and H atoms, respectively.

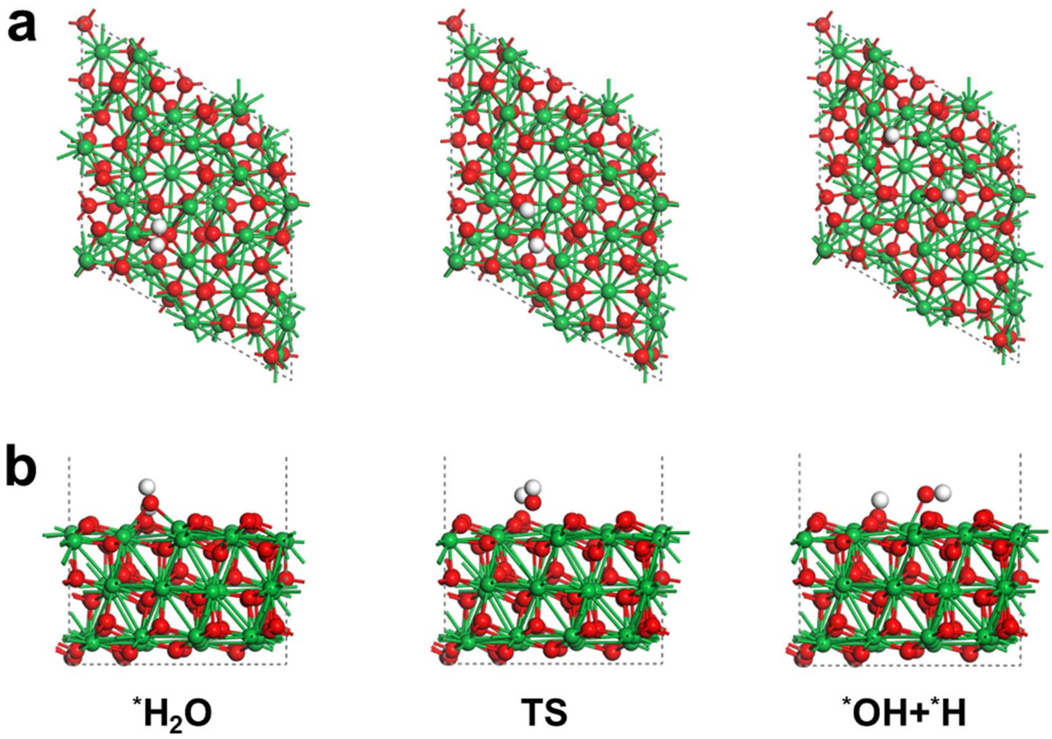

Supplementary Figure 61. Atomic configurations of simulated H_2O dissociation process on optimized sites of the pristine Yb_2O_3(222) surface. a Top view. **b** Side view. The red, white and green spheres represent the O, H and Yb atoms, respectively.

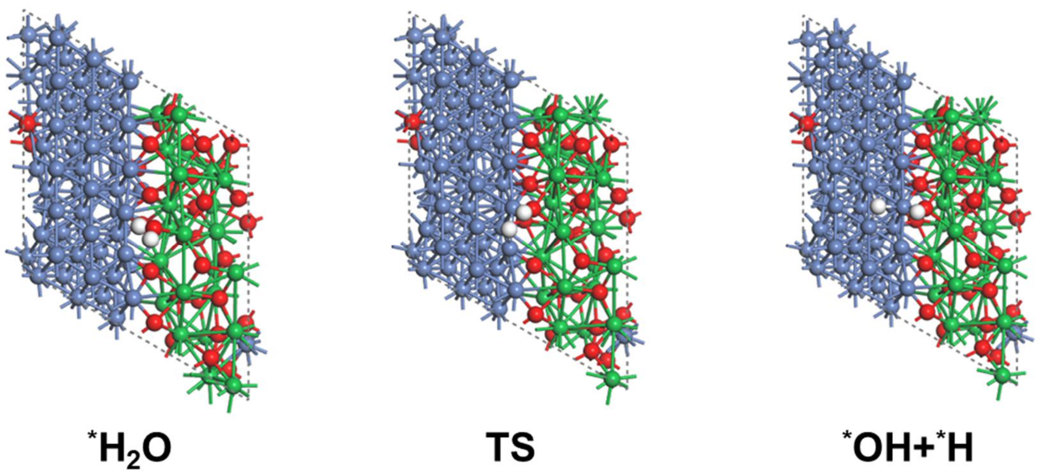

Supplementary Figure 62. Atomic configurations of simulated H_2O dissociation process on optimized sites of the Ni(111)/Yb_2O_3(222) interface (top view). The blue, red, white and green spheres represent the Ni, O, H and Yb atoms, respectively.

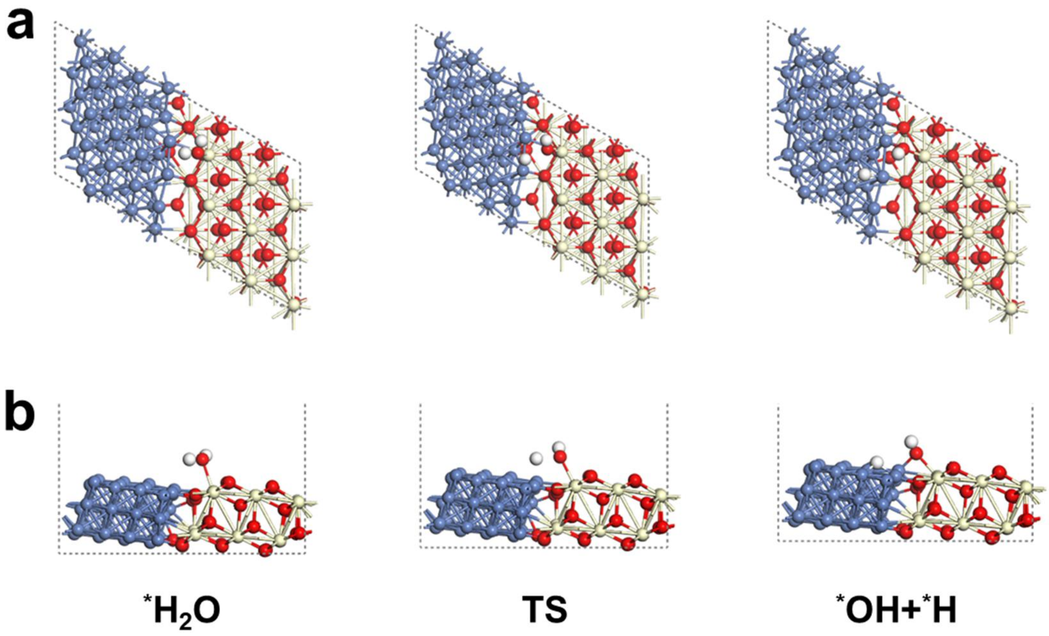

Supplementary Figure 63. Atomic configurations of simulated H_2O dissociation process on optimized sites of the Ni(111)/CeO_2(111) interface. a Top view. **b** Side view. The blue, red, white and buff spheres represent the Ni, O, H and Ce atoms, respectively.

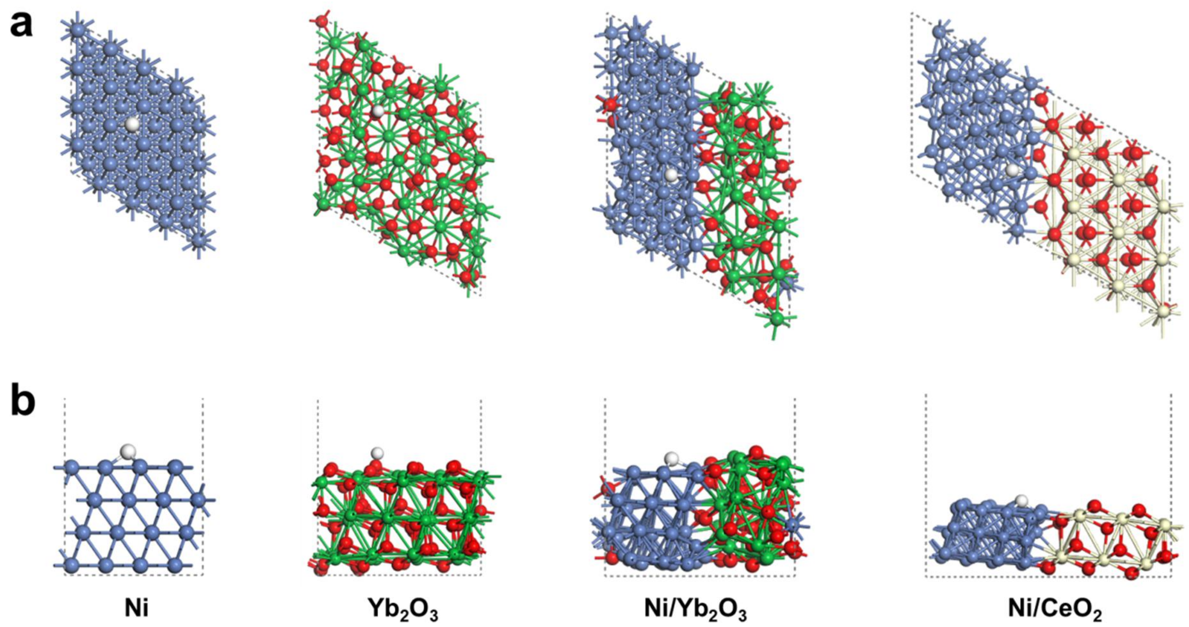

Supplementary Figure 64. Adsorption configurations of H* on the active sites of Ni(111), Yb_2O_3(222), Ni(111)/Yb_2O_3(222), and Ni(111)/CeO_2(111). a Top view. **b** Side view. The blue, red, white, green, and buff spheres represent the Ni, O, H, Yb, and Ce atoms, respectively.

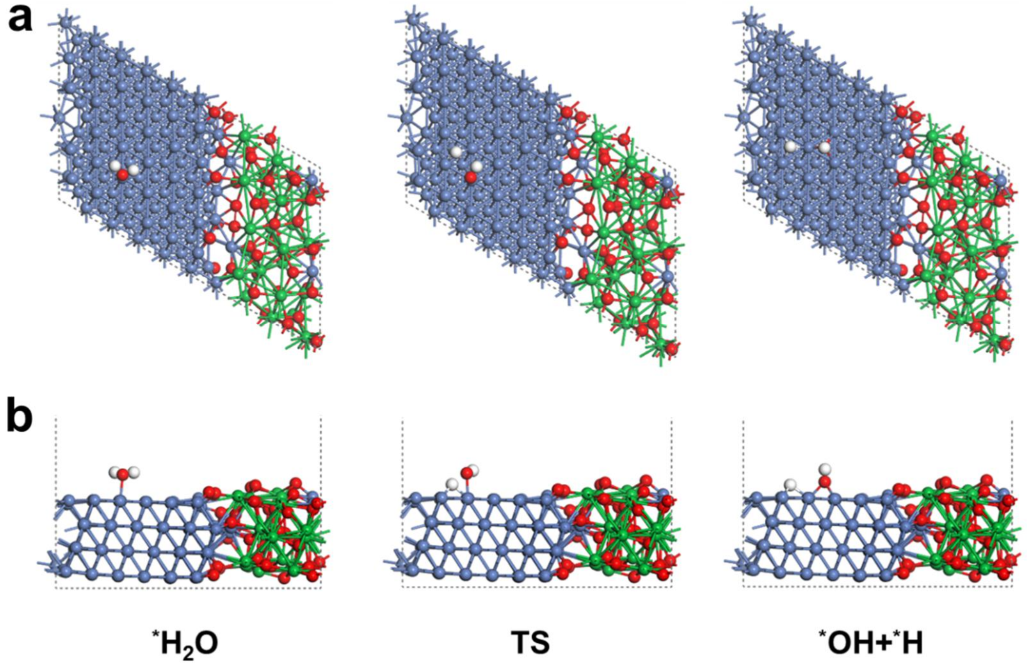

Supplementary Figure 65. Atomic configurations of simulated H_2O dissociation process on Ni sites far from the interface of Ni(111)/Yb_2O_3(222). a Top view. **b** Side view. The blue, red, white and green spheres represent the Ni, O, H and Yb atoms, respectively.

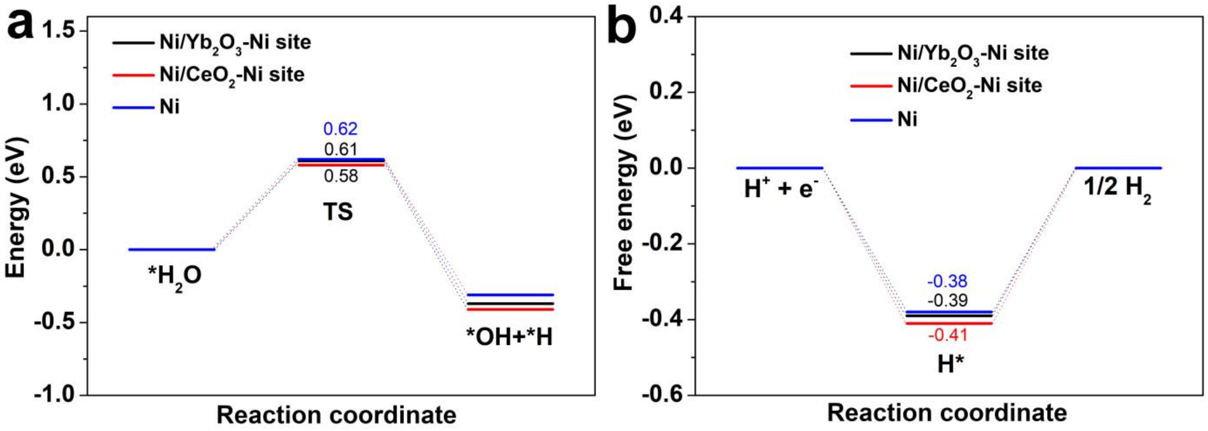

Supplementary Figure 66. DFT calculations. a DFT calculated reaction energy diagram of H_2O dissociation for bare Ni, Ni sites far from the interface of Ni/Yb_2O_3 (abbreviated as Ni/Yb_2O_3-Ni site) and Ni sites far from the interface of Ni/CeO_2 (abbreviated as Ni/CeO_2-Ni site). **b** Calculated ΔG_{H*} for bare Ni, Ni/Yb_2O_3-Ni site, and Ni/CeO_2-Ni site.

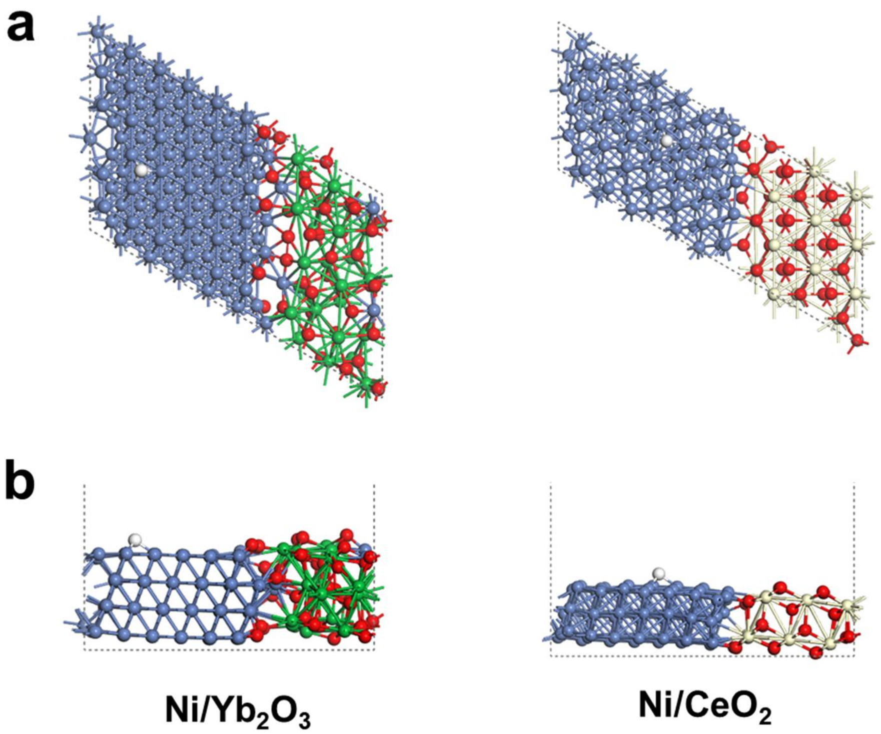

Supplementary Figure 67. Adsorption configurations of H* on the Ni sites far from the interfaces of Ni(111)/Yb_2O_3(222) and Ni(111)/CeO_2(111). a Top view. **b** Side view. The blue, red, white, green, and buff spheres represent the Ni, O, H, Yb, and Ce atoms, respectively.

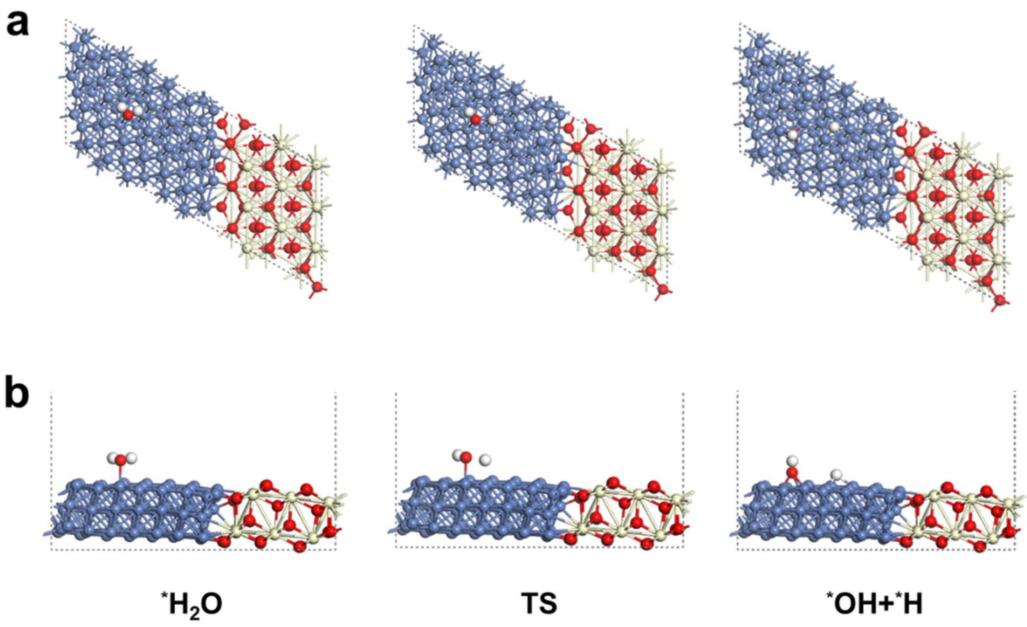

Supplementary Figure 68. Atomic configurations of simulated H_2O dissociation process on Ni sites far from the interface of Ni(111)/CeO_2(111). a Top view. **b** Side view. The blue, red, white and buff spheres represent the Ni, O, H and Ce atoms, respectively.

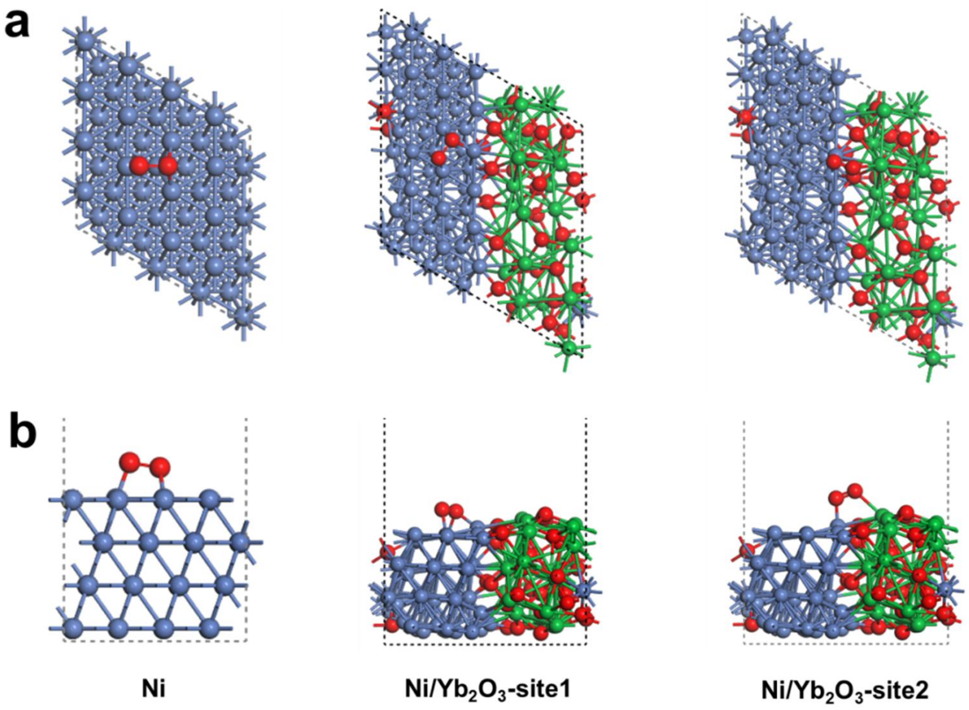

Supplementary Figure 69. Adsorption configurations of O_2 on the active sites of Ni(111) and Ni(111)/Yb_2O_3(222). a Top view. **b** Side view. The blue, red, and green spheres represent the Ni, O, and Yb atoms, respectively. The O_2 adsorption energy on Ni(111), Ni(111)/Yb_2O_3(222)-site1, and Ni(111)/Yb_2O_3(222)-site2 is -1.62 eV, -1.35 eV, and -0.98 eV, respectively.

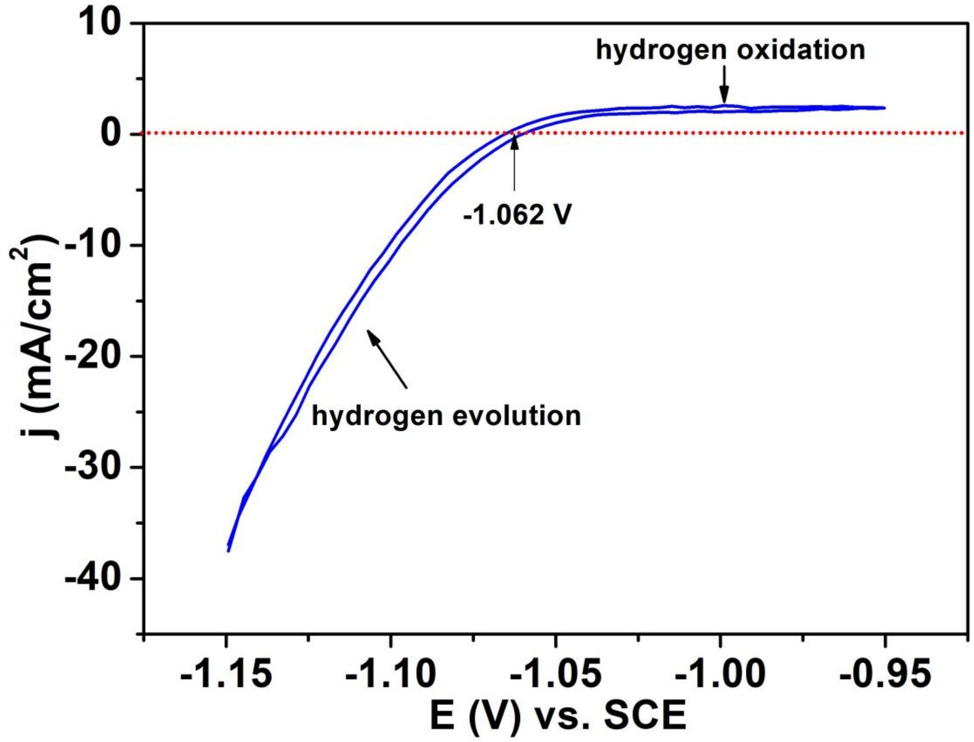

Supplementary Figure 70. Potential calibration of reference electrode in H_2-saturated 1.0 M KOH solution. CV curves of platinum plate electrode recorded at a scan rate 5 mV s^{-1}.

The CV result of RHE calibration: $E_{(RHE)} = E_{(SCE)} + 1.062$ V.

Supplementary Table 2. The fitted EIS parameters of Ni/Ln_2O_3 and Ni electrodes for HER.

The error bars represent the standard derivation based on triplicate measurements.

Overpotential / Sample	20 mV		40 mV		60 mV		80 mV		100 mV	
	R_p (Ω)	R_{ct} (Ω)	R_p (Ω)	R_{ct} (Ω)	R_p (Ω)	R_{ct} (Ω)	R_p (Ω)	R_{ct} (Ω)	R_p (Ω)	R_{ct} (Ω)
**Ni**		164.10 ± 3.80		78.37 ± 1.70		25.60 ± 0.90		17.42 ± 0.85		16.15 ± 2.13
**Ni/Sm_2O_3**	1.84 ± 0.08	124.77 ± 4.58	1.87 ± 0.10	71.88 ± 2.17	1.79 ± 0.16	19.10 ± 0.51	1.76 ± 0.15	3.32 ± 0.20	1.86 ± 0.08	2.10 ± 0.29
**Ni/Eu_2O_3**	2.04 ± 0.11	103.53 ± 7.32	1.88 ± 0.08	48.55 ± 1.42	1.95 ± 0.16	16.64 ± 1.39	1.82 ± 0.11	4.19 ± 0.12	1.74 ± 0.07	2.47 ± 0.12
**Ni/Gd_2O_3**	1.56 ± 0.11	102.93 ± 3.23	1.60 ± 0.07	46.95 ± 2.30	1.55 ± 0.18	6.25 ± 0.32	1.65 ± 0.14	2.22 ± 0.10	1.62 ± 0.12	1.31 ± 0.11
**Ni/Dy_2O_3**	1.85 ± 0.10	94.70 ± 1.18	1.71 ± 0.04	25.92 ± 1.96	1.52 ± 0.10	5.03 ± 0.76	1.58 ± 0.04	1.95 ± 0.13	1.63 ± 0.03	1.13 ± 0.10
**Ni/Ho_2O_3**	1.74 ± 0.06	72.43 ± 2.18	1.57 ± 0.11	17.22 ± 0.88	1.61 ± 0.13	3.89 ± 0.38	1.61 ± 0.18	1.59 ± 0.06	1.58 ± 0.11	1.02 ± 0.08
**Ni/Er_2O_3**	1.26 ± 0.08	61.30 ± 4.36	1.31 ± 0.10	15.32 ± 0.80	1.19 ± 0.15	2.99 ± 0.29	1.23 ± 0.10	1.29 ± 0.13	1.16 ± 0.05	0.85 ± 0.09
**Ni/Tm_2O_3**	1.23 ± 0.09	25.70 ± 1.85	1.16 ± 0.07	3.75 ± 0.29	1.18 ± 0.14	1.86 ± 0.27	0.93 ± 0.12	0.81 ± 0.12	0.94 ± 0.11	0.49 ± 0.06
**Ni/Yb_2O_3**	0.94 ± 0.12	13.40 ± 1.87	0.82 ± 0.11	2.85 ± 0.23	0.97 ± 0.16	1.69 ± 0.27	0.71 ± 0.04	0.64 ± 0.11	0.61 ± 0.96	0.37 ± 0.04
**Ni/Lu_2O_3**	1.21 ± 0.10	10.30 ± 1.01	1.11 ± 0.11	3.18 ± 0.13	0.98 ± 0.06	1.76 ± 0.15	0.89 ± 0.10	0.86 ± 0.07	0.82 ± 0.11	0.63 ± 0.10

Supplementary Table 3. Compositions of Ni/Yb_2O_3 electrodes with different Ni:Yb molar ratios determined by ICP-AES. The error bars represent the standard derivation based on triplicate measurements.

Sample	Ni (atom%)	Yb (atom%)
**Ni/Yb_2O_3-99:1**	98.85 ± 0.14	1.15 ± 0.14
**Ni/Yb_2O_3-97:3**	97.01 ± 0.29	2.99 ± 0.29
**Ni/Yb_2O_3-95:5**	94.89 ± 0.45	5.11 ± 0.45
**Ni/Yb_2O_3-90:10**	89.73 ± 1.16	10.27 ± 1.16
**Ni/Yb_2O_3-80:20**	79.21 ± 0.97	20.79 ± 0.97
**Ni/Yb_2O_3-70:30**	59.64 ± 1.45	30.36 ± 1.45
**Ni/Yb_2O_3-60:40**	59.31 ± 2.94	40.69 ± 2.94

Supplementary Table 4. EXAFS fitting parameters at the Ni K-edge and Yb L-edge.

Sample	Path	CN	R (Å)	σ^2 (10^{-3}Å^2)	ΔE_0 (eV)	R-factor
**Ni**	Ni–Ni	5.6 ± 0.2	2.48 ± 0.01	5.2 ± 0.2	5.5 ± 0.4	0.001
**Ni/Yb_2O_3**	Ni–Ni	5.3 ± 0.3	2.48 ± 0.01	5.5 ± 0.4	6.2 ± 0.7	0.001
**Yb_2O_3**	Yb–O	4.6 ± 0.2	2.24 ± 0.01	7.4 ± 0.5	2.1 ± 1.1	0.011
**Ni/Yb_2O_3**	Yb–O	4.5 ± 0.3	2.24 ± 0.01	7.5 ± 1.3	2.9 ± 1.0	0.013

CN: coordination number; R: bonding distance; σ^2: Debye-Waller factor; ΔE_0: inner potential shift; R factor: goodness of fit.

Supplementary Table 5. Comparison of the activities for Pt/C(20%) electrodes used in this work with those reported.

Catalyst	Electrolyte	Current density	Overpotential at corresponding j
**20 wt.% Pt/C**[6]	1 M KOH	100 mA cm^{-2}	205 mV
**20 wt.% Pt/C**[7]	1 M KOH	10 mA cm^{-2}	50 mV
**20 wt.% Pt/C**[8]	1 M KOH	200 mA cm^{-2}	~160 mV
**20 wt.% Pt/C**[9]	1 M KOH	10 mA cm^{-2}	13 mV
**20 wt.% Pt/C**[10]	1 M KOH	10 mA cm^{-2}<br>100 mA cm^{-2}	36 mV<br>99 mV
**20 wt.% Pt/C**[11]	1 M KOH	10 mA cm^{-2}	330 mV
**20 wt.% Pt/C**[12]	1 M KOH	10 mA cm^{-2}	10 mV
**20 wt.% Pt/C**[this work]	1 M KOH	10 mA cm^{-2}<br>100 mA cm^{-2}<br>200 mA cm^{-2}	10 mV<br>56 mV<br>96 mV

Supplementary Table 6. Comparison of the electrocatalytic HER activities for Ni/Yb_2O_3 and reported nonprecious catalysts in 1.0 M KOH electrolyte.

Catalyst	Substrate	Overpotential (mV) at 10 mA cm^{-2}	Tafel slope (mV dec^{-1})	Stability
**Ni_3N-V_2O_3**[13]	Ni foam	57	50	-13 mA cm^{-2}@24 h
**$NiP_2/NiSe_2$**[14]	Carbon fibers	89	65.7	-10 mA cm^{-2}@90 h
**$NiSe_2$**[15]	Carbon paper	157	76	-10 mA cm^{-2}@24 h
**Mo-NiO/Ni**[16]	Glassy carbon	50	86	-10 mA cm^{-2}@12 h
**Ni/Fe_3C**[17]	Ni foam	93	63	-10 mA cm^{-2}@20 h
**Mo-Ni_3S_2/Ni_xP_y**[18]	Ni foam	109	68.4	-10 mA cm^{-2}@24 h
**Li_xNiO/Ni**[11]	Ni foam	36	50	-20 mA cm^{-2}@50 h
**Ni_5P_4-Ru**[19]	Carbon cloth	54	52	-30 mA cm^{-2}@60 h
**$Ni/Ni(OH)_2$**[20]	Carbon paper	77	53	-10 mA cm^{-2}@10 h
**Ni/CeO_2**[21]	Glassy carbon	100	---	-30 mA cm^{-2}@100 h
**Co-NiS_2**[22]	Glassy carbon	80	43	-10~100 mAcm^{-2}@90h
**$Cu_{0.4}In_{0.6}NNi_3$**[23]	Carbon paper	42	51	-100mA cm^{-2}@60 h
**$Ni_3(BO_3)^{2}$-Ni_3S_2**[24]	Ni foam	92	152.3	-10 mA cm^{-2}@10 h
**Ni-N_x species**[25]	Glassy carbon	147	114	-30 mA cm^{-2}@10 h
**Ni-Co-P**[26]	Glassy carbon	58	57	-10 mA cm^{-2}@24 h
**Cu NDs/Ni_3S_2NTs**[27]	Carbon fiber	128	76.2	-85 mAcm^{-2}@30 h
**N-$NiCo_2S_4$**[28]	Ni foam	41	37	-10 mA cm^{-2}@1000 h
**C-$Ni_{1-x}O$**[9]	Ni foam	27	36	5000 cycles
**Ni(Cu)VO_x**[29]	Ni foam	21	28	-100 mA cm^{-2}@100 h
**Ni/NiO-3.8**[30]	Carbon substrate	90	41	5000 cycles
**Ni-Ni_3C**[31]	Carbon cloth	98	88.5	-35 mA cm^{-2}@35 h
**1T-$MoS_2/Ni(OH)_2$**[32]	Carbon cloth	57	30	-500 mA cm^{-2}@100 h

**$Ni(OH)_2$-$NiMoO_x$**[33]	Ni foam	36	38	2000 CV cycles
**$Co(OH)_2$-MoS_2**[34]	Glassy carbon	89	53	-10 mA cm^{-2}@20 h
**$Pt/Ni(HCO_3)_2$**[35]	Glassy carbon	44	40	---
**Ni_5P_4@$NiCo_2O_4$**[36]	Ni foam	27	27	-20 mA cm^{-2}@45 h
**$MoS_2/CoNi_2S_4$**[37]	Carbon paper	78	67	-10 mA cm^{-2}@48 h
**P-Ni_2P/Ru NCs**[38]	Carbon cloth	45	54.2	-10 mA cm^{-2}@100 h
**CoP-CeO_2**[39]	Ti mesh	43	45	-10 mA cm^{-2}@10 h
**Co_4N-CeO_2/GP**[40]	Graphite plate	24	61	-500 mA cm^{-2}@50 h
**NiCo-CeO_2/GP**[41]	Graphite plate	34	49.1	-500 mA cm^{-2}@48 h
**V-CoP@a-CeO_2**[10]	Carbon cloth	68	48.1	-20 mA cm^{-2}@60 h
**Cr-Co_4N**[42]	Carbon cloth	21	38.1	-100 mA cm^{-2}@10 h
**$CoW(OH)_x$**[43]	Ni foam	73.6	149.5	-20 mA cm^{-2}@70 h
**PtRu NCs/BP**[44]	Glassy carbon	22	19	-10 mA cm^{-2}@20 h
**RuAu SAAs**[45]	Glassy carbon	24	37	-10 mA cm^{-2}@10 h
**FeCoNiAlTi**[46]	---	88.2	40.1	-100 mA cm^{-2}@40 h
**CoP nanosheets**[47]	Carbon cloth	51	46	-10 mA cm^{-2}@20 h
**CoP/NiCoP**[6]	Ti foil	133	88	-20 mA cm^{-2}@24 h
**V-Co_4N**[48]	Ni foam	37	44	-20 mA cm^{-2}@27 h
**MoO_2/Ni**[49]	Ni foam	50.48	65.52	-10 mA cm^{-2}@30 h
**Ni/NiS**[50]	Carbon cloth	155	135	-100 mA cm^{-2}@40 h
**Ni/La-Ce oxide**[51]	Glassy carbon	114	72.7	-10~40 mA cm^{-2}@40 h
**Cu_3P@Ni**[52]	Copper foam	42	41	-50 mA cm^{-2}@20 h
**Ni_3N/Ni**[53]	FTO glass	89	---	-10 mA cm^{-2}@72 h
**Ni-$Ni(OH)_2$**[54]	Ni foam	72	43	-10 mA cm^{-2}@28 h
**$NiCu_x/Ni_3S_2$**[55]	Ti mesh	202	86	-10 mA cm^{-2}@11 h
**Ni/V_2O_3**[56]	Ni foam	61	79.7	-10 mA cm^{-2}@12 h
**Ni/TiO_2**[57]	Ni foam	46	41.8	-10 mA cm^{-2}@24 h

**Ni/NiS$_x$**[58]	Ni foam	42	34.31	-100 mA cm^{-2}@16 h
**Ni-MoO$_2$@BC**[59]	Glassy carbon	169	63	-10 mA cm^{-2}@300 h
**Ni/NiFe-oxide**[60]	Ni foam	29	82	-10 mA cm^{-2}@24 h
**MoO$_2$-Ni**[61]	Carbon cloth	46	56.9	-10 mA cm^{-2}@36 h
**Ni$_3$N/Ni**[62]	Ni foam	12	---	-10 mA cm^{-2}@50 h
**MoNi$_4$/MoO$_2$**[12]	Ni foam	15	30	-200 mA cm^{-2}@10 h
**Ni/Yb$_2$O$_3$**[this work]	Graphite plate	20	44.6	-500 mA cm^{-2}@360 h

Supplementary References

1 Li, C. et al. Phase and composition controllable synthesis of cobalt manganese spinel nanoparticles towards efficient oxygen electrocatalysis. *Nat. Commun.* **6**, 7345 (2015).

2 Popczun, E. J. et al. Nanostructured nickel phosphide as an electrocatalyst for the hydrogen evolution reaction. *J. Am. Chem. Soc.* **135**, 9267-9270 (2013).

3 Pan, Y., Hu, G., Lu, J., Xiao, L. & Zhuang, L. $Ni(OH)_2$-Ni/C for hydrogen oxidation reaction in alkaline media. *J. Energy Chem.* **29**, 111-115 (2019).

4 Bao, L. et al. The effects of trace Yb doping on the electrochemical performance of Li-rich layered oxide. *ChemSusChem* **12**, 2294-2301(2019).

5 Kibsgaard, J. et al. Designing an improved transition metal phosphide catalyst for hydrogen evolution using experimental and theoretical trends. *Energy Environ. Sci.* **8**, 3022-3029 (2015).

6 Lin, Y. et al. Construction of CoP/NiCoP nanotadpoles heterojunction interface for wide pH hydrogen evolution electrocatalysis and supercapacitor. *Adv. Energy Mater.* **9**, 1901213 (2019).

7 Shi, H. et al. Spontaneously separated intermetallic Co_3Mo from nanoporous copper as versatile electrocatalysts for highly efficient water splitting. *Nat. Commun.* **11**, 2940 (2020).

8 Wang, Z. et al. Copper-nickel nitride nanosheets as efficient bifunctional catalysts for hydrazine-assisted electrolytic hydrogen production. *Adv. Energy Mater.* **9**, 900390 (2019).

9 Kou, T. et al. Carbon doping switching on the hydrogen adsorption activity of NiO for hydrogen evolution reaction. *Nat. Commun.* **11**, 590 (2020).

10 Yang, L., Liu, R. & Jiao, L. Electronic redistribution construction and modulation of interface engineering on CoP for enhancing overall water splitting. *Adv. Funct. Mater.* **30**, 1909618 (2020).

11 Lu, K. et al. Li_xNiO/Ni Heterostructure with strong basic lattice oxygen enables electrocatalytic hydrogen evolution with Pt-like activity. *J. Am. Chem. Soc.* **142**, 12613-12619 (2020).

12 Zhang, J. et al. Efficient hydrogen production on $MoNi_4$ electrocatalysts with fast water dissociation kinetics. *Nat. Commun.* **8**, 15437 (2017).

13 Zhou, P. et al. Boosting the electrocatalytic HER performance of Ni_3N-V_2O_3 via the interface coupling effect. *Appl. Catal. B-Environ.* **283**, 119590 (2021).

14 Yang, L., Huang, L., Yao, Y. & Jiao, L. In-situ construction of lattice-matching $NiP_2/NiSe_2$ heterointerfaces with electron redistribution for boosting overall water splitting. *Appl. Catal. B-Environ.* **282**,119584 (2020).

15 Zhai, L. et al. In situ phase transformation on nickel-based selenides for enhanced hydrogen evolution reaction in alkaline medium. *ACS Energy Lett.* **5**, 2483-2491 (2020).

16 Huang, J. et al. Boosting hydrogen transfer during Volmer reaction at oxides/metal nanocomposites for efficient alkaline hydrogen evolution. *ACS Energy Lett.* **4**, 3002-3010 (2019).

17 Yang, C. et al. Ni-activated transition metal carbides for efficient hydrogen evolution in acidic and alkaline solutions. *Adv. Energy Mater.* **10**, 2002260 (2020).

18 Luo, X. et al. Interface engineering of hierarchical branched Mo-doped Ni_3S_2/Ni_xP_y hollow heterostructure nanorods for efficient overall water splitting. *Adv. Energy Mater.* **10**, 1903891 (2020).

19 He, Q. et al. Achieving efficient alkaline hydrogen evolution reaction over a Ni_5P_4 catalyst incorporating single-atomic Ru sites. *Adv. Mater.* **32**, 1906972 (2020).

20 Dai, L. et al. Ultrathin Ni(0)-embedded $Ni(OH)_2$ heterostructured nanosheets with enhanced electrochemical overall water splitting. *Adv. Mater.* **32**, 1906915 (2020).

21 Weng, Z. et al. Metal/oxide interface nanostructures generated by surface segregation for electrocatalysis. *Nano Lett.* **15**, 7704-7710 (2015).

22 Yin, J. et al. Atomic arrangement in metal-doped NiS_2 boosts the hydrogen evolution reaction in alkaline media. *Angew. Chem. Int. Ed.* **58**, 18676-18682 (2019).

23 Zhang, J. et al. Composition-tunable antiperovskite $Cu_xIn_{1-x}NNi_3$ as superior electrocatalysts for the hydrogen evolution reaction. *Angew. Chem. Int. Ed.* **59**, 1-7 (2020).

24 Sun, Z. et al. “Lewis base-hungry” amorphous-crystalline nickel borate nickel sulfide heterostructures by in situ structural engineering as effective bifunctional electrocatalysts toward overall water splitting. *ACS Appl. Mater. Interfaces* **12**, 23896-23903 (2020).

25 Lei, C. et al. Efficient alkaline hydrogen evolution on atomically dispersed Ni-N_x species anchored porous carbon with embedded Ni nanoparticles by accelerating water dissociation kinetics. *Energy Environ. Sci.* **12**, 149-156 (2019).

26 Fang, Z. et al. Dual tuning of Ni-Co-A (A = P, Se, O) nanosheets by anion substitution and holey engineering for efficient hydrogen evolution. *J. Am. Chem. Soc.* **140**, 5241-5247 (2018).

27 Feng, J. X., Wu, J. Q., Tong, Y. X. & Li, G. R. Efficient hydrogen evolution on Cu nanodots-decorated Ni_3S_2 nanotubes by optimizing atomic hydrogen adsorption and desorption. *J. Am. Chem. Soc.* **140**, 610-617 (2018).

28 Wu, Y. et al. Electron density modulation of $NiCo_2S_4$ nanowires by nitrogen incorporation for highly efficient hydrogen evolution catalysis. *Nat. Commun.* **9**, 1425 (2018).

29 Li, Y. et al. Implanting Ni-O-VO_x sites into Cu-doped Ni for low-overpotential alkaline hydrogen evolution. *Nat. Commun.* **11**, 2720 (2020).

30 Zhao, L. et al. Steering elementary steps towards efficient alkaline hydrogen evolution via size-dependent Ni/NiO nanoscale heterosurfaces. *Natl. Sci. Rev.* **7**, 27-36 (2020).

31 Wang, P. et al. Synergistic coupling of Ni nanoparticles with Ni_3C nanosheets for highly efficient overall water splitting. *Small* **16**, 2001642 (2020).

32 Chen, W. *et al.* Achieving rich and active alkaline hydrogen evolution heterostructures via interface engineering on 2D 1T-MoS_2 quantum sheets. *Adv. Funct. Mater.* **30**, 2000551 (2020).

33 Dong, Z., Lin, F., Yao, Y. & Jiao, L. Crystalline $Ni(OH)_2$/amorphous $NiMoO_x$ mixed-catalyst with Pt-like performance for hydrogen production. *Adv. Energy Mater.* **9**, 1902703 (2019).

34 Luo, Y. et al. Two-dimensional MoS_2 confined $Co(OH)_2$ electrocatalysts for hydrogen evolution in alkaline electrolytes. *ACS Nano* **12**, 4565-4573 (2018).

35 Lao, M. et al. Platinum/nickel bicarbonate heterostructures towards accelerated hydrogen evolution under alkaline conditions. *Angew. Chem. Int. Ed.* **58**, 5432-5437 (2019).

36 Zhang, T. et al. Nanometric Ni_5P_4 clusters nested on $NiCo_2O_4$ for efficient hydrogen production via alkaline water electrolysis. *Adv. Energy Mater.* **8**, 1801690 (2018).

37 Hu, J. et al. Kinetic-oriented construction of MoS_2 synergistic interface to boost pH-universal hydrogen evolution. *Adv. Funct. Mater.* **30**, 1908520 (2020).

38 Li, J. et al. Multiple-interface relay catalysis: enhancing alkaline hydrogen evolution through a combination of Volmer promoter and electrical-behavior regulation. *Chem. Eng. J.* **397**, 125457 (2020).

39 Zhang, R. et al. Selective phosphidation: an effective strategy toward CoP/CeO_2 interface engineering for superior alkaline hydrogen evolution electrocatalysis. *J. Mater. Chem. A.* **6**, 1985-1990 (2018).

40 Sun, H. et al. Boosting activity on Co_4N porous nanosheet by coupling CeO_2 for efficient electrochemical overall water splitting at high current densities. *Adv. Funct. Mater.* **30**, 1910596 (2020).

41 Sun, H. et al. Coupling NiCo alloy and CeO_2 to enhance electrocatalytic hydrogen evolution in alkaline solution. *Adv. Sustainable Syst.* **4**, 2000122 (2020).

42 Yao, N. et al. Synergistically tuning water and hydrogen binding abilities over Co_4N by Cr doping for exceptional alkaline hydrogen evolution electrocatalysis. *Adv. Energy Mater.* **9**, 1902449 (2019).

43 Zhang, L. et al. Accelerating neutral hydrogen evolution with tungsten modulated amorphous metal hydroxides. *ACS Catal.* **8**, 5200-5205 (2018).

44 Li, Y. et al. Hybrids of PtRu nanoclusters and black phosphorus nanosheets for highly efficient alkaline hydrogen evolution reaction. *ACS Catal.* **9**, 10870-10875 (2019).

45 Chen, C. H. et al. Ruthenium-based single-atom alloy with high electrocatalytic activity for hydrogen evolution. *Adv. Energy Mater.* **9**, 1803913 (2019).

46 Jia, Z. et al. A novel multinary intermetallic as an active electrocatalyst for hydrogen evolution. *Adv. Mater.* **32**, 2000385 (2020).

47 Dang, Y. et al. Constructing bifunctional 3D holey and ultrathin CoP nanosheets for efficient overall water splitting. *ACS Appl. Mater. Interfaces* **11**, 29879-29887 (2019).

48 Chen, Z. et al. Tailoring the d-band centers enables Co_4N nanosheets to be highly active for hydrogen evolution catalysis. *Angew. Chem. Int. Ed.* **130**, 5170-5174 (2018).

49 Liang, W. et al. Electron density modulation of MoO_2/Ni to produce superior hydrogen evolution and oxidation activities. *ACS Appl. Mater. Interfaces* **13**, 39470-39479 (2021).

50 Hegazy, M. B. Z. et al. Synergistic electrocatalytic hydrogen evolution in Ni/NiS

nanoparticles wrapped in multi-heteroatom-doped reduced graphene oxide nanosheets. *ACS Appl. Mater. Interfaces* **13**, 34043-34052 (2021).

51 Jang, M. J. et al. Promotion effect of modified Ni/C by La-Ce oxide for durable hydrogen evolution reaction. *ACS Sustainable Chem. Eng.* **9**, 12508-12513 (2021).

52 Chen, J., Li, X., Ma, B., Zhao, X. & Chen, Y. Cu_3P@Ni core-shell heterostructure with modulated electronic structure for highly efficient hydrogen evolution. *Nano Res.* **15**, 2935-2942 (2022).

53 Zhang, D. et al. Unconventional direct synthesis of Ni_3N/Ni with N-vacancies for efficient and stable hydrogen evolution. *Energy Environ. Sci.* **15**, 185-195 (2022).

54 Zhong, W. et al. Interfacial electron rearrangement: Ni activated $Ni(OH)_2$ for efficient hydrogen evolution. *J. Energy Chem.* **61**, 236-242 (2021).

55 Wang, J. et al. Enhanced hydrogen evolution activity of Ni/Ni_3S_2 nanosheet grown on Ti mesh by Cu doped Ni. *J. Electrochem. Soc.* **166**, 168-173 (2019).

56 Ming, M. et al. 3D nanoporous Ni/V_2O_3 hybrid nanoplate assemblies for highly efficient electrochemical hydrogen evolution. *J. Mater. Chem. A* **6**, 21452-21457 (2018).

57 Zhou, P. et al. Host dependent electrocatalytic hydrogen evolution of Ni/TiO_2 composites. *J. Mater. Chem. A* **9**, 6325-6334 (2021).

58 Xiao, L., Yao, P., Xue, T. & Li, F. One-step electrodeposition synthesis of Ni/NiS_x@NF catalyst on nickel foam (NF) for hydrogen evolution reaction. *Mol. Catal.* **511**, 11694 (2021).

59 Yusuf, B. A. et al. Ni nanoparticles oriented on MoO_2@BC nanosheets with an

outstanding long-term stability for hydrogen evolution reaction. *Chem. Eng. Sci.* **246**, 116868 (2021).

60 Tian, Y. et al. Two-dimensional hetero-nanostructured electrocatalyst of Ni/NiFe-layered double oxide for highly efficient hydrogen evolution reaction in alkaline medium. *Chem. Eng. J.* **426**, 131827 (2021).

61 Wang, B. et al. Electron-transfer enhanced MoO_2-Ni heterostructures as a highly efficient pH-universal catalyst for hydrogen evolution. *Sci. China. Chem.* **63**, 841-849 (2020).

62 Song, F. et al. Interfacing nickel nitride and nickel boosts both electrocatalytic hydrogen evolution and oxidation reactions. *Nat. Commun.* **9**, 4531 (2018).
